# Global dietary quality in 185 countries from 1990 to 2018 show wide differences by nation, age, education, and urbanicity

**DOI:** 10.1038/s43016-022-00594-9

**Published:** 2022-09-19

**Authors:** Victoria Miller, Patrick Webb, Frederick Cudhea, Peilin Shi, Jianyi Zhang, Julia Reedy, Josh Erndt-Marino, Jennifer Coates, Dariush Mozaffarian, Global Dietary Database, Murat Bas, Jemal Haidar Ali, Suhad Abumweis, Anand Krishnan, Puneet Misra, Nahla Chawkat Hwalla, Chandrashekar Janakiram, Nur Indrawaty Liputo, Abdulrahman Musaiger, Farhad Pourfarzi, Iftikhar Alam, Karin DeRidder, Celine Termote, Anjum Memon, Aida Turrini, Elisabetta Lupotto, Raffaela Piccinelli, Stefania Sette, Karim Anzid, Marieke Vossenaar, Paramita Mazumdar, Ingrid Rached, Alicia Rovirosa, María Elisa Zapata, Tamene Taye Asayehu, Francis Oduor, Julia Boedecker, Lilian Aluso, Johana Ortiz-Ulloa, J. V. Meenakshi, Michelle Castro, Giuseppe Grosso, Anna Waskiewicz, Umber S. Khan, Anastasia Thanopoulou, Reza Malekzadeh, Neville Calleja, Marga Ocke, Zohreh Etemad, Mohannad Al Nsour, Lydiah M. Waswa, Eha Nurk, Joanne Arsenault, Patricio Lopez-Jaramillo, Abla Mehio Sibai, Albertino Damasceno, Carukshi Arambepola, Carla Lopes, Milton Severo, Nuno Lunet, Duarte Torres, Heli Tapanainen, Jaana Lindstrom, Suvi Virtanen, Cristina Palacios, Eva Roos, Imelda Angeles Agdeppa, Josie Desnacido, Mario Capanzana, Anoop Misra, Ilse Khouw, Swee Ai Ng, Edna Gamboa Delgado, Mauricio Caballero, Johanna Otero, Hae-Jeung Lee, Eda Koksal, Idris Guessous, Carl Lachat, Stefaan De Henauw, Ali Reza Rahbar, Alison Tedstone, Androniki Naska, Angie Mathee, Annie Ling, Bemnet Tedla, Beth Hopping, Brahmam Ginnela, Catherine Leclercq, Charmaine Duante, Christian Haerpfer, Christine Hotz, Christos Pitsavos, Colin Rehm, Coline van Oosterhout, Corazon Cerdena, Debbie Bradshaw, Dimitrios Trichopoulos, Dorothy Gauci, Dulitha Fernando, Elzbieta Sygnowska, Erkki Vartiainen, Farshad Farzadfar, Gabor Zajkas, Gillian Swan, Guansheng Ma, Gulden Pekcan, Hajah Masni Ibrahim, Harri Sinkko, Helene Enghardt Barbieri, Isabelle Sioen, Jannicke Myhre, Jean-Michel Gaspoz, Jillian Odenkirk, Kanitta Bundhamcharoen, Keiu Nelis, Khairul Zarina, Lajos Biro, Lars Johansson, Laufey Steingrimsdottir, Leanne Riley, Mabel Yap, Manami Inoue, Maria Szabo, Marja-Leena Ovaskainen, Meei-Shyuan Lee, Mei Fen Chan, Melanie Cowan, Mirnalini Kandiah, Ola Kally, Olof Jonsdottir, Pam Palmer, Peter Vollenweider, Philippos Orfanos, Renzo Asciak, Robert Templeton, Rokiah Don, Roseyati Yaakub, Rusidah Selamat, Safiah Yusof, Sameer Al-Zenki, Shu-Yi Hung, Sigrid Beer-Borst, Suh Wu, Widjaja Lukito, Wilbur Hadden, Wulf Becker, Xia Cao, Yi Ma, Yuen Lai, Zaiton Hjdaud, Didier Garriguet, Jennifer Ali, Ron Gravel, Tina Tao, Jacob Lennert Veerman, Shashi Chiplonkar, Mustafa Arici, Le Tran Ngoan, Demosthenes Panagiotakos, Yanping Li, Antonia Trichopoulou, Noel Barengo, Anuradha Khadilkar, Veena Ekbote, Noushin Mohammadifard, Irina Kovalskys, Avula Laxmaiah, Harikumar Rachakulla, Hemalatha Rajkumar, Indrapal Meshram, Laxmaiah Avula, Nimmathota Arlappa, Rajkumar Hemalatha, Licia lacoviello, Marialaura Bonaccio, Simona Costanzo, Yves Martin-Prevel, Katia Castetbon, Nattinee Jitnarin, Yao-Te Hsieh, Sonia Olivares, Gabriela Tejeda, Aida Hadziomeragic, Amanda de Moura Souza, Wen-Harn Pan, Inge Huybrechts, Alan de Brauw, Mourad Moursi, Maryam Maghroun, Augustin Nawidimbasba Zeba, Nizal Sarrafzadegan, Lital Keinan-Boker, Rebecca Goldsmith, Tal Shimony, Irmgard Jordan, Shivanand C. Mastiholi, Moses Mwangi, Yeri Kombe, Zipporah Bukania, Eman Alissa, Nasser Al-Daghri, Shaun Sabico, Martin Gulliford, Tshilenge S. Diba, Kyungwon Oh, Sanghui Kweon, Sihyun Park, Yoonsu Cho, Suad Al-Hooti, Chanthaly Luangphaxay, Daovieng Douangvichit, Latsamy Siengsounthone, Pedro Marques-Vidal, Constance Rybak, Amy Luke, Nipa Rojroongwasinkul, Noppawan Piaseu, Kalyana Sundram, Donka Baykova, Parvin Abedi, Fariza Fadzil, Noriklil Bukhary Ismail Bukhary, Pascal Bovet, Sandjaja Sandjaja, Yu Chen, Norie Sawada, Shoichiro Tsugane, Lalka Rangelova, Stefka Petrova, Vesselka Duleva, Anna Karin Lindroos, Jessica Petrelius Sipinen, Lotta Moraeus, Per Bergman, Ward Siamusantu, Lucjan Szponar, Hsing-Yi Chang, Makiko Sekiyama, Balakrishna Nagalla, Kalpagam Polasa, Sesikeran Boindala, Khanh Le Nguyen Bao, Jalila El Ati, Daniel Illescas-Zarate, Luz Maria Sanchez-Romero, Ivonne Ramirez Silva, Juan Rivera Dommarco, Simon Barquera, Sonia Rodríguez-Ramírez, Nayu Ikeda, Sahar Zaghloul, Anahita Houshiar-rad, Fatemeh Mohammadi-Nasrabadi, Morteza Abdollahi, Khun-Aik Chuah, Zaleha Abdullah Mahdy, Alison Eldridge, Eric L. Ding, Herculina Kruger, Sigrun Henjum, Anne Fernandez, Milton Fabian Suarez-Ortegon, Nawal Al-Hamad, Veronika Janská, Reema Tayyem, Parvin Mirmiran, Roya Kelishadi, Eva Warensjo Lemming, Almut Richter, Gert Mensink, Lothar Wieler, Daniel Hoffman, Benoit Salanave, Cho-il Kim, Rebecca Kuriyan-Raj, Sumathi Swaminathan, Saeed Dastgiri, Sirje Vaask, Tilakavati Karupaiah, Fatemeh Vida Zohoori, Alireza Esteghamati, Sina Noshad, Maryam Hashemian, Elizabeth Mwaniki, Elizabeth Yakes-Jimenez, Justin Chileshe, Sydney Mwanza, Lydia Lera Marques, Alan Martin Preston, Samuel Duran Aguero, Mariana Oleas, Luz Posada, Angelica Ochoa, Khadijah Shamsuddin, Zalilah Mohd Shariff, Hamid Jan Bin Jan Mohamed, Wan Manan, Anca Nicolau, Cornelia Tudorie, Bee Koon Poh, Pamela Abbott, Mohammadreza Pakseresht, Sangita Sharma, Tor Strand, Ute Alexy, Ute Nöthlings, Jan Carmikle, Ken Brown, Jeremy Koster, Indu Waidyatilaka, Pulani Lanerolle, Ranil Jayawardena, Julie M. Long, K. Michael Hambidge, Nancy F. Krebs, Aminul Haque, Gudrun B. Keding, Liisa Korkalo, Maijaliisa Erkkola, Riitta Freese, Laila Eleraky, Wolfgang Stuetz, Inga Thorsdottir, Ingibjorg Gunnarsdottir, Lluis Serra-Majem, Foong Ming Moy, Simon Anderson, Rajesh Jeewon, Corina Aurelia Zugravu, Linda Adair, Shu Wen Ng, Sheila Skeaff, Dirce Marchioni, Regina Fisberg, Carol Henry, Getahun Ersino, Gordon Zello, Alexa Meyer, Ibrahim Elmadfa, Claudette Mitchell, David Balfour, Johanna M. Geleijnse, Mark Manary, Laetitia Nikiema, Tatyana El-kour, Masoud Mirzaei, Rubina Hakeem

**Affiliations:** 1 Friedman School of Nutrition Science and Policy, Tufts University, Boston, MA USA; 2 Department of Medicine, McMaster University, Hamilton, Ontario Canada; 3 Population Health Research Institute, Hamilton, Ontario Canada; 4 Division of General Internal Medicine and Primary Care, Brigham and Women’s Hospital, Boston, MA USA; 5 Acibadem University, Istanbul, Turkey; 6 Addis Ababa University, Addis Ababa, Ethiopia; 7 Al Ain University, Abu Dhabi, UAE; 8 All India Institute of Medical Sciences, New Delhi, India; 9 American University of Beirut, Beirut, Lebanon; 10 Amrita School of Dentistry, Ernakulam, India; 11 Andalas University, Padang, Indonesia; 12 Arab Center for Nutrition, Manama, Bahrain; 13 Ardabil University of Medical Sciences, Ardabil, Islamic Republic of Iran; 14 Bacha Khan University, Charsadda, Pakistan; 15 Belgian Public Health Institute, Brussels, Belgium; 16 Biodiversity International, Maccarese, Italy; 17 Brighton and Sussex Medical School, Brighton, UK; 18 CREA-Alimenti e Nutrizione, Rome, Italy; 19 Cadi Ayyad University, Ben Guerir, Morocco; 20 Center for Studies of Sensory Impairment, Aging and Metabolism (CeSSIAM), Guatemala City, Guatemala; 21 Centre For Media Studies, New Delhi, India; 22 Centro de Atencion Nutricional Antimano (CANIA), Miami, FL USA; 23 Centro de Estudios sobre Nutrición Infantil (CESNI), Buenos Aires, Argentina; 24 College of Applied Sciences, Department of Food Science and Applied Nutrition, Addis Ababa Science and Technology University, Addis Ababa, Ethiopia; 25 Consultative Group on International Agricultural Research (CGIAR), Montpellier, France; 26 Cuenca University, Cuenca, Ecuador; 27 Delhi School of Economics, University of Delhi, Delhi, India; 28 Departamento de Alimentacao Escolar, Sao Paulo, Brazil; 29 Department of Biomedical and Biotechnological Sciences, University of Catnia, Catania, Italy; 30 Department of CVD Epidemiology, Prevention and Health Promotion, Institute of Cardiology, Warsaw, Poland; 31 Department of Community Health Sciences, Aga Khan University, Karachi, Pakistan; 32 Diabetes Center, 2nd Department of Internal Medicine, Athens University, Athens, Greece; 33 Digestive Disease Research Institute, Tehran University of Medical Sciences, Tehran, Islamic Republic of Iran; 34 Directorate for Health Information & Research, Tarxien, Malta; 35 Dutch National Institute for Public Health and the Environment (RIVM), Bilthoven, Netherlands; 36 Eastern Mediterranean Public Health Network (EMPHNET), Amman, Jordan; 37 Egerton University, Njoro, Kenya; 38 Estonian National Institute for Health Development, Tallinn, Estonia; 39 FHI Solutions, Washington, DC USA; 40 FOSCAL and UDES, Bucaramanga, Colombia; 41 Faculty of Health Sciences, American University of Beirut, Beirut, Lebanon; 42 Faculty of Medicine, Eduardo Mondlane University, Maputo, Mozambique; 43 Faculty of Medicine, University of Colombo, Colombo 5, Sri Lanka; 44 Faculty of Medicine – Institute of Public Health, University of Porto, Porto, Portugal; 45 Faculty of Nutrition and Food Sciences, University of Porto, Porto, Portugal; 46 Finnish Institute for Health and Welfare, Helsinki, Finland; 47 Florida International University, Miami, FL USA; 48 Folkhälsan Research Center, Helsinki, Finland; 49 Food and Nutrition Research Institue (DOST-FNRI), Manila, Philippines; 50 Food and Nutrition Research Institue (DOST-FNRI), Taguig City, Philippines; 51 Fortis CDOC Center for Excellence for Diabetes, New Delhi, India; 52 FrieslandCampina, Amersfoort, The Netherlands; 53 Fundacion Cardiovascular de Colombia, Bucaramanga, Colombia; 54 Fundacion INFANT and Consejo Nacional De Investigaciones Cientificas y Tecnicas (CONICET), Buenos Aires, Argentina; 55 Fundacion Oftalmologica de Santander (FOSCAL), Floridablanca, Colombia; 56 Gachon University, Seongnam-si, South Korea; 57 Gazi University, Ankara, Turkey; 58 Geneva University Hospitals, Geneva, Switzerland; 59 Ghent University, Ghent, Belgium; 60 Global Dietary Database Consortium, Boston, MA USA; 61 Government of Canada, Statistics Canada, Ottawa, Ontario Canada; 62 Griffith University, Gold Coast, Queensland Australia; 63 HC Jehangir Medical Research Institute, Pune, India; 64 Hacettepe University Faculty of Medicine, Ankara, Turkey; 65 Hanoi Medical University, Hanoi, Vietnam; 66 Harokopio University, Athens, Greece; 67 Harvard School of Public Health, Cambridge, MA USA; 68 Hellenic Health Foundation and University of Athens, Athens, Greece; 69 Herbert Wetheim College of Medicine, Miami, FL USA; 70 Hirabai Cowasji Jehangir Medical Research Institute, Pune, India; 71 Hypertension Research Center, Cardiovascular Research Center, Isfahan University of Medical Sciences, Isfahan, Iran; 72 ICCAS (Instituto para la Cooperacion Científica en Ambiente y Salud), Buenos Aires, Argentina; 73 ICMR-National Institute of Nutrition, Hyderabad, India; 74 IRCCS Neuromed, Pozzilli, Italy; 75 Institut de Recherche pour le Developpement, Montpellier, France; 76 Institut de Veille Sanitaire, Bobigny, France; 77 Institute for International Investigation, NDRI-USA, New York, NY USA; 78 Institute of Biomedical Sciences, Academia Sinica, Taipei, Taiwan ROC; 79 Institute of Nutrition and Food Technology (INTA), University of Chile, Santiago, Chile; 80 Institute of Nutrition in Central America and Panama (INCAP), Guatemala City, Guatemala; 81 Institute of Public Health of Federation of Bosnia and Herzegovina, Sarajevo, Bosnia and Herzegovina; 82 Institute of Studies in Public Health, Federal University of Rio de Janeiro (UFRJ), Rio de Janeiro, Brazil; 83 Institutes of Biomedical Sciences, Academia Sinica, Taipei, Taiwan ROC; 84 International Agency for Research on Cancer, Lyon, France; 85 International Food Policy Research Institute (IFPRI), Washington, DC USA; 86 Interventional Cardiology Research Center, Cardiovascular Research Center, Isfahan University of Medical Sciences, Isfahan, Iran; 87 Intitut de Recherche en Sciences de la Sante, Bobo-Dioulasso, Burkina Faso; 88 Isfahan Cardiovascular Research Center, Cardiovascular Research Center, Isfahan University of Medical Sciences, Isfahan, Iran; 89 Israel Center for Disease Control, Ramat Gan, Israel; 90 Justus Liebig University Giessen, Giessen, Germany; 91 KLE Academy of Higher Education and Research (Deemed-to-be-University) Jawaharlal Nehru Medical College, Belagavi, India; 92 Kenya Medical Research Institute, Nairobi, Kenya; 93 King Abdulaziz University, Jeddah, Saudi Arabia; 94 King Saud University, Riyadh, Saudi Arabia; 95 King’s College London, London, UK; 96 Kinshasa School of Public Health, Kinshasa, Democratic Republic of Congo; 97 Korea Disease Control and Prevention Agency (KDCA), Cheongju, South Korea; 98 Korea University, Seoul, South Korea; 99 Kuwait Institute for Scientific Research, Kuwait City, Kuwait; 100 Lao Tropical and Public Health Institute, Vientiane, Lao PDR; 101 Lausanne University Hospital (CHUV) and University of Lausanne, Lausanne, Switzerland; 102 Leibniz Centre for Agricultural Landscape Research, Muncheberg, Germany; 103 Loyola University Chicago, Chicago, IL USA; 104 Mahidol University, Nakhon Pathom, Thailand; 105 Mahidol University, Bangkok, Thailand; 106 Malaysian Palm Oil Council (MPOC), Petaling Jaya, Malaysia; 107 Medical Center Markovs, Sofia, Bulgaria; 108 Menopause Andropause Research Center, Ahvaz Jundishapur University of Medical Sciences, Ahvaz, Islamic Republic of Iran; 109 Ministry of Health, Kuala Lumpur, Malaysia; 110 Ministry of Health, Sabak Bernam, Malaysia; 111 Ministry of Health, Victoria, Seychelles; 112 Ministry of Health, Jakarta, Indonesia; 113 NYU School of Medicine, New York, NY USA; 114 National Cancer Center Institute for Cancer Control, Tokyo, Japan; 115 National Centre of Public Health and Analyses (NCPHA), Sofia, Bulgaria; 116 National Food Agency, Uppsala, Sweden; 117 National Food and Nutrition Commission, Lusaka, Zambia; 118 National Food and Nutrition Institute, Warsaw, Poland; 119 National Health Research Institutes, Zhunan township, Taiwan ROC; 120 National Institute for Environmental Studies, Health and Environmental Risk Division, Tsukuba, Japan; 121 National Institute of Nutrition, Hyderabad, India; 122 National Institute of Nutrition, Hanoi, Vietnam; 123 National Institute of Nutrition and Food Technology & SURVEN RL, Tunis, Tunisia; 124 National Institute of Public Health (INSP), Mexico City, Mexico; 125 National Institute of Public Health (INSP), Cuernavaca, Mexico; 126 National Institutes of Biomedical Innovation, Health and Nutrition, Tokyo, Japan; 127 National Nutrition Institute, Cairo, Egypt; 128 National Nutrition and Food Technology Research Institute (NNFTRI): SBMU, Tehran, Islamic Republic of Iran; 129 National University of Malaysia (UKM), Kuala Lumpur, Malaysia; 130 Nestlé Research, Lausanne, Switzerland; 131 New England Complex Systems Institute, Cambridge, MA USA; 132 North-West University, Potchefstroom, South Africa; 133 Oslo Metropolitan University (OsloMet), Oslo, Norway; 134 Perdana University, Puchong, Malaysia; 135 Royal College of Surgeons in Ireland, Dublin, Ireland; 136 Pontificia Universidad Javeriana Seccional, Cali, Colombia; 137 Public Authority for Food and Nutrition, Sabah Al Salem, Kuwait; 138 Public Health Authority of the Slovak Republic, Bratislava, Slovak Republic; 139 Qatar University and University of Jordan, Doha, Qatar; 140 Research Institute for Endocrine Sciences, Shahid Beheshti University of Medical Sciences, Tehran, Islamic Republic of Iran; 141 Research Institute for Primordial Prevention of NCD, Isfahan University of Medical Sciences, Isfahan, Islamic Republic of Iran; 142 Risk and Benefit Assessment Department, Swedish Food Agency, Uppsala, Sweden; 143 Robert Koch Institute, Berlin, Germany; 144 Rutgers University, New Brunswick, NJ USA; 145 Santé publique France, the French Public Health Agency, Saint Maurice, France; 146 Seoul National University, Seoul, South Korea; 147 St John’s Research Institute, Bangalore, India; 148 Tabriz University of Medical Sciences, Tabriz, Islamic Republic of Iran; 149 Tallinn University, Tallinn, Estonia; 150 Taylor’s University, Subang Jaya, Malaysia; 151 Teesside University, Middlesbrough, UK; 152 Tehran University of Medical Sciences, Tehran, Islamic Republic of Iran; 153 Tehran University of Medical Sciences and Utica University, Tehran, Islamic Republic of Iran; 154 The Technical University of Kenya, Nairobi, Kenya; 155 The University of New Mexico, Albuquerque, NM USA; 156 Tropical Diseases Research Centre, Ndola, Zambia; 157 Unidad de Nutricion Publica, Macul, Chile; 158 Department of Biochemistry, University of Puerto Rico – Medical Science Campus, San Juan, Puerto Rico; 159 Universidad San Sebastian, Santiago, Chile; 160 Universidad Tecnica del Norte, Ibarra, Ecuador; 161 Universidad de Antioquia, Medellin, Colombia; 162 Universidad de Cuenca, Cuenca, Ecuador; 163 Universiti Kebangsaan Malaysia Medical Centre, Kuala Lumpur, Malaysia; 164 Universiti Putra Malaysia, Serdang, Malaysia; 165 Universiti Sains Malaysia, Kubang Kerian, Malaysia; 166 University Center for Primary Care and Public Health (Unisanté), Lausanne, Switzerland; 167 University Dunarea de Jos, Galati, Romania; 168 University Kebangsaan Malaysia, Bangi, Malaysia; 169 University of Aberdeen, Aberdeen, UK; 170 University of Alberta, Edmonton, Alberta Canada; 171 University of Bergen, Bergen, Norway; 172 University of Bonn, Department of Nutrition and Food Sciences, Bonn, Germany; 173 University of California Davis, Davis, CA USA; 174 University of Cincinnati, Cincinnati, OH USA; 175 University of Colombo, Colombo, Sri Lanka; 176 University of Colorado School of Medicine, Aurora, CO USA; 177 University of Dhaka, Dhaka, Bangladesh; 178 University of Goettingen, Goettingen, Germany; 179 University of Helsinki, Department of Food and Nutrition, Helsinki, Finland; 180 University of Hohenheim, Stuttgart, Germany; 181 University of Iceland, Reykjavík, Iceland; 182 University of Insubria, Varese, Italy; 183 University of Las Palmas de Gran Canaria (ULPGC), Las Palmas, Spain; 184 University of Malaya, Kuala Lumpur, Malaysia; 185 University of Manchester, Manchester, UK; 186 University of Mauritius, Moka, Mauritius; 187 University of Medicine and Pharmacy Carol Davila, Bucharest, Romania; 188 University of North Carolina at Chapel Hill, Chapel Hill, NC USA; 189 University of Otago, Dunedin, New Zealand; 190 University of Sao Paulo, Sao Paulo, Brazil; 191 University of Saskatchewan, Saskatoon, Saskatchewan Canada; 192 University of Vienna, Vienna, Austria; 193 University of the Southern Caribbean, Port-of-Spain, Trinidad and Tobago; 194 Wageningen University, Wageningen, Netherlands; 195 Washington University in St. Louis, St. Louis, MO USA; 196 World Health Organization (WHO), Geneva, Switzerland; 197 World Health Organization (WHO), Amman, Jordan; 198 Yazd Cardiovascular Research Centre, Shahid Sadoughi University of Medical Sciences, Yazd, Islamic Republic of Iran; 199 Ziauddin University Karachi, Karachi, Pakistan

**Keywords:** Risk factors, Epidemiology

## Abstract

Evidence on what people eat globally is limited in scope and rigour, especially as it relates to children and adolescents. This impairs target setting and investment in evidence-based actions to support healthy sustainable diets. Here we quantified global, regional and national dietary patterns among children and adults, by age group, sex, education and urbanicity, across 185 countries between 1990 and 2018, on the basis of data from the Global Dietary Database project. Our primary measure was the Alternative Healthy Eating Index, a validated score of diet quality; Dietary Approaches to Stop Hypertension and Mediterranean Diet Score patterns were secondarily assessed. Dietary quality is generally modest worldwide. In 2018, the mean global Alternative Healthy Eating Index score was 40.3, ranging from 0 (least healthy) to 100 (most healthy), with regional means ranging from 30.3 in Latin America and the Caribbean to 45.7 in South Asia. Scores among children versus adults were generally similar across regions, except in Central/Eastern Europe and Central Asia, high-income countries, and the Middle East and Northern Africa, where children had lower diet quality. Globally, diet quality scores were higher among women versus men, and more versus less educated individuals. Diet quality increased modestly between 1990 and 2018 globally and in all world regions except in South Asia and Sub-Saharan Africa, where it did not improve.

## Abstract (web summary)

Dietary quality is reported at the global, regional and national level across 185 countries. Though diet quality increased modestly since 1990 at the global level, in South Asia and Sub-Saharan Africa it did not improve. In some regions, children’s dietary quality is lower than that of adults.

## Main

Poor diet is a leading cause of disease worldwide, responsible for an estimated 26% of global preventable mortality^1–4^. While individual foods and nutrients are important, overall dietary patterns are more strongly associated with health^5^. Evidence supports interactive and synergistic relationships between foods and nutrients when consumed together^6^, resulting in complementary effects^5^. While the various components of an optimal dietary pattern are well established and validated^7^, the distributions of such patterns globally are not well characterized. This is particularly true for children and adolescents, among whom global dietary patterns have not previously been reported.

Previous dietary studies have been limited to small subsets of countries^8,9^, used national per capita food availability or sales data as direct data inputs^10–14^, which substantially misestimate intake compared with individual-level data^15^ and did not include children, adolescents or young adults (<25 years old)^8–12,16^. Additionally, there is a paucity of evidence on global disparities in dietary patterns, for example by age, sex, education and urbanicity. Also, no previous global studies have jointly assessed several validated metrics of diet quality^17^, such as the Alternative Healthy Eating Index (AHEI), the Dietary Approaches to Stop Hypertension (DASH) and the Mediterranean Diet Score (MED).

In this Article, to address these gaps in knowledge, we characterized global, regional and national dietary patterns and trends on the basis of individual-level intake data among both adults and children from 185 countries in 1990 and 2018. Findings were further assessed by age, sex, education and urbanicity within each country. This analysis utilized the latest Global Dietary Database (GDD) 2018 data, based on individual-level dietary surveys around the world^18^.

## Results

The GDD is a collaborative effort to systematically identify, compile and standardize individual-level dietary data on 53 foods, beverages and nutrients (Methods). The GDD uses Bayesian modelling methods to estimate dietary intakes jointly stratified by age, sex, education, level and urbanicity for 185 countries between 1990 and 2018.

### Global and regional diet quality in 2018

**Fig. 1:**
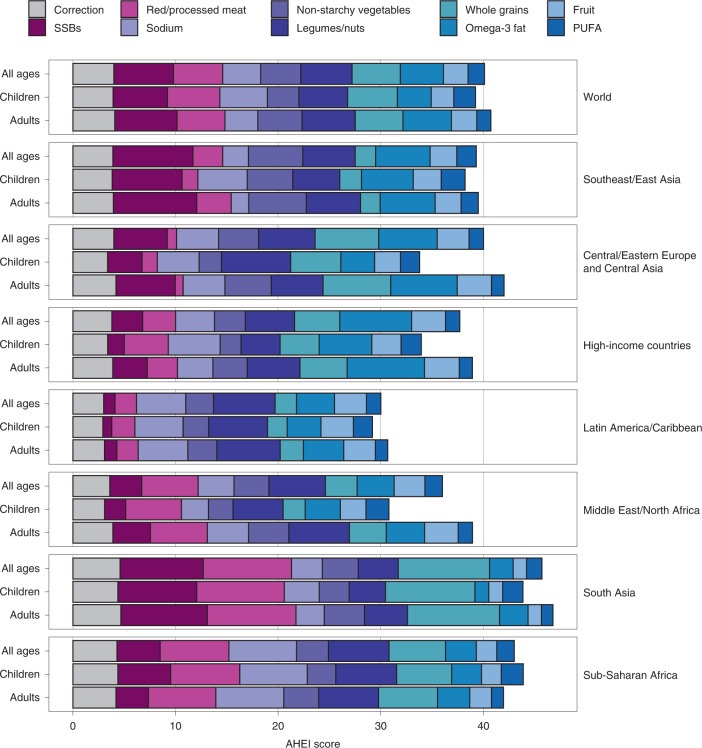
Global and regional mean AHEI component scores by age (all ages, children only and adults only) in 2018. AHEI score: nine components scored from 0 to 10 each and scaled to ten components (correction for *trans* fat shown). Healthy components: fruit, non-starchy vegetables, legumes/nuts, whole grains, PUFAs and seafood omega-3 fat; unhealthy components: red/processed meat, SSBs and sodium.

In 2018, the global mean of the AHEI score was 40.3 (95% uncertainty interval (UI) 39.4, 41.3), with regional means ranging from 30.3 (28.7, 32.2) in Latin America and the Caribbean to 45.7 (43.8, 49.3) in South Asia (Fig. 1). Among components of the score, highest global scores for healthier items were for legumes/nuts (5.0; 4.8, 5.3), followed by whole grains (4.7; 4.5, 5.0), seafood omega-3 fat (4.2; 3.8, 5.1) and non-starchy vegetables (3.9; 3.8, 4.0); among unhealthier items, highest scores (lowest or most favourable intakes) were for sugar-sweetened beverages (SSBs) (5.8; 5.7, 5.9) and red/processed meat (4.8; 4.5, 5.1). However, these score components varied substantially by world region. For example, top scores in South Asia were for higher whole grains and lower red/processed meat and SSBs, while top scores in Latin American and the Caribbean were for higher legumes/nuts and lower sodium.

### National diet quality in 2018

**Fig. 2:**
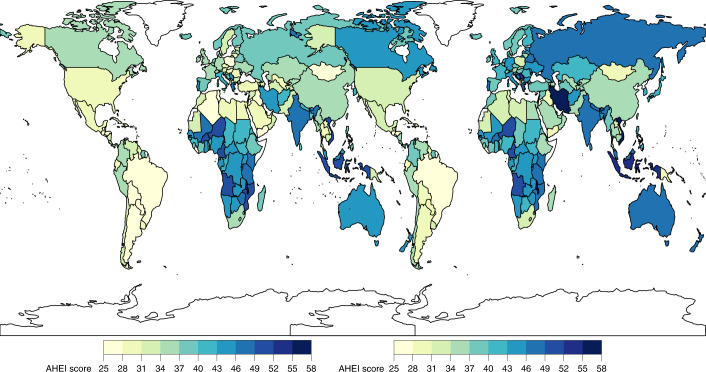
National mean AHEI scores among children (left) and adults (right) in 2018. Children: ≤1 years to ≤19 years; adults: ≥20 years. The AHEI score ranged from 0 to 100. The mean national score was computed as the sum of the stratum-level component scores and aggregated to the national mean using weighted population proportions for 2018.

Only ten countries, representing <1% of the world’s population, had AHEI scores ≥50. Among the world’s 25 most populous countries, the mean AHEI score was highest in Vietnam, Iran, Indonesia and India (54.5 to 48.2) and lowest in Brazil, Mexico, the United States and Egypt (27.1–33.5) (Fig. 2). Most component scores varied substantially across these populous countries. For example, a 100-fold difference was seen in the sodium score, a 90-fold difference in the red/processed meat score and a 23-fold difference in the SSB score. Among the components, the polyunsaturated fatty acid (PUFA) and non-starchy vegetable scores varied the least (two-fold and three-fold, respectively) across populous countries.

### Global and regional differences across demographic subgroups

**Fig. 3:**
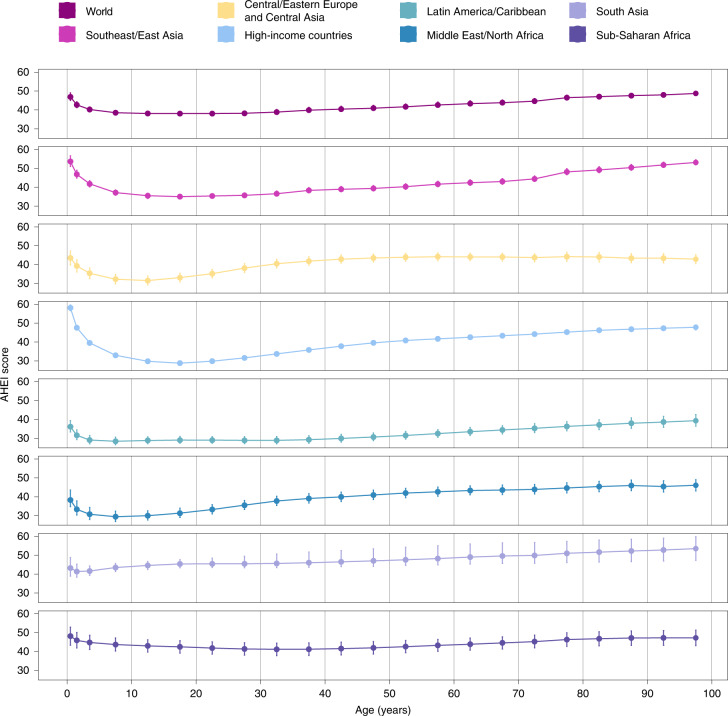
Global and regional mean AHEI scores, by age (years) in 2018. The AHEI score ranged from 0 to 100. The circles represent the global or regional mean for the age group, and the error bars represent the corresponding 95% UI. The mean and its UI are plotted for the midpoint of each age group (<1, 1–2, 3–4, 5–9, 10–14, 15–19, 20–24, 25–29, 30–34, 35–39, 40–44, 45–49, 50–54, 55–59, 60–64, 65–69, 70–74, 75–79, 80–84, 85–89, 90–94 and ≥95 years).

Globally, the mean AHEI score in 2018 was similar among children (39.2; 38.2, 40.3) versus adults (40.8; 39.8, 42.0) (Fig. 1). However, the mean AHEI score was substantially higher among adults compared with children in Central/Eastern Europe and Central Asia, high-income countries, and the Middle East and Northern Africa region. By age, most regions had J- or U-shaped relationships, with the highest scores observed among the youngest (≤5 years) and/or oldest age groups (≥75 years) (Fig. 3).

Among the AHEI components globally, four component scores were lower among children versus adults: fruit (2.2 (2.1, 2.3) versus 2.5 (2.4, 2.5), respectively), non-starchy vegetables (3.1 (3.0, 4.5) versus 4.3 (4.2, 3.2)), SSBs (5.3 (5.1, 5.5) versus 6.1 (6.0, 6.2)) and seafood omega-3 (3.3 (2.9, 4.0) versus 4.7 (4.2, 5.7)), while two others were higher among children versus adults: PUFAs (2.1 (2.0, 2.2) versus 1.4 (1.3, 1.5)) and sodium (4.6 (4.1, 5.1) versus 3.2 (2.9, 3.5)) (Fig. 1).

**Extended Data Fig. 1:**
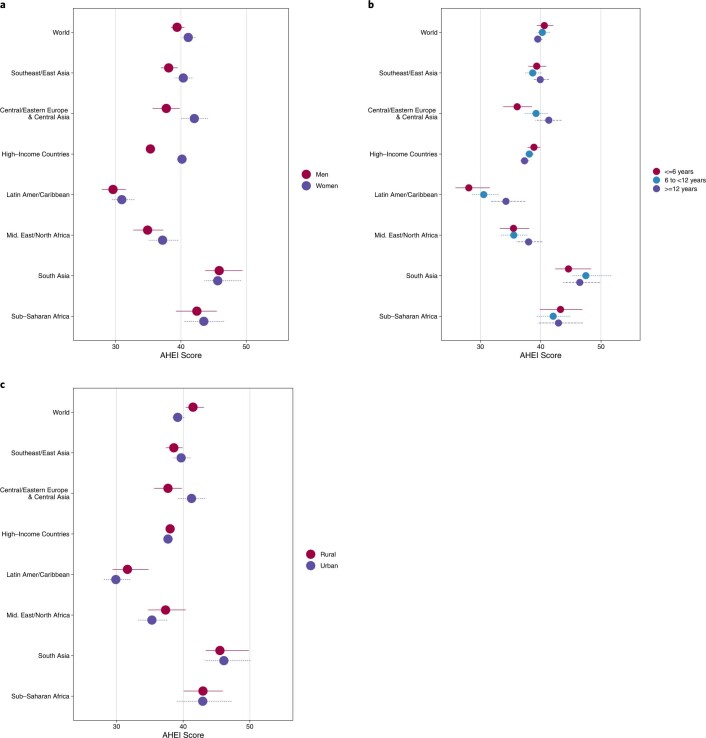
Global and regional AHEI score by (A) sex, (B) education, and (C) urban/rural residence in 2018. Data are the mean score (95% uncertainty interval). The AHEI score ranged from 0 to 100. <6 years: <6 years of education; ≥6 to <12 years: ≥6 to <12 years of education; ≥12 years: ≥12 years of education. Rural: rural residence; urban: urban residence.

By sex, the mean AHEI score was generally higher in women versus men globally and regionally, with the greatest differences seen in high-income countries (difference +4.4; 3.8, 5.0), and Central/Eastern Europe and Central Asia (+3.6; 2.1, 5.3) (Extended Data Fig. 1). Evaluating different AHEI components globally, women had modestly higher scores for fruit (+0.2; 0.2, 0.3), non-starchy vegetables (+0.3; 0.1, 0.4) and whole grains (+0.4; 0.2, 0.5).

**Fig. 4:**
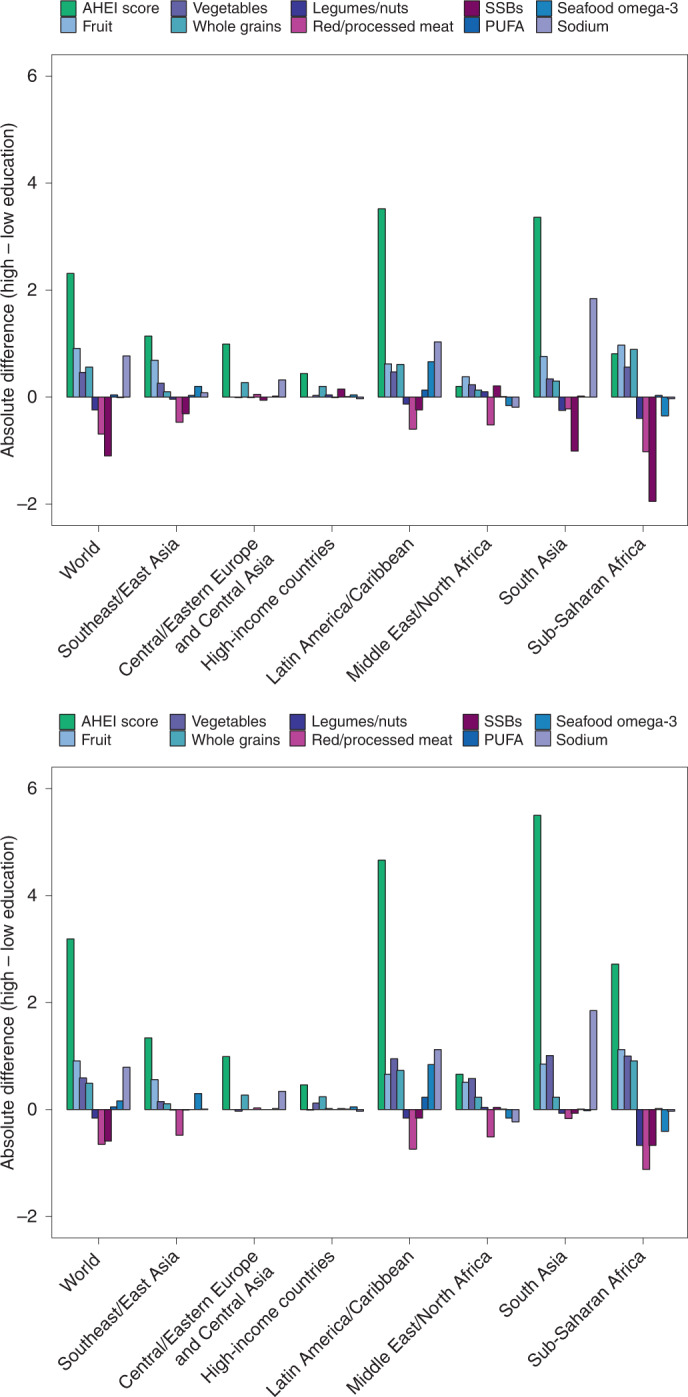
Global and regional mean absolute differences in AHEI component scores in children (top) and adults (bottom) in 2018, by high versus low education level. AHEI score: nine components scored from 0 to 10 each and scaled to ten components (correction not shown). The absolute difference by education was computed as the difference at the stratum level and aggregated to the global and regional mean differences using weighted population proportions for low (<6 years) and high education levels (≥12 years) only (excludes education level ≥6 and <12 years).

Evaluating differences according to educational attainment, AHEI scores were greater among individuals with a higher education level globally and in most regions, except in the Middle East and Northern Africa and Sub-Saharan Africa, where no differences were evident (Fig. 4). Among world regions, differences by education were largest in Central/Eastern Europe and Central Asia (+3.6; 2.4, 4.9), Latin America and the Caribbean (+3.5; 0.9, 6.0) and South Asia (+2.9; 1.1, 4.9). Globally, more educated individuals had higher scores for fruit (+0.8; 0.7, 0.9), sodium (+0.7; 0.3, 1.1), whole grains (+0.6; 0.4, 0.8) and non-starchy vegetables (+0.5; 0.4, 0.6). However, in contrast, more educated individuals also had lower scores (less favourable consumption levels) for red/processed meat (−0.6; −0.7, −0.5), SSBs (−0.6; −0.8, −0.4) and nuts and legumes (−0.1; −0.2, −0.1) globally.

**Fig. 5:**
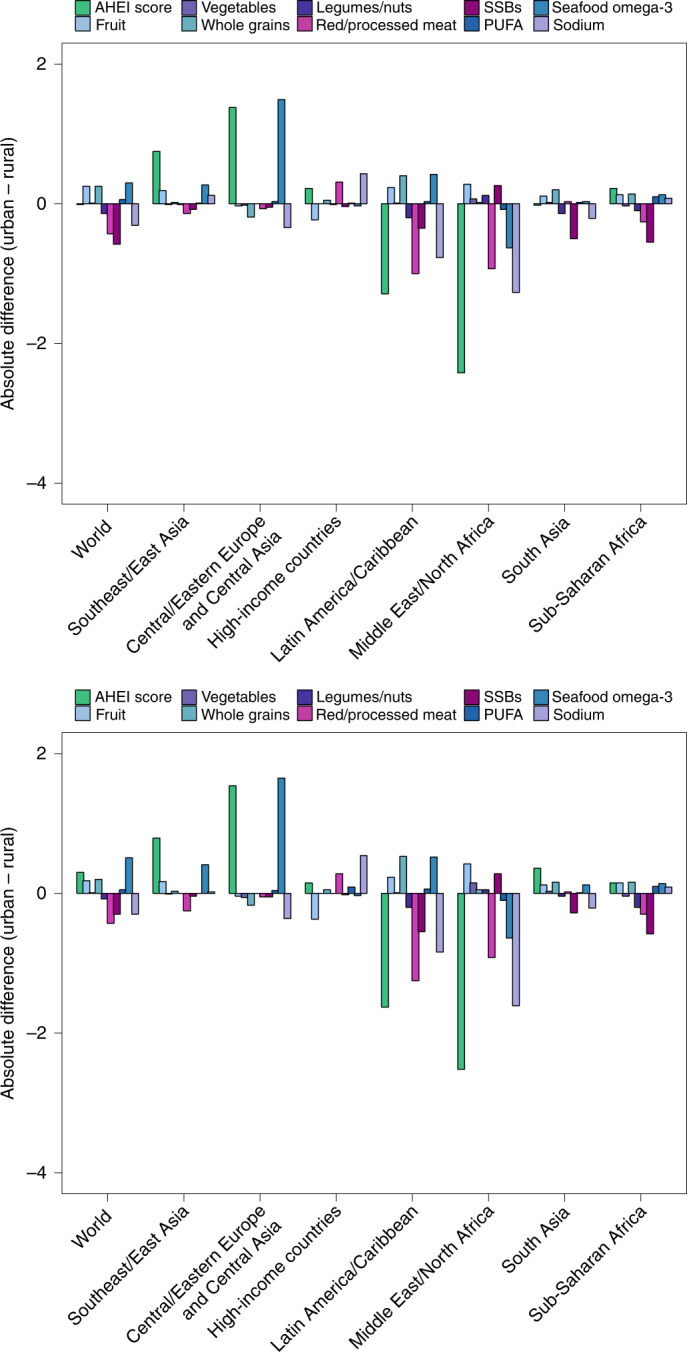
Global and regional mean absolute differences in AHEI component scores in children (top) and adults (bottom) in 2018, by urban versus rural residence. AHEI score: nine components scored from 0 to 10 each and scaled to ten components (correction not shown). The absolute difference by urbanicity was computed as the difference at the stratum level and aggregated to the global and regional mean differences using weighted population proportions.

Globally, AHEI scores did not significantly vary by urban versus rural residence (Fig. 5). However, higher scores were evident among urban versus rural individuals in Central/Eastern Europe and Central Asia (difference +2.2; 0.9, 3.5), and Southeast and East Asia (+1.4; 0.6, 2.4), and lower scores among urban versus rural individuals in the Middle East and Northern Africa (−3.8; −5.5, −2.2). Globally, individuals residing in urban areas had higher scores for fruit (+0.2; 0.2, 0.3) and whole grains (+0.2; 0.1, 0.4), but lower scores for SSBs (−0.5; −0.7, −0.4), red/processed meat (−0.4, −0.5, −0.1) and legumes/nuts (−0.1; −0.2, −0.1).

### Changes in dietary pattern scores between 1990 and 2018

**Fig. 6:**
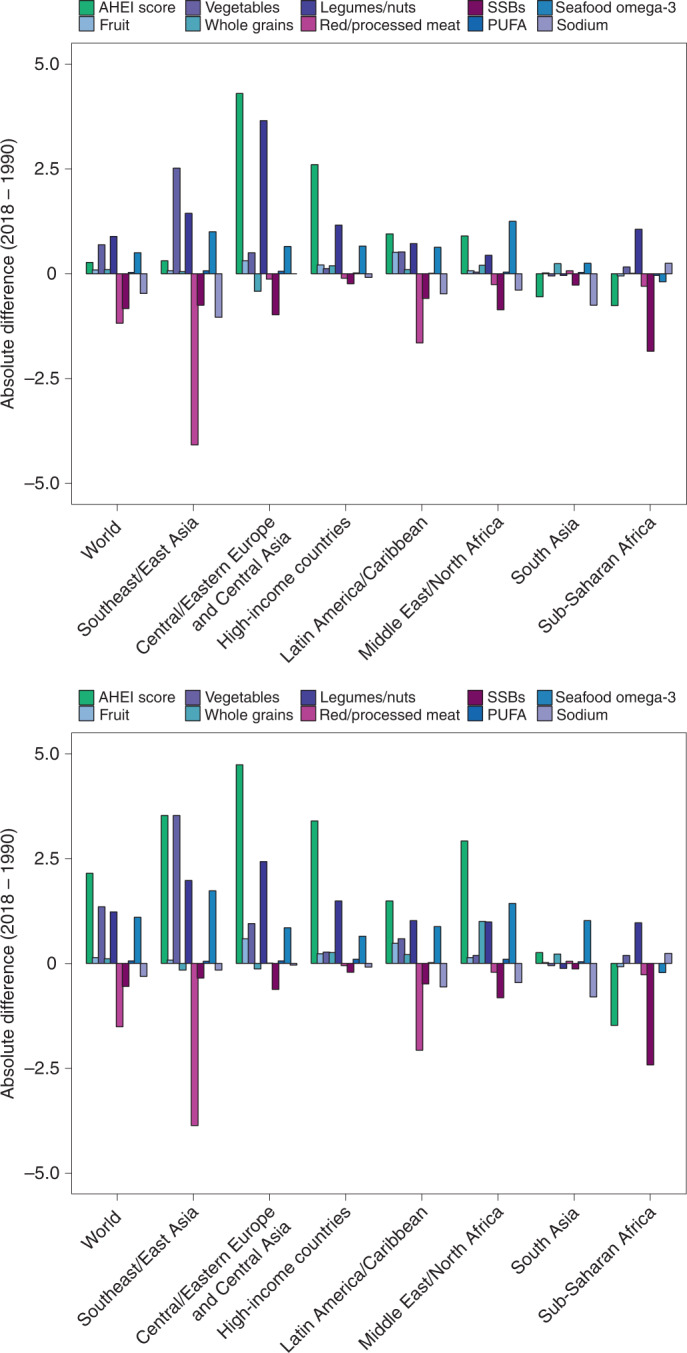
Global and regional mean absolute differences in AHEI component scores in children (top) and adults (bottom), between 2018 and 1990. AHEI score: nine components scored from 0 to 10 each and scaled to ten components (correction not shown). The absolute difference by time was computed as the difference at the stratum level and aggregated to the global and regional mean differences using weighted population proportions for 2018.

Between 1990 and 2018, the mean global AHEI score (standardized to 2018 population distributions) increased by +1.5 (1.0, 2.0). Increasing trends occurred in five of seven regions: Central/Eastern Europe and Central Asia (+4.6; 4.0, 5.3); high-income countries (+3.2; 2.9, 3.5); Southeast and East Asia (+2.7; 1.7, 3.8); the Middle East and Northern Africa (+2.2; 1.4, 3.0); and Latin America and the Caribbean (+1.3; 0.6, 2.0). No significant change was seen in South Asia (0; −0.9, 1.1), and a decreasing trend was seen in Sub-Saharan Africa (−1.1; −1.8, −0.4) (Fig. 6).

Among AHEI components globally, scores increased over time for non-starchy vegetables (+1.1; 1.0, 1.2), legumes/nuts (+1.1; 1.0, 1.3) and fruit (+0.1; 0.1, 0.2); decreased for red/processed meat (−1.4; −1.5, −1.2), SSBs (−0.6; −0.7, −0.6) and sodium (−0.4; −0.6, −0.2); and remained stable for whole grains (+0.1; 0, 0.2), PUFAs (0; 0, 0.1) and seafood omega-3 (0; 0, 0.1).

**Fig. 7:**
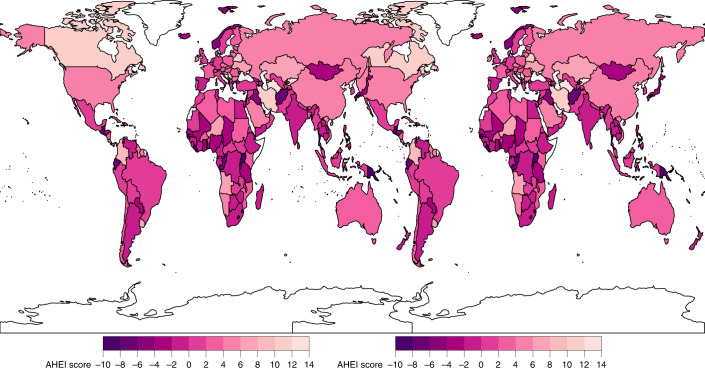
National mean absolute change in AHEI scores among children (left) and adults (right) between 1990 and 2018. The AHEI score ranged from 0 to 100. The absolute difference between 2018 and 1990 was computed as the difference at the stratum level and aggregated to the national mean differences using weighted population proportions for 2018.

Among the most populous countries, the largest absolute improvement in the AHEI score between 1990 and 2018 occurred in Iran (+12.0; 9.9, 13.9), the United States (+4.6; 4.1, 5.1), Vietnam (+4.5; 2.4, 7.2) and China (+4.3; 2.8, 5.9), while the largest declines were found in Tanzania (−3.7; −5.8, −1.5), Nigeria (−3.0; −5.3, −0.7), Japan (−2.7; −3.1, −2.3) and the Philippines (−1.8; −2.7, −0.9) (Fig. 7).

### Results for DASH and MED

**Extended Data Fig. 2:**
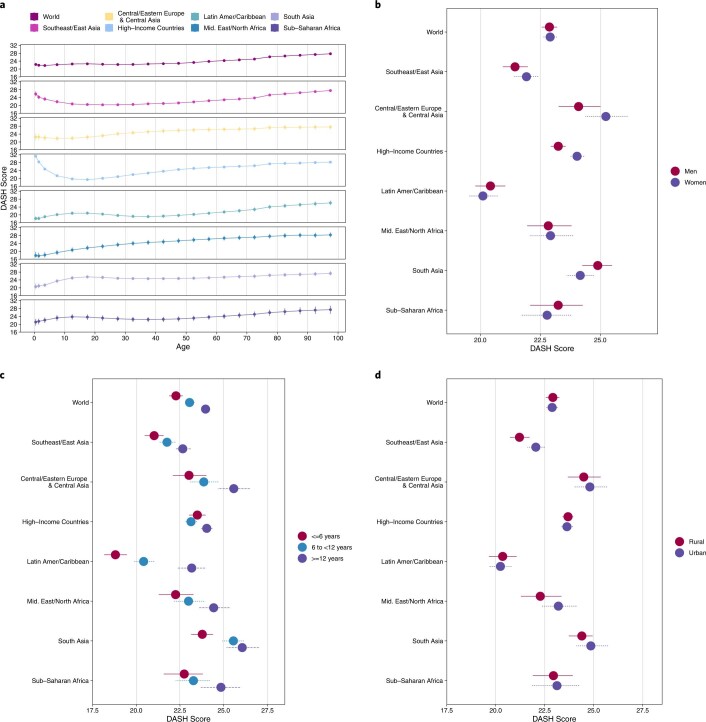
Global and regional DASH score by (A) age, (B) sex, (C) education, and (D) urban/rural residence in 2018. Data are the mean score (95% uncertainty interval). The DASH score ranged from 5 to 40. <6 years: <6 years of education; ≥6 to <12 years: ≥6 to <12 years of education; ≥12 years: ≥12 years of education. Rural: rural residence; urban: urban residence.

**Extended Data Fig. 3:**
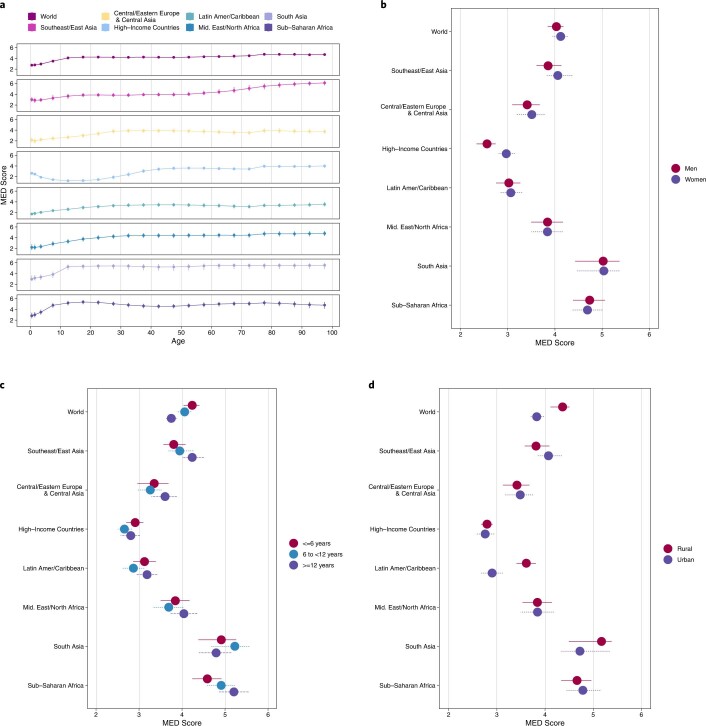
Global and regional MED score by (A) age, (B) sex, (C) education, and (D) urban/rural residence in 2018. Data are the mean score (95% uncertainty interval). The MED score ranged from 0 to 8. <6 years: <6 years of education; ≥6 to <12 years: ≥6 to <12 years of education; ≥12 years: ≥12 years of education. Rural: rural residence; urban: urban residence.

**Extended Data Fig. 4:**
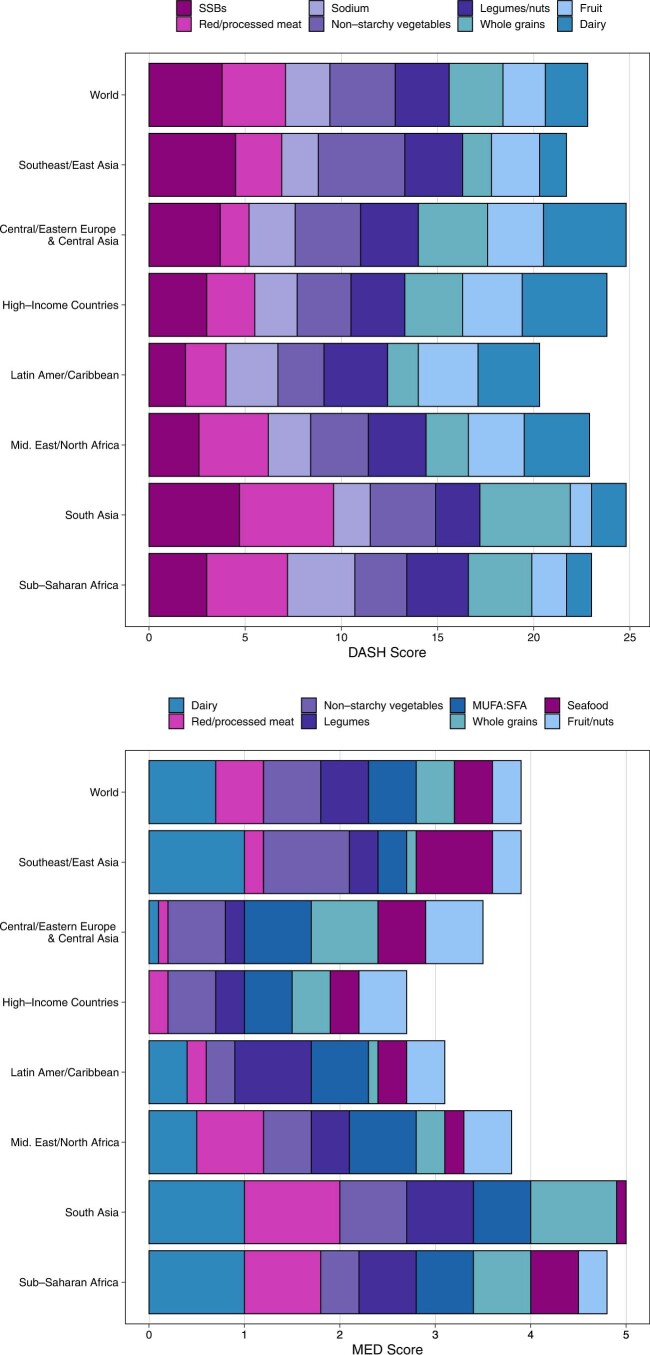
Global and region mean DASH scores (top panel), and MED scores (bottom panel) among all ages (children and adults) in 2018. The DASH score ranged from 5 to 40, and the MED score ranged from 0 to 8.

**Extended Data Fig. 5:**
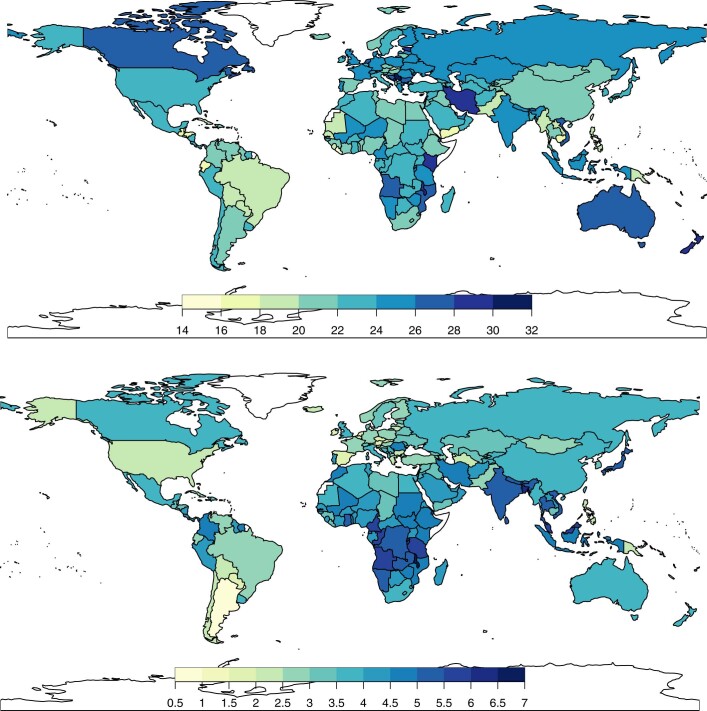
National mean DASH scores (top panel), and MED scores (bottom panel) among all ages (children and adults) in 2018. The DASH score ranged from 5 to 40, and the MED score ranged from 0 to 8.

**Extended Data Fig. 6:**
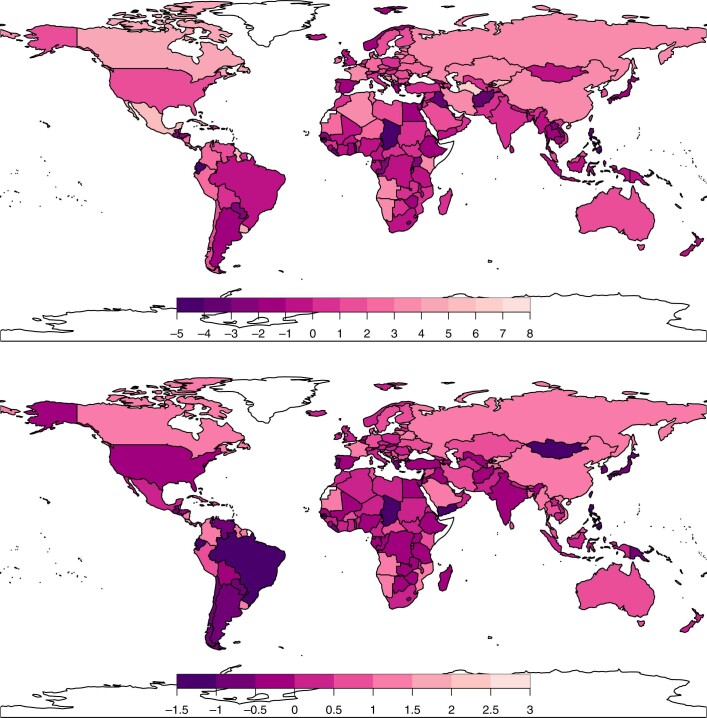
National mean absolute change in DASH scores (top panel), and MED scores (bottom panel) among all ages (children and adults) in 2018. The DASH score ranged from 5 to 40, and the MED score ranged from 0 to 8. The absolute difference between 2018 and 1990 was computed as the difference at the stratum-level and aggregated to the global and regional mean differences using weighted population proportions for 2018.

**Extended Data Fig. 7:**
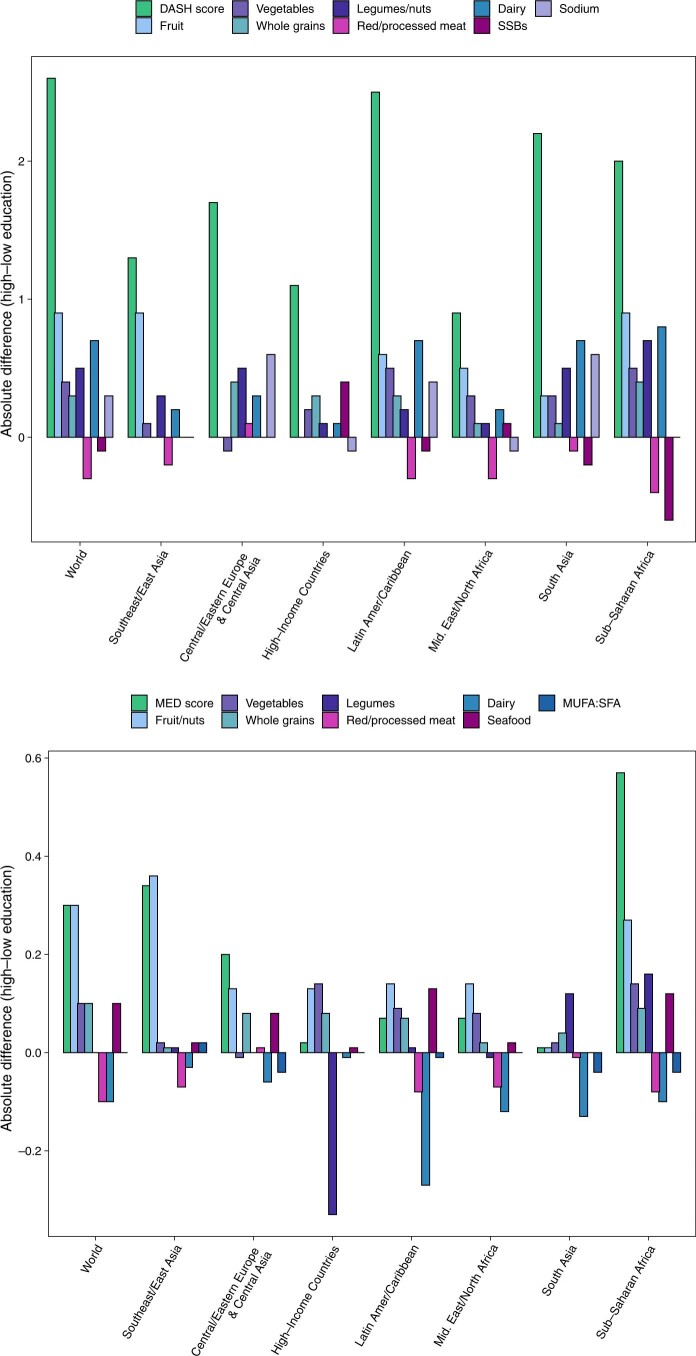
Global and regional mean absolute difference in DASH component scores (top panel) and MED component scores (bottom panel) in all ages (children and adults) in 2018, by high versus low education level. The absolute difference by education was computed as the difference at the stratum-level and aggregated to the global and regional mean differences using weighted population proportions for low (<6 years) and high education levels (≥12 years) only (excludes education level = ≥6 to <12 years).

**Extended Data Fig. 8:**
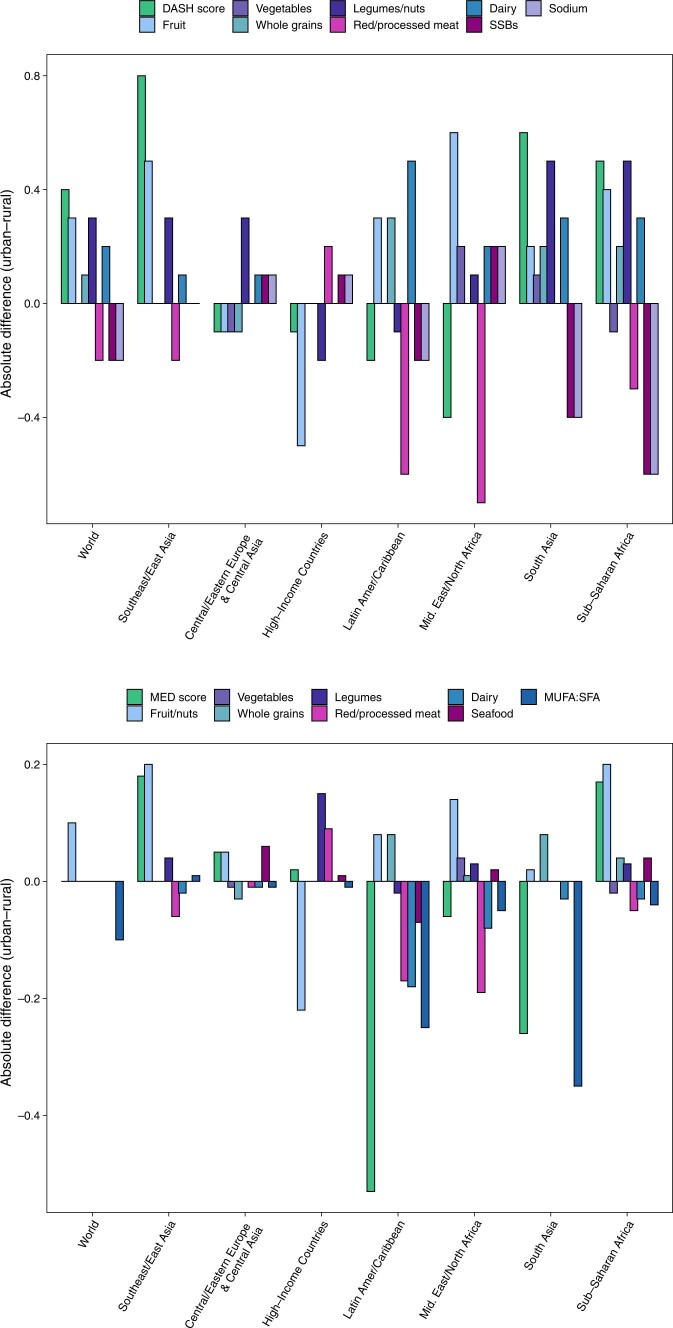
Global and regional mean absolute difference in DASH component scores (top panel) and MED component scores (bottom panel) in all ages (children and adults) in 2018, by urban versus rural residence. The absolute difference by urbanicity was computed as the difference at the stratum-level and aggregated to the global and regional mean differences using weighted population proportions.

**Extended Data Fig. 9:**
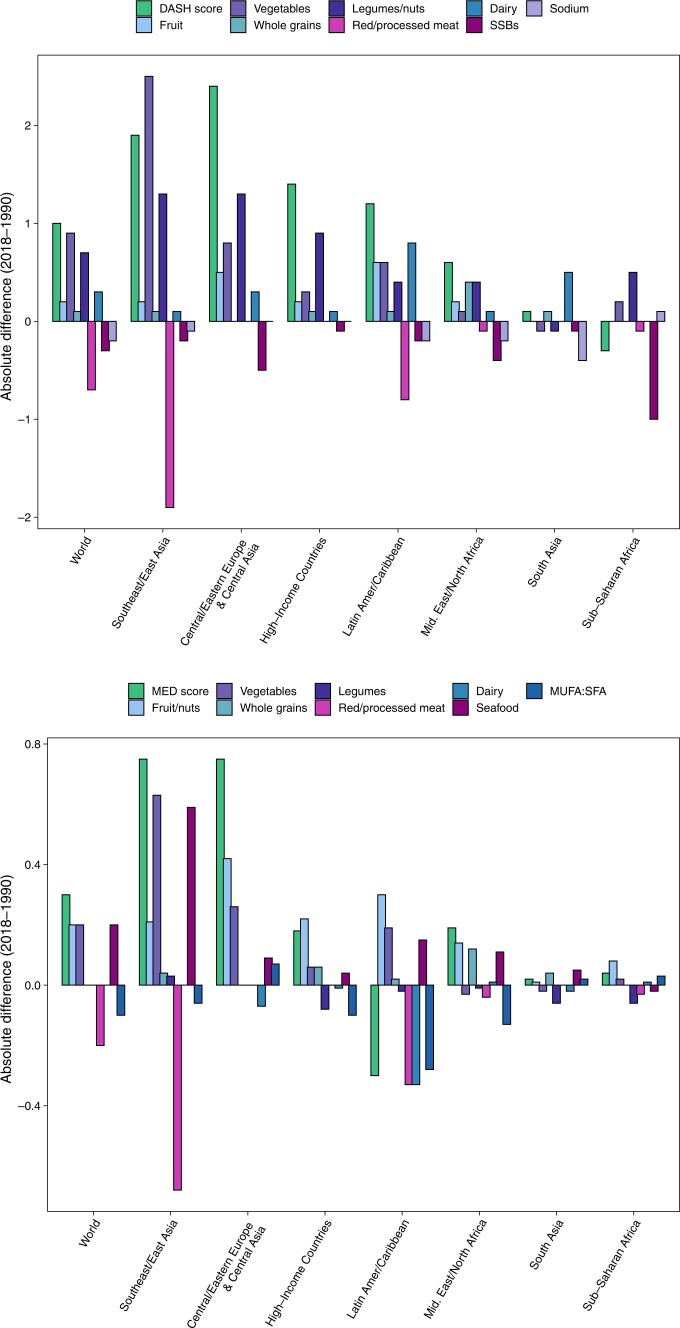
Global and regional mean absolute difference in DASH component scores (top panel) and MED component scores (bottom panel) in all ages (children and adults) between 2018 and 1990. The absolute difference by time was computed as the difference at the stratum-level and aggregated to the global and regional mean differences using weighted population proportions for 2018.

Detailed findings for the DASH and MED scores are presented in Supplementary Information. Briefly, global mean DASH and MED scores in 2018 were 22.9 (22.6, 23.2) and 4.1 (3.9, 4.2), respectively (Extended Data Figs. 2 and 3). Regionally, means for these scores were consistently higher in South Asia, and lower in Latin America and the Caribbean (Extended Data Figs. 4 and 5). Among population subgroups, global DASH and MED scores were higher among adults compared with children (DASH: 23.2 (22.9, 23.4) versus 22.3 (21.9, 22.7); MED: 4.3 (4.1, 4.4) versus 3.7 (3.5, 3.8)), but did not appreciably differ by sex (Extended Data Figs. 2 and 3). Global mean scores were higher among more versus less educated individuals (difference +2.6 (2.3, 2.8) and +0.3 (0.2, 0.4), respectively) (Extended Data Fig. 7), and, for DASH only, among urban versus rural individuals (+0.4; 0.2, 0.7) (Extended Data Fig. 8). Worldwide, the mean DASH and MED scores increased modestly between 1990 and 2018, by +1.0 (0.8, 1.1) for DASH and +0.3 (0.2, 0.4) for MED (Extended Data Figs. 6 and 9). Across strata in 2018, the inter-correlations of the dietary pattern scores were 0.8 for AHEI and DASH, 0.5 for AHEI and MED, and 0.6 for DASH and MED.

## Discussion

In this global assessment of different dietary patterns across 185 countries in 1990 and 2018, we found modest overall dietary quality, but with important variation by age, sex, education, urbanicity, time and world region, as well as by dietary component. These results, based on the systematic collection and standardization of more than 1,100 individual-level dietary surveys worldwide, provide the most current and comprehensive estimates of global, regional and national dietary quality among adults and children, in subgroups according to educational attainment and urban versus rural residence, and comparing three validated dietary patterns including the AHEI, DASH and MED^17^. These results have important implications for public health and inform priorities in each nation and subnational subgroup to improve nutrition security and health equity.

As one example, our findings highlight the regional differences between insufficient intakes of healthful foods versus excess intakes of unhealthful foods. For instance, the highest dietary pattern scores in 2018 were identified in low-income countries in South Asia and Sub-Saharan Africa, where relatively low consumption of SSBs and red/processed meats is consistent with national data on food or beverage volume sales^19^. However, consumption of healthy components, such as fruit, non-starchy vegetables, legumes/nuts, seafood omega-3 fat and PUFAs, were also far from optimal in these nations. This suggests that a major focus on policies and innovations to increase intakes of produce, seafood and plant oils will have the largest impact on dietary quality in these countries.

By contrast, in high-income countries, Central/Eastern Europe and Central Asia, and the Middle East and Northern Africa, increasing intakes of fruit, non-starchy vegetables, legumes/nuts and whole grains have improved dietary quality over time, but have been offset by stable trends or only minor reductions in red/processed meats, SSBs and sodium. We found that red/processed meat and sodium have each significantly increased over time in Asia and Latin America and the Caribbean, consistent with previous nation-specific reports from China, Japan and Mexico^20–22^. These findings suggest that a dual focus on increasing healthful foods and lowering of harmful factors is essential in these regions, especially for nations in Asia and Latin America and the Caribbean.

Several studies have documented that the AHEI is associated with the risk of non-communicable diseases^23^. For example, pooled findings from two US cohorts found a 24%, 33% and 6% reduction in the incidence of cardiovascular disease, diabetes mellitus and cancer, respectively, for the highest AHEI quintile (median 64.5) compared with the lowest quintile (median 36.9; comparable to the global mean in our study, 40.3 (95% UI 39.4, 41.3)) (ref. ^24^). Cohorts have also found that a moderate increase (20-percentile increase) in the AHEI score during follow-up was associated with significantly lower risk of cardiovascular disease mortality and cancer mortality^25^. Similar relationships have been observed in France^26^, the United Kingdom^27^ and Singapore^28,29^. Such associations suggest that the current quality of global diets identified in this study is leading to preventable chronic disease and mortality, and that modest improvements in dietary quality can contribute to reductions in fatal and non-fatal diet-related diseases over time.

Our findings on global diet patterns among infants, children and adolescents have important implications for child nutrition and health. We found that diet quality was generally highest among infants and young children and worsened into adolescence, emphasizing the need for initiatives to aim to improve dietary quality in older children, as well as promote healthy eating habits in early childhood to translate into improved dietary quality in adolescence and adulthood. Although, diet quality was highest among children in Sub-Saharan Africa and South Asia, we found that diet quality worsened or remained stable over time in these regions. Children with more educated parents had higher dietary quality in all regions except South Asia and the Middle East and Northern Africa, while better diet quality was found among children residing in urban areas in Central/Eastern Europe and Central Asia and Southeast and East Asia, and rural areas in the Middle East and Northern Africa. Worse dietary quality in children is associated with stunting, cardiometabolic risk factors (for example, blood pressure, blood lipid levels, glucose control and obesity) and lower health-related quality of life^30–35^, and dietary habits and food preferences established during early life influence later habits throughout childhood and into adulthood^36–38^.

Dietary disparities by education or income level have been reported in specific, mostly high-income nations or selected groups of nations^8,39–41^, but not globally. Our findings demonstrate that more educated individuals had higher overall dietary quality in most, but not all, world regions, with largest impacts of education among nations in Central/Eastern Europe and Central Asia, Latin America and the Caribbean, and South Asia. We also identified key exceptions in the Middle East and Northern Africa, and Sub-Saharan Africa, where dietary quality did not vary by education level. Notably, higher education was generally linked to greater consumption of fruits, non-starchy vegetables, whole grains and plant oils, but not always to lower consumption of SSBs and red/processed meat. Interestingly, urbanicity differentially influenced dietary quality in different world regions, with better dietary quality among urban versus rural residents in Central/Eastern Europe and Central Asia and Southeast and East Asia, but the opposite in the Middle East and Northern Africa, related to specific differences in the consumption of the underlying healthful versus unhealthful components among urban versus rural residents in these regions.

In agreement with our earlier analysis of healthy and unhealthy dietary scores^16^, we found that, compared with lower-income countries, higher-income countries had better scores for healthy components (for example, fruit and whole grains) but worse scores for unhealthy components (for example, red/processed meats and sodium).

This investigation has several strengths. Our data and findings build upon and expand the previous literature by including the largest number of individual-level dietary surveys, providing a more contemporary estimate of trends in global dietary quality and estimating global dietary quality in children and adolescents, which has not been previously reported. We included 1,139 dietary surveys, most of which were nationally representative and collected at the individual-level using 24 h recalls or food-frequency questionnaires (FFQs). We standardized all data inputs including dietary factor definitions, units and age-specific energy adjustment, and incorporated Bayesian modelling with survey and country covariates to address heterogeneity and sampling and modelling uncertainty^42^. We assessed subnational differences by age, sex, education and urbanicity, including the first global estimates of dietary patterns by educational attainment and urban versus rural residence. We characterized three established metrics for diet quality, each validated against major health outcomes^17^, including the similarities and differences in global, regional and national dietary quality depending on the dietary metric.

Potential limitations should be considered. While we made extensive efforts to minimize bias and incorporate heterogeneity and uncertainty, individual-level dietary data are subject to measurement errors, and survey availability was limited or incomplete for some nations, dietary factors, demographic groups and years^16,42^. For example, less than a quarter of surveys included data on children aged 3–9 years and adults ≥85 years. The Bayesian hierarchical models incorporated additional uncertainty to account for these limitations, but sampling and/or information bias cannot be ruled out^16^. To allow for comparability between population subgroups, we standardized dietary intakes to 2,000 kcal per day before computing the dietary patterns, but the unadjusted dietary intakes may be lower among populations with lower energy requirements (for example, infants and young children, and seniors) or higher among populations consuming >2,000 kcal per day. We did not have information on *trans* fat (AHEI) or alcohol use (AHEI and MED), and our findings should be interpreted as dietary quality based on the other components of these scores. The dietary patterns selected (AHEI, MED and DASH) were originally developed and validated for adult populations in high-income countries but have been used to characterize dietary quality among children and seniors^33,43,44^. It is important to note that a single or suite of dietary metrics has not been developed or validated to assess micronutrient quality of the diet in all age groups^17^, and the AHEI, MED and DASH may be inadequately correlated with nutrients of concern, particularly among children and in low- and middle-income countries. Caution is warranted when interpreting the findings in relation to nutrient adequacy. However, in the absence of validated metrics for the double burden of malnutrition, the AHEI, MED and DASH are appropriate metrics for assessing dietary quality across populations^17^. We did not consider other, less validated dietary indices and scores^16,17,45,46^, which can be assessed once these have been better validated for use in diverse global populations.

In conclusion, we found global dietary quality to be only modest today, and with only some improvement, although inconsistent by world region, over the past three decades.

These results provide comprehensive global information about individual-level dietary patterns among children and adults, by age, sex, education and urbanicity. Our findings highlight the substantial variation in dietary quality and inform the need for specific national and subnational policies to improve nutrition security and nutrition equity.

## Methods

### Data sources and retrieval

Our methods and findings for identifying dietary surveys, data extraction, standardization and harmonization, and modelling have been reported^18,42,47,48^. In brief, we systematically searched, identified and collated data from nationally and subnationally representative surveys (or local representative community surveys when national and subnational were not available) on individual-level dietary intakes, and for sodium intake, as well as additional biomarker surveys^18,42^. Household budget surveys were used rarely when individual-level dietary surveys were not identified for a populous country^18,42^. In total, we compiled data from 1,248 dietary surveys from 188 countries. Of these, 1,139 surveys from 175 countries (representing 7.46 billion of the world population in 2018) reported data on the nine foods, three beverages and six nutrients measured in the dietary pattern scores in the present analysis. Most surveys were nationally or subnationally representative (89.1%); used an FFQ (42.1%) or 24 h recall (22.7%); included data on children (0–19 years) (73.9%) and adults (≥20 years) (64.5%); and included data on urban and/or rural residence (60.8%) (Supplementary Information Table 4).

### Data extraction and standardization

For each survey in the GDD, we obtained and assessed the credibility of information obtaining relating to survey characteristics, including survey name, country, years performed, sampling methods, response rate, national representativeness, level of data collection (individual or household), dietary assessment method and validation, sample size, population demographics (age, sex, education, urban/rural residence and pregnancy/lactation status) and definitions and units of dietary factors^18^. We also extracted or obtained directly from the survey owners data on individual-level dietary intakes of up to 53 foods, beverages and nutrients, jointly stratified by age, sex, education and urban/rural residence. We evaluated dietary intakes adjusted to age-standardized energy intakes to assess dietary composition independently of quantity, account for estimated age-specific average requirements and reduce measurement error within and across surveys (Supplementary Information)^42^. Data were assessed for extraction errors and for plausibility using standardized protocols, and survey quality by evaluating evidence for selection bias, sample representativeness, response rate and validity of diet assessment method^42^.

### Modelling and uncertainty

To account for differences in survey methods, representativeness, time trends, input data and uncertainty, a Bayesian model estimated the log-means of dietary intake (mean and standard deviation) within a nested hierarchical structure^42^. The model included random effects by country and region as well as globally; sex, education, urban/rural residence and non-linear age effects; survey-level indicator data for dietary assessment method (24 h recall, FFQ, Demographic Health Survey questionnaire and household budget survey) and type of dietary metric (optimal or suboptimal definition); and national year-specific covariate data relevant to each dietary factor^42^. The model included overdispersion of study-level variance for surveys that were not nationally representative or not stratified by sex, education, urbanicity or small age groups (≤10 years)^42^.

The final model included estimates of consumption of each food or nutrient for 264 subgroups jointly stratified by sex (male or female), age group (<1, 1–2, 3–4, 5–9, 10–14, 15–19, 20–24, 25–29, 30–34, 35–39, 40–44, 45–49, 50–54, 55–59, 60–64, 65–69, 70–74, 75–79, 80–84, 85–89, 90–94 and ≥95 years), education (<6 years, ≥6 to <12 years, or ≥12 years) and urban versus rural residence; within 185 countries covering 99.0% of the world’s population in 2018^42^. Uncertainty of each stratum-specific dietary factor estimate was quantified using 4,000 iterations to determine posterior distributions jointly by country, year, age, sex, education and urbanicity^42^. We computed the median intake and the 95% UI for each stratum from the 50th, 2.5th and 97.5th percentiles of the 4,000 draws, respectively^42^. Validity checks included: five-fold cross-validation (randomly omitting 20% of the raw survey data, run five times), comparing predicted versus observed intakes; assessment of implausible estimates; and visual assessment of national mean intakes using global heat maps^42^. A second time-component-based Bayesian model was used to strengthen time trend estimates for dietary factors with corresponding food or nutrient availability data (FAO Food Balance Sheets^49^ and Global Expanded Nutrient Supply^50^)^42^. The model, commonly referred to as a varying slopes model, incorporated country-level intercepts, and slopes, along with their correlation that is estimated across countries^42,51,52^. The final GDD results were based on these two Bayesian models^42^, as detailed in Supplementary Information.

### Characterization of dietary patterns

For our primary analysis, we focused on the AHEI. For each stratum, we scored nine components: fruit, non-starchy vegetables, whole grains, SSBs, legumes/nuts, unprocessed red/processed meats, seafood omega-3 fat, PUFAs and sodium (alcohol and *trans* fat were not estimated in GDD) (Supplementary Table 6). Each component was scored from 0 to 10, and the final score ranging from 0 to 90 was scaled to range from 0 to 100. DASH was calculated on the basis of eight components, scored from 1 to 5 using sex-specific quintiles, with the final score ranging from 8 to 40 (Supplementary Table 7). MED was calculated on the basis of eight components (alcohol was not estimated), with each component scored as 0 or 1 using sex-specific medians and the final score ranging from 0 to 8 (Supplementary Table 8). As scoring cutpoints for DASH and MED are based on observed population distributions, distributions were calculated for 2018 and used consistently in other years. As each of these scores is based on usual adult intakes, consumption levels of dietary factors in each stratum were standardized to 2,000 kcal per day for deriving the dietary pattern scores. For each dietary pattern, higher scores are given for higher intakes of healthier foods or nutrients and lower intakes of unhealthier foods or nutrients, and thus higher scores represent healthier diets.

### Statistical analysis

Population-weighted average dietary pattern scores for each population subgroup stratum in each country–year were calculated using all 4,000 posterior predictions for each of the components in that stratum to derive global, regional and national scores^42^. Annual population weights were derived from the United Nations Population Division^53^, supplemented with data on educational and urban/rural distributions from Barro Lee^54^ and the United Nations^55^, respectively^42^. Spearman correlations assessed inter-relationships between each dietary pattern score. Changes in scores between 1990 and 2018 were calculated using all 4,000 posterior predictions for each stratum to account for the full spectrum of uncertainty and standardized to the proportion of individuals within each stratum in 2018 to account for changes in demographics over time^42^. Given the Bayesian nature of the analysis, formal statistical significance was not appropriate, and the 95% UIs should be used as a guide^42^.

## Supplementary information

Supplementary Information Supplementary methods, Tables 1–8 and discussion.

## Data Availability

The modelled estimates of individual food and nutrient intakes by population subgroup, country, region and globe in 1990 and 2018 are available for download from the GDD (https://www.globaldietarydatabase.org/). Survey-level information and original data download weblinks are also provided for all public surveys; survey-level microdata or stratum-level aggregate data are provided for direct download for all non-public surveys granted consent for public sharing by the data owner. The modelled dietary quality scores are available for download from (https://github.com/victoriaemiller/GDD-Diet-Quality).

## Code Availability

The statistical coding is available from the corresponding author on reasonable request.

## References

[1] *2021 Global Nutrition Report: The State of Global Nutrition* (Development Initiatives, 2021).

[2] GBD 2017 Causes of Death Collaborators. Global, regional, and national age-sex-specific mortality for 282 causes of death in 195 countries and territories, 1980–2017: a systematic analysis for the Global Burden of Disease Study 2017. *Lancet*10.1016/s0140-6736(18)32203-7 (2018). 10.1016/S0140-6736(18)32203-7 PMC6227606 30496103

[3] GBD 2017 Risk Factor Collaborators. Global, regional, and national comparative risk assessment of 84 behavioural, environmental and occupational, and metabolic risks or clusters of risks for 195 countries and territories, 1990–2017: a systematic analysis for the Global Burden of Disease Study 2017. *Lancet*10.1016/s0140-6736(18)32225-6 (2018). 10.1016/S0140-6736(18)32225-6 PMC6227755 30496105

[4] GBD 2017 Diet Collaborators. Health effects of dietary risks in 195 countries, 1990–2017: a systematic analysis for the Global Burden of Disease Study 2017. *Lancet*10.1016/s0140-6736(19)30041-8 (2019). 10.1016/S0140-6736(19)30041-8 PMC6899507 30954305

[5] Sacks FM (1995). Rationale and design of the Dietary Approaches to Stop Hypertension trial (DASH). A multicenter controlled-feeding study of dietary patterns to lower blood pressure. Ann. Epidemiol..

[6] Hu FB (2002). Dietary pattern analysis: a new direction in nutritional epidemiology. Curr. Opin. Lipidol..

[7] Micha, R. et al. Etiologic effects and optimal intakes of foods and nutrients for risk of cardiovascular diseases and diabetes: systematic reviews and meta-analyses from the Nutrition and Chronic Diseases Expert Group (NutriCoDE). *PLoS ONE* **12**, e0175149 (2017). 10.1371/journal.pone.0175149 PMC5407851 28448503

[8] Teo K (2013). Prevalence of a healthy lifestyle among individuals with cardiovascular disease in high-, middle- and low-income countries: the Prospective Urban Rural Epidemiology (PURE) study. JAMA.

[9] Yusuf S (2020). Modifiable risk factors, cardiovascular disease, and mortality in 155 722 individuals from 21 high-income, middle-income, and low-income countries (PURE): a prospective cohort study. Lancet.

[10] Ezzati M, Riboli E (2013). Behavioral and dietary risk factors for noncommunicable diseases. N. Engl. J. Med..

[11] Kennedy G, Nantel G, Shetty P (2004). Globalization of food systems in developing countries: impact on food security and nutrition. FAO Food Nutr. Pap..

[12] Wang DD (2019). Global improvement in dietary quality could lead to substantial reduction in premature death. J. Nutr..

[13] Vandevijvere S (2013). Monitoring and benchmarking population diet quality globally: a step-wise approach. Obes. Rev..

[14] Green R, Sutherland J, Dangour AD, Shankar B, Webb P (2016). Global dietary quality, undernutrition and non-communicable disease: a longitudinal modelling study. BMJ Open.

[15] Del Gobbo LC (2015). Assessing global dietary habits: a comparison of national estimates from the FAO and the Global Dietary Database. Am. J. Clin. Nutr..

[16] Imamura F (2015). Dietary quality among men and women in 187 countries in 1990 and 2010: a systematic assessment. Lancet Glob. Health.

[17] Miller V, Webb P, Micha R, Mozaffarian D (2020). Defining diet quality: a synthesis of dietary quality metrics and their validity for the double burden of malnutrition. Lancet Planet Health.

[18] Miller V (2021). Global Dietary Database 2017: data availability and gaps on 54 major foods, beverages and nutrients among 5.6 million children and adults from 1220 surveys worldwide. BMJ Global Health.

[19] Vandevijvere S (2019). Global trends in ultraprocessed food and drink product sales and their association with adult body mass index trajectories. Obes. Rev..

[20] Li Y (2017). Time trends of dietary and lifestyle factors and their potential impact on diabetes burden in China. Diabetes Care.

[21] Murakami K, Livingstone MBE, Sasaki S (2018). Thirteen-year trends in dietary patterns among Japanese adults in the National Health and Nutrition Survey 2003–2015: continuous Westernization of the Japanese diet. Nutrients.

[22] Marrón-Ponce JA, Tolentino-Mayo L, Hernández-F M, Batis C (2018). Trends in ultra-processed food purchases from 1984 to 2016 in Mexican Households. Nutrients.

[23] Schwingshackl L, Bogensberger B, Hoffmann G (2018). Diet quality as assessed by the healthy eating index, alternate healthy eating index, dietary approaches to stop hypertension score, and health outcomes: an updated systematic review and meta-analysis of cohort studies. J. Acad. Nutr. Diet.

[24] Chiuve SE (2012). Alternative dietary indices both strongly predict risk of chronic disease. J. Nutr..

[25] Sotos-Prieto M (2017). Association of changes in diet quality with total and cause-specific mortality. N. Engl. J. Med..

[26] Trébuchet A (2019). Prospective association between several dietary scores and risk of cardiovascular diseases: is the Mediterranean diet equally associated to cardiovascular diseases compared to National Nutritional Scores?. Am. Heart J..

[27] Shivappa N, Hebert JR, Kivimaki M, Akbaraly T (2017). Alternative Healthy Eating Index 2010, Dietary Inflammatory Index and risk of mortality: results from the Whitehall II cohort study and meta-analysis of previous Dietary Inflammatory Index and mortality studies. Br. J. Nutr..

[28] Neelakantan N, Koh W-P, Yuan J-M, van Dam RM (2018). Diet-quality indexes are associated with a lower risk of cardiovascular, respiratory, and all-cause mortality among Chinese adults. J. Nutr..

[29] Chen G-C (2018). Diet quality indices and risk of type 2 diabetes mellitus: The Singapore Chinese Health Study. Am. J. Epidemiol..

[30] Wu XY (2019). The influence of diet quality and dietary behavior on health-related quality of life in the general population of children and adolescents: a systematic review and meta-analysis. Qual. Life Res..

[31] Dalwood P, Marshall S, Burrows TL, McIntosh A, Collins CE (2020). Diet quality indices and their associations with health-related outcomes in children and adolescents: an updated systematic review. Nutr. J..

[32] Jennings A, Welch A, van Sluijs EM, Griffin SJ, Cassidy A (2011). Diet quality is independently associated with weight status in children aged 9-10 years. J. Nutr..

[33] Marshall S, Burrows T, Collins CE (2014). Systematic review of diet quality indices and their associations with health-related outcomes in children and adolescents. J. Hum. Nutr. Diet.

[34] Krasevec, J., An, X., Kumapley, R., Bégin, F. & Frongillo, E. A. Diet quality and risk of stunting among infants and young children in low- and middle-income countries. *Matern. Child Nutr.*10.1111/mcn.12430 (2017). 10.1111/mcn.12430 PMC6865990 29032628

[35] Martin-Calvo N, Chavarro JE, Falbe J, Hu FB, Field AE (2016). Adherence to the Mediterranean dietary pattern and BMI change among US adolescents. Int. J. Obes..

[36] Switkowski KM, Gingras V, Rifas-Shiman SL, Oken E (2020). Patterns of complementary feeding behaviors predict diet quality in early childhood. Nutrients.

[37] Mikkilä V, Räsänen L, Raitakari OT, Pietinen P, Viikari J (2004). Longitudinal changes in diet from childhood into adulthood with respect to risk of cardiovascular diseases: The Cardiovascular Risk in Young Finns Study. Eur. J. Clin. Nutr..

[38] Lake AA, Mathers JC, Rugg-Gunn AJ, Adamson AJ (2006). Longitudinal change in food habits between adolescence (11–12 years) and adulthood (32–33 years): the ASH30 Study. J. Public Health.

[39] Wang DD (2014). Trends in dietary quality among adults in the United States, 1999 through 2010. JAMA Intern. Med..

[40] Fang Zhang F (2018). Trends and disparities in diet quality among US adults by supplemental nutrition assistance program participation status. JAMA Netw. Open.

[41] Dehghan M (2012). Relationship between healthy diet and risk of cardiovascular disease among patients on drug therapies for secondary prevention. Circulation.

[42] Miller V (2022). Global, regional, and national consumption of animal-source foods between 1990 and 2018: findings from the Global Dietary Database. Lancet Planet. Health.

[43] Chen X, Maguire B, Brodaty H, O’Leary F (2019). Dietary patterns and cognitive health in older adults: a systematic review. J. Alzheimers Dis..

[44] Roman B, Carta L, Martínez-González MA, Serra-Majem L (2008). Effectiveness of the Mediterranean diet in the elderly. Clin. Interv. Aging.

[45] Bromage S (2021). Development and validation of a novel food-based global diet quality score (GDQS). J. Nutr..

[46] Herforth AW, Wiesmann D, Martínez-Steele E, Andrade G, Monteiro CA (2020). Introducing a suite of low-burden diet quality indicators that reflect healthy diet patterns at population level. Curr. Dev. Nutr..

[47] Micha R (2012). Estimating the global and regional burden of suboptimal nutrition on chronic disease: methods and inputs to the analysis. Eur. J. Clin. Nutr..

[48] Khatibzadeh S (2016). A global database of food and nutrient consumption. Bull. World Health Organ..

[49] Food and Agriculture Organization of the United Nations. Food balances. 2021. https://www.fao.org/faostat/en/#data2021 (accessed March 3, 2021).

[50] Smith MR, Micha R, Golden CD, Mozaffarian D, Myers SS (2016). Global Expanded Nutrient Supply (GENuS) Model: a new method for estimating the global dietary supply of nutrients. PLoS ONE.

[51] Gelman A, Pardoe I (2006). Bayesian measures of explained variance and pooling in multilevel (hierarchical) models. Technometrics.

[52] Wagner T, Diefenbach DR, Christensen S, Norton AS (2011). Using multilevel models to quantify heterogeneity in resource selection. J. Wildl. Manag..

[53] United Nations Population Division. Total population by sex (thousands). 2019. https://population.un.org/wpp/DataQuery/2019 (accessed June 12, 2020).

[54] Barro R, Lee J (2013). A new data set of educational attainment in the world, 1950–2010. J. Dev. Econ.

[55] United Nations Population Division. Urban population (% of total population). 2018. https://data.worldbank.org/indicator/SP.URB. TOTL.IN.ZS2018 (accessed June 12, 2020).

