# Author Correction: Global dietary quality in 185 countries from 1990 to 2018 show wide differences by nation, age, education, and urbanicity

**DOI:** 10.1038/s43016-023-00705-0

**Published:** 2023-01-24

**Authors:** Victoria Miller, Patrick Webb, Frederick Cudhea, Peilin Shi, Jianyi Zhang, Julia Reedy, Josh Erndt-Marino, Jennifer Coates, Dariush Mozaffarian, Global Dietary Database, Murat Bas, Jemal Haidar Ali, Suhad Abumweis, Anand Krishnan, Puneet Misra, Nahla Chawkat Hwalla, Chandrashekar Janakiram, Nur Indrawaty Liputo, Abdulrahman Musaiger, Farhad Pourfarzi, Iftikhar Alam, Karin DeRidder, Celine Termote, Anjum Memon, Aida Turrini, Elisabetta Lupotto, Raffaela Piccinelli, Stefania Sette, Karim Anzid, Marieke Vossenaar, Paramita Mazumdar, Ingrid Rached, Alicia Rovirosa, María Elisa Zapata, Tamene Taye Asayehu, Francis Oduor, Julia Boedecker, Lilian Aluso, Johana Ortiz-Ulloa, J. V. Meenakshi, Michelle Castro, Giuseppe Grosso, Anna Waskiewicz, Umber S. Khan, Anastasia Thanopoulou, Reza Malekzadeh, Neville Calleja, Marga Ocke, Zohreh Etemad, Mohannad Al Nsour, Lydiah M. Waswa, Eha Nurk, Joanne Arsenault, Patricio Lopez-Jaramillo, Abla Mehio Sibai, Albertino Damasceno, Carukshi Arambepola, Carla Lopes, Milton Severo, Nuno Lunet, Duarte Torres, Heli Tapanainen, Jaana Lindstrom, Suvi Virtanen, Cristina Palacios, Eva Roos, Imelda Angeles Agdeppa, Josie Desnacido, Mario Capanzana, Anoop Misra, Ilse Khouw, Swee Ai Ng, Edna Gamboa Delgado, Mauricio Caballero, Johanna Otero, Hae-Jeung Lee, Eda Koksal, Idris Guessous, Carl Lachat, Stefaan De Henauw, Ali Reza Rahbar, Alison Tedstone, Androniki Naska, Angie Mathee, Annie Ling, Bemnet Tedla, Beth Hopping, Brahmam Ginnela, Catherine Leclercq, Charmaine Duante, Christian Haerpfer, Christine Hotz, Christos Pitsavos, Colin Rehm, Coline van Oosterhout, Corazon Cerdena, Debbie Bradshaw, Dimitrios Trichopoulos, Dorothy Gauci, Dulitha Fernando, Elzbieta Sygnowska, Erkki Vartiainen, Farshad Farzadfar, Gabor Zajkas, Gillian Swan, Guansheng Ma, Gulden Pekcan, Hajah Masni Ibrahim, Harri Sinkko, Helene Enghardt Barbieri, Isabelle Sioen, Jannicke Myhre, Jean-Michel Gaspoz, Jillian Odenkirk, Kanitta Bundhamcharoen, Keiu Nelis, Khairul Zarina, Lajos Biro, Lars Johansson, Laufey Steingrimsdottir, Leanne Riley, Mabel Yap, Manami Inoue, Maria Szabo, Marja-Leena Ovaskainen, Meei-Shyuan Lee, Mei Fen Chan, Melanie Cowan, Mirnalini Kandiah, Ola Kally, Olof Jonsdottir, Pam Palmer, Peter Vollenweider, Philippos Orfanos, Renzo Asciak, Robert Templeton, Rokiah Don, Roseyati Yaakub, Rusidah Selamat, Safiah Yusof, Sameer Al-Zenki, Shu-Yi Hung, Sigrid Beer-Borst, Suh Wu, Widjaja Lukito, Wilbur Hadden, Wulf Becker, Xia Cao, Yi Ma, Yuen Lai, Zaiton Hjdaud, Didier Garriguet, Jennifer Ali, Ron Gravel, Tina Tao, Jacob Lennert Veerman, Shashi Chiplonkar, Mustafa Arici, Le Tran Ngoan, Demosthenes Panagiotakos, Yanping Li, Antonia Trichopoulou, Noel Barengo, Anuradha Khadilkar, Veena Ekbote, Noushin Mohammadifard, Irina Kovalskys, Avula Laxmaiah, Harikumar Rachakulla, Hemalatha Rajkumar, Indrapal Meshram, Laxmaiah Avula, Nimmathota Arlappa, Rajkumar Hemalatha, Licia lacoviello, Marialaura Bonaccio, Simona Costanzo, Yves Martin-Prevel, Katia Castetbon, Nattinee Jitnarin, Yao-Te Hsieh, Sonia Olivares, Gabriela Tejeda, Aida Hadziomeragic, Amanda de Moura Souza, Wen-Harn Pan, Inge Huybrechts, Alan de Brauw, Mourad Moursi, Maryam Maghroun, Augustin Nawidimbasba Zeba, Nizal Sarrafzadegan, Lital Keinan-Boker, Rebecca Goldsmith, Tal Shimony, Irmgard Jordan, Shivanand C. Mastiholi, Moses Mwangi, Yeri Kombe, Zipporah Bukania, Eman Alissa, Nasser Al-Daghri, Shaun Sabico, Martin Gulliford, Tshilenge S. Diba, Kyungwon Oh, Sanghui Kweon, Sihyun Park, Yoonsu Cho, Suad Al-Hooti, Chanthaly Luangphaxay, Daovieng Douangvichit, Latsamy Siengsounthone, Pedro Marques-Vidal, Constance Rybak, Amy Luke, Nipa Rojroongwasinkul, Noppawan Piaseu, Kalyana Sundram, Donka Baykova, Parvin Abedi, Fariza Fadzil, Noriklil Bukhary Ismail Bukhary, Pascal Bovet, Sandjaja Sandjaja, Yu Chen, Norie Sawada, Shoichiro Tsugane, Lalka Rangelova, Stefka Petrova, Vesselka Duleva, Anna Karin Lindroos, Jessica Petrelius Sipinen, Lotta Moraeus, Per Bergman, Ward Siamusantu, Lucjan Szponar, Hsing-Yi Chang, Makiko Sekiyama, Balakrishna Nagalla, Kalpagam Polasa, Sesikeran Boindala, Khanh Le Nguyen Bao, Jalila El Ati, Daniel Illescas-Zarate, Luz Maria Sanchez-Romero, Ivonne Ramirez Silva, Juan Rivera Dommarco, Simon Barquera, Sonia Rodríguez-Ramírez, Nayu Ikeda, Sahar Zaghloul, Anahita Houshiar-rad, Fatemeh Mohammadi-Nasrabadi, Morteza Abdollahi, Khun-Aik Chuah, Zaleha Abdullah Mahdy, Alison Eldridge, Eric L. Ding, Herculina Kruger, Sigrun Henjum, Anne Fernandez, Milton Fabian Suarez-Ortegon, Nawal Al-Hamad, Veronika Janská, Reema Tayyem, Parvin Mirmiran, Roya Kelishadi, Eva Warensjo Lemming, Almut Richter, Gert Mensink, Lothar Wieler, Daniel Hoffman, Benoit Salanave, Cho-il Kim, Rebecca Kuriyan-Raj, Sumathi Swaminathan, Saeed Dastgiri, Sirje Vaask, Tilakavati Karupaiah, Fatemeh Vida Zohoori, Alireza Esteghamati, Sina Noshad, Maryam Hashemian, Elizabeth Mwaniki, Elizabeth Yakes-Jimenez, Justin Chileshe, Sydney Mwanza, Lydia Lera Marques, Alan Martin Preston, Samuel Duran Aguero, Mariana Oleas, Luz Posada, Angelica Ochoa, Khadijah Shamsuddin, Zalilah Mohd Shariff, Hamid Jan Bin Jan Mohamed, Wan Manan, Anca Nicolau, Cornelia Tudorie, Bee Koon Poh, Pamela Abbott, Mohammadreza Pakseresht, Sangita Sharma, Tor Strand, Ute Alexy, Ute Nöthlings, Jan Carmikle, Ken Brown, Jeremy Koster, Indu Waidyatilaka, Pulani Lanerolle, Ranil Jayawardena, Julie M. Long, K. Michael Hambidge, Nancy F. Krebs, Aminul Haque, Gudrun B. Keding, Liisa Korkalo, Maijaliisa Erkkola, Riitta Freese, Laila Eleraky, Wolfgang Stuetz, Inga Thorsdottir, Ingibjorg Gunnarsdottir, Lluis Serra-Majem, Foong Ming Moy, Simon Anderson, Rajesh Jeewon, Corina Aurelia Zugravu, Linda Adair, Shu Wen Ng, Sheila Skeaff, Dirce Marchioni, Regina Fisberg, Carol Henry, Getahun Ersino, Gordon Zello, Alexa Meyer, Ibrahim Elmadfa, Claudette Mitchell, David Balfour, Johanna M. Geleijnse, Mark Manary, Laetitia Nikiema, Tatyana El-kour, Masoud Mirzaei, Rubina Hakeem

**Affiliations:** 1 Friedman School of Nutrition Science and Policy, Tufts University, Boston, MA USA; 2 Department of Medicine, McMaster University, Hamilton, Ontario Canada; 3 Population Health Research Institute, Hamilton, Ontario Canada; 4 Division of General Internal Medicine and Primary Care, Brigham and Women’s Hospital, Boston, MA USA; 5 Acibadem University, Istanbul, Turkey; 6 Addis Ababa University, Addis Ababa, Ethiopia; 7 Al Ain University, Abu Dhabi, UAE; 8 All India Institute of Medical Sciences, New Delhi, India; 9 American University of Beirut, Beirut, Lebanon; 10 Amrita School of Dentistry, Ernakulam, India; 11 Andalas University, Padang, Indonesia; 12 Arab Center for Nutrition, Manama, Bahrain; 13 Ardabil University of Medical Sciences, Ardabil, Islamic Republic of Iran; 14 Bacha Khan University, Charsadda, Pakistan; 15 Belgian Public Health Institute, Brussels, Belgium; 16 Biodiversity International, Maccarese, Italy; 17 Brighton and Sussex Medical School, Brighton, UK; 18 CREA-Alimenti e Nutrizione, Rome, Italy; 19 Cadi Ayyad University, Ben Guerir, Morocco; 20 Center for Studies of Sensory Impairment, Aging and Metabolism (CeSSIAM), Guatemala City, Guatemala; 21 Centre For Media Studies, New Delhi, India; 22 Centro de Atencion Nutricional Antimano (CANIA), Miami, FL USA; 23 Centro de Estudios sobre Nutrición Infantil (CESNI), Buenos Aires, Argentina; 24 College of Applied Sciences, Department of Food Science and Applied Nutrition, Addis Ababa Science and Technology University, Addis Ababa, Ethiopia; 25 Consultative Group on International Agricultural Research (CGIAR), Montpellier, France; 26 Cuenca University, Cuenca, Ecuador; 27 Delhi School of Economics, University of Delhi, Delhi, India; 28 Departamento de Alimentacao Escolar, Sao Paulo, Brazil; 29 Department of Biomedical and Biotechnological Sciences, University of Catnia, Catania, Italy; 30 Department of CVD Epidemiology, Prevention and Health Promotion, Institute of Cardiology, Warsaw, Poland; 31 Department of Community Health Sciences, Aga Khan University, Karachi, Pakistan; 32 Diabetes Center, 2nd Department of Internal Medicine, Athens University, Athens, Greece; 33 Digestive Disease Research Institute, Tehran University of Medical Sciences, Tehran, Islamic Republic of Iran; 34 Directorate for Health Information & Research, Tarxien, Malta; 35 Dutch National Institute for Public Health and the Environment (RIVM), Bilthoven, Netherlands; 36 Eastern Mediterranean Public Health Network (EMPHNET), Amman, Jordan; 37 Egerton University, Njoro, Kenya; 38 Estonian National Institute for Health Development, Tallinn, Estonia; 39 FHI Solutions, Washington, DC USA; 40 FOSCAL and UDES, Bucaramanga, Colombia; 41 Faculty of Health Sciences, American University of Beirut, Beirut, Lebanon; 42 Faculty of Medicine, Eduardo Mondlane University, Maputo, Mozambique; 43 Faculty of Medicine, University of Colombo, Colombo 5, Sri Lanka; 44 Faculty of Medicine – Institute of Public Health, University of Porto, Porto, Portugal; 45 Faculty of Nutrition and Food Sciences, University of Porto, Porto, Portugal; 46 Finnish Institute for Health and Welfare, Helsinki, Finland; 47 Florida International University, Miami, FL USA; 48 Folkhälsan Research Center, Helsinki, Finland; 49 Food and Nutrition Research Institue (DOST-FNRI), Manila, Philippines; 50 Food and Nutrition Research Institue (DOST-FNRI), Taguig City, Philippines; 51 Fortis CDOC Center for Excellence for Diabetes, New Delhi, India; 52 FrieslandCampina, Amersfoort, The Netherlands; 53 Fundacion Cardiovascular de Colombia, Bucaramanga, Colombia; 54 Fundacion INFANT and Consejo Nacional De Investigaciones Cientificas y Tecnicas (CONICET), Buenos Aires, Argentina; 55 Fundacion Oftalmologica de Santander (FOSCAL), Floridablanca, Colombia; 56 Gachon University, Seongnam-si, South Korea; 57 Gazi University, Ankara, Turkey; 58 Geneva University Hospitals, Geneva, Switzerland; 59 Ghent University, Ghent, Belgium; 60 Global Dietary Database Consortium, Boston, MA USA; 61 Government of Canada, Statistics Canada, Ottawa, Ontario Canada; 62 Griffith University, Gold Coast, Queensland Australia; 63 HC Jehangir Medical Research Institute, Pune, India; 64 Hacettepe University Faculty of Medicine, Ankara, Turkey; 65 Hanoi Medical University, Hanoi, Vietnam; 66 Harokopio University, Athens, Greece; 67 Harvard School of Public Health, Cambridge, MA USA; 68 Hellenic Health Foundation and University of Athens, Athens, Greece; 69 Herbert Wetheim College of Medicine, Miami, FL USA; 70 Hirabai Cowasji Jehangir Medical Research Institute, Pune, India; 71 Hypertension Research Center, Cardiovascular Research Center, Isfahan University of Medical Sciences, Isfahan, Iran; 72 ICCAS (Instituto para la Cooperacion Científica en Ambiente y Salud), Buenos Aires, Argentina; 73 ICMR-National Institute of Nutrition, Hyderabad, India; 74 IRCCS Neuromed, Pozzilli, Italy; 75 Institut de Recherche pour le Developpement, Montpellier, France; 76 Institut de Veille Sanitaire, Bobigny, France; 77 Institute for International Investigation, NDRI-USA, New York, NY USA; 78 Institute of Biomedical Sciences, Academia Sinica, Taipei, Taiwan ROC; 79 Institute of Nutrition and Food Technology (INTA), University of Chile, Santiago, Chile; 80 Institute of Nutrition in Central America and Panama (INCAP), Guatemala City, Guatemala; 81 Institute of Public Health of Federation of Bosnia and Herzegovina, Sarajevo, Bosnia and Herzegovina; 82 Institute of Studies in Public Health, Federal University of Rio de Janeiro (UFRJ), Rio de Janeiro, Brazil; 83 Institutes of Biomedical Sciences, Academia Sinica, Taipei, Taiwan ROC; 84 International Agency for Research on Cancer, Lyon, France; 85 International Food Policy Research Institute (IFPRI), Washington, DC USA; 86 Interventional Cardiology Research Center, Cardiovascular Research Center, Isfahan University of Medical Sciences, Isfahan, Iran; 87 Intitut de Recherche en Sciences de la Sante, Bobo-Dioulasso, Burkina Faso; 88 Isfahan Cardiovascular Research Center, Cardiovascular Research Center, Isfahan University of Medical Sciences, Isfahan, Iran; 89 Israel Center for Disease Control, Ramat Gan, Israel; 90 Justus Liebig University Giessen, Giessen, Germany; 91 KLE Academy of Higher Education and Research (Deemed-to-be-University) Jawaharlal Nehru Medical College, Belagavi, India; 92 Kenya Medical Research Institute, Nairobi, Kenya; 93 King Abdulaziz University, Jeddah, Saudi Arabia; 94 King Saud University, Riyadh, Saudi Arabia; 95 King’s College London, London, UK; 96 Kinshasa School of Public Health, Kinshasa, Democratic Republic of Congo; 97 Korea Disease Control and Prevention Agency (KDCA), Cheongju, South Korea; 98 Korea University, Seoul, South Korea; 99 Kuwait Institute for Scientific Research, Kuwait City, Kuwait; 100 Lao Tropical and Public Health Institute, Vientiane, Lao PDR; 101 Lausanne University Hospital (CHUV) and University of Lausanne, Lausanne, Switzerland; 102 Leibniz Centre for Agricultural Landscape Research, Muncheberg, Germany; 103 Loyola University Chicago, Chicago, IL USA; 104 Mahidol University, Nakhon Pathom, Thailand; 105 Mahidol University, Bangkok, Thailand; 106 Malaysian Palm Oil Council (MPOC), Petaling Jaya, Malaysia; 107 Medical Center Markovs, Sofia, Bulgaria; 108 Menopause Andropause Research Center, Ahvaz Jundishapur University of Medical Sciences, Ahvaz, Islamic Republic of Iran; 109 Ministry of Health, Kuala Lumpur, Malaysia; 110 Ministry of Health, Sabak Bernam, Malaysia; 111 Ministry of Health, Victoria, Seychelles; 112 Ministry of Health, Jakarta, Indonesia; 113 NYU School of Medicine, New York, NY USA; 114 National Cancer Center Institute for Cancer Control, Tokyo, Japan; 115 National Centre of Public Health and Analyses (NCPHA), Sofia, Bulgaria; 116 National Food Agency, Uppsala, Sweden; 117 National Food and Nutrition Commission, Lusaka, Zambia; 118 National Food and Nutrition Institute, Warsaw, Poland; 119 National Health Research Institutes, Zhunan township, Taiwan ROC; 120 National Institute for Environmental Studies, Health and Environmental Risk Division, Tsukuba, Japan; 121 National Institute of Nutrition, Hyderabad, India; 122 National Institute of Nutrition, Hanoi, Vietnam; 123 National Institute of Nutrition and Food Technology & SURVEN RL, Tunis, Tunisia; 124 National Institute of Public Health (INSP), Mexico City, Mexico; 125 National Institute of Public Health (INSP), Cuernavaca, Mexico; 126 National Institutes of Biomedical Innovation, Health and Nutrition, Tokyo, Japan; 127 National Nutrition Institute, Cairo, Egypt; 128 National Nutrition and Food Technology Research Institute (NNFTRI): SBMU, Tehran, Islamic Republic of Iran; 129 National University of Malaysia (UKM), Kuala Lumpur, Malaysia; 130 Nestlé Research, Lausanne, Switzerland; 131 New England Complex Systems Institute, Cambridge, MA USA; 132 North-West University, Potchefstroom, South Africa; 133 Oslo Metropolitan University (OsloMet), Oslo, Norway; 134 Perdana University, Puchong, Malaysia; 135 Royal College of Surgeons in Ireland, Dublin, Ireland; 136 Pontificia Universidad Javeriana Seccional, Cali, Colombia; 137 Public Authority for Food and Nutrition, Sabah Al Salem, Kuwait; 138 Public Health Authority of the Slovak Republic, Bratislava, Slovak Republic; 139 Qatar University and University of Jordan, Doha, Qatar; 140 Research Institute for Endocrine Sciences, Shahid Beheshti University of Medical Sciences, Tehran, Islamic Republic of Iran; 141 Research Institute for Primordial Prevention of NCD, Isfahan University of Medical Sciences, Isfahan, Islamic Republic of Iran; 142 Risk and Benefit Assessment Department, Swedish Food Agency, Uppsala, Sweden; 143 Robert Koch Institute, Berlin, Germany; 144 Rutgers University, New Brunswick, NJ USA; 145 Santé publique France, the French Public Health Agency, Saint Maurice, France; 146 Seoul National University, Seoul, South Korea; 147 St John’s Research Institute, Bangalore, India; 148 Tabriz University of Medical Sciences, Tabriz, Islamic Republic of Iran; 149 Tallinn University, Tallinn, Estonia; 150 Taylor’s University, Subang Jaya, Malaysia; 151 Teesside University, Middlesbrough, UK; 152 Tehran University of Medical Sciences, Tehran, Islamic Republic of Iran; 153 Tehran University of Medical Sciences and Utica University, Tehran, Islamic Republic of Iran; 154 The Technical University of Kenya, Nairobi, Kenya; 155 The University of New Mexico, Albuquerque, NM USA; 156 Tropical Diseases Research Centre, Ndola, Zambia; 157 Unidad de Nutricion Publica, Macul, Chile; 158 Department of Biochemistry, University of Puerto Rico – Medical Science Campus, San Juan, Puerto Rico; 159 Universidad San Sebastian, Santiago, Chile; 160 Universidad Tecnica del Norte, Ibarra, Ecuador; 161 Universidad de Antioquia, Medellin, Colombia; 162 Universidad de Cuenca, Cuenca, Ecuador; 163 Universiti Kebangsaan Malaysia Medical Centre, Kuala Lumpur, Malaysia; 164 Universiti Putra Malaysia, Serdang, Malaysia; 165 Universiti Sains Malaysia, Kubang Kerian, Malaysia; 166 University Center for Primary Care and Public Health (Unisanté), Lausanne, Switzerland; 167 University Dunarea de Jos, Galati, Romania; 168 University Kebangsaan Malaysia, Bangi, Malaysia; 169 University of Aberdeen, Aberdeen, UK; 170 University of Alberta, Edmonton, Alberta Canada; 171 University of Bergen, Bergen, Norway; 172 University of Bonn, Department of Nutrition and Food Sciences, Bonn, Germany; 173 University of California Davis, Davis, CA USA; 174 University of Cincinnati, Cincinnati, OH USA; 175 University of Colombo, Colombo, Sri Lanka; 176 University of Colorado School of Medicine, Aurora, CO USA; 177 University of Dhaka, Dhaka, Bangladesh; 178 University of Goettingen, Goettingen, Germany; 179 University of Helsinki, Department of Food and Nutrition, Helsinki, Finland; 180 University of Hohenheim, Stuttgart, Germany; 181 University of Iceland, Reykjavík, Iceland; 182 University of Insubria, Varese, Italy; 183 University of Las Palmas de Gran Canaria (ULPGC), Las Palmas, Spain; 184 University of Malaya, Kuala Lumpur, Malaysia; 185 University of Manchester, Manchester, UK; 186 University of Mauritius, Moka, Mauritius; 187 University of Medicine and Pharmacy Carol Davila, Bucharest, Romania; 188 University of North Carolina at Chapel Hill, Chapel Hill, NC USA; 189 University of Otago, Dunedin, New Zealand; 190 University of Sao Paulo, Sao Paulo, Brazil; 191 University of Saskatchewan, Saskatoon, Saskatchewan Canada; 192 University of Vienna, Vienna, Austria; 193 University of the Southern Caribbean, Port-of-Spain, Trinidad and Tobago; 194 Wageningen University, Wageningen, Netherlands; 195 Washington University in St. Louis, St. Louis, MO USA; 196 World Health Organization (WHO), Geneva, Switzerland; 197 World Health Organization (WHO), Amman, Jordan; 198 Yazd Cardiovascular Research Centre, Shahid Sadoughi University of Medical Sciences, Yazd, Islamic Republic of Iran; 199 Ziauddin University Karachi, Karachi, Pakistan

**Keywords:** Risk factors, Epidemiology

Correction to: *Nature Food* 10.1038/s43016-022-00594-9. Published online 19 September 2022.

In the version of this article originally published, the Global Dietary Database consortium was missing from the author list. The consortium is now listed as an author, with a list of members and their affiliations appearing online. The error has been corrected in the HTML and PDF versions of the article.

