# Supplementary material for: Global dietary quality in 185 countries from 1990 to 2018 show wide differences by nation, age, education, and urbanicity
Source: Nat Food. 2022 Sep 19;3(9):694–702. doi: 10.1038/s43016-022-00594-9 (PMC10277807; doi:10.1038/s43016-022-00594-9)
Supplement: Supplementary file 1 — Supplementary methods, Tables 1–8 and discussion. [file 43016_2022_594_MOESM1_ESM.pdf]

nature portfolio

https://doi.org/10.1038/s43016-022-00594-9

Supplementary information

Global dietary quality in 185 countries from 1990 to 2018 show wide differences by nation, age, education, and urbanicity

In the format provided by the authors and unedited

Table of Contents

Description of data assessment, standardization, and analysis....2
Table S1. Definitions and units of dietary variables included in the GDD....7
Table S2. Countries, regions, and super-regions included in GDD 2018 (N=185)....9
Covariates....12
GDD Prediction Model....14
Intercept, sex differences, education differences, and urban/rural differences....16
Covariate effects....17
Age trend....17
Overdispersion....18
Computation....18
Predictions....18
Varying slopes modeling structure....21
Flowchart of the number of countries with identified and standardized dietary surveys and included in the Bayesian hierarchical model....23
Table S3. Characteristics of global data sources of dietary pattern components in children and adults....24
Table S4. Characteristics of total dietary surveys (N=1,139) used to model the dietary pattern components....31
Table S5. Number of dietary surveys for diet factor overall and by year....33
The Global Dietary Database Corresponding Members....34
Table S6. AHEI scoring thresholds....37
Table S7. DASH scoring thresholds....38
Table S8. MED scoring thresholds....38
Global, regional, and national DASH and MED scores in 2018....39

Description of data assessment, standardization, and analysis

Data assessment

Data received from corresponding members or from publicly available surveys were checked to confirm survey-level characteristics; dietary intake variables were categorized into GDD dietary factors; necessary unit and format conversions were noted. Data-owners or survey directors were contacted extensively to resolve questions about data quality, categorization, or assessment methods to ensure accuracy and completeness of data prior to analysis.

Preliminary data checks

Biostatisticians generated survey description files for each survey including survey characteristics, variable lists, and summary statistics for categorical and continuous variables. Research assistants used these description files to assess inclusion of survey level information and demographic variables. Discrepancies between author-reported characteristics and those ultimately included in the dataset were noted for further discussion with the data-owner.

Categorization of variables into GDD dietary factors

Research assistants matched reported dietary data to GDD dietary factors. This involved categorizing foods, nutrients, mixed dishes, and regional items into the matched GDD dietary factor, noting cases where variables represented less than the optimal GDD definition. Unit conversions were included as necessary to transform variables into the optimal GDD units.

Categorization of variables into GDD dietary factors – Food Frequency Questionnaires

To transform food and beverage data reported from Food Frequency Questionnaires into optimal GDD units, most often grams per day, all categorical variables were standardized into single daily serving units. When ranges of frequencies were provided, the mean of each range was utilized to represent each frequency category. Variables reported in times per week were divided by 7 to calculate the average daily servings. Variables reported in times per month were divided by 30·42 (the average number of days in a month) to calculate the average daily servings. In cases where the upper range was open-ended (e.g., "5 or more times per week"), the ranges of the other frequency categories were used to calculate an upper limit. Servings per day were then converted into grams per day by multiplying the number of servings by the author-reported serving sizes or by the GDD standard serving sizes.

Communication with data owners and creation of data key

Any questions regarding the data, including those about region-specific diets (e.g., disaggregation of mixed dishes, classification of regional items), survey-level characteristics, and serving sizes for foods and beverages, were communicated to the data owner. After all questions regarding the data were answered, research assistants generated a data key outlining all available variables of interest, including demographic and dietary variables. Categorizations of dietary variables were turned into STATA code for clear identification and research assistants flagged the quality assurance checks.

Converting household data to individual data

Household-level data were transformed into individual-level data using the Adult Male Equivalent (AME) method. The AME method estimates individual-level intakes by assigning each household member a reference AME based on their age and sex. Household members' reference AMEs are summed to find total household AME. Each individual's reference AME is then divided by the total household AME to find individual-level AME. This individual-level AME represents the proportion each individual contributes to the overall household AME. This individual level proportion is multiplied by the household consumption of each food item to estimate individual-level intake.

Data aggregation

Using preliminary checking documents provided by research assistants, biostatisticians converted individual-level data into aggregated outputs for each dietary factor stratified by the available demographic variables. Stata version 12 was used to convert all demographic and dietary variables from raw data files to a single data file containing only relevant variables. Missing observations were excluded from the dataset and all variables were recoded to match the GDD demographic and dietary variable coding scheme. Data were then aggregated into demographic strata by age, sex, residence, education, and pregnancy/lactating status. In addition to the single, final data file, supporting files were generated including a summary report, minimum/maximum values for each dietary factor, and group level means, standard deviations, and percentiles of intake. All output files were stored in each survey's specific folder on the Tufts GDD Box, accessible to all members of the research team.

Energy adjustment

We extracted both raw and energy-adjusted data when available. If energy adjustment of individual-level data had not been completed by the data owner, biostatisticians completed energy adjustment at the aggregation stage to age-specific levels using the residual method. This approach was considered the "gold standard." We adjusted for total energy intake to mitigate the effects of measurement error in data collection, account for differences in energy

requirements related to body size, metabolic efficiency, and physical activity, and facilitate comparisons between surveys, age groups, and sexes.

Total daily energy values by age

Age (years)	Daily energy intake (kcal)
<1	700
1-2	1,000
2-5	1,300
6-10	1,700
11-74	2,000
75+	1,700

Child and older adult-specific daily energy values were selected using dietary recommendations and mean population ranges from the USA, United Kingdom, and India.

Energy adjustment corrections

We initially asked that all data be shared both unadjusted and energy-adjusted to 2,000 kcal, regardless of age category, but retrospectively changed this decision to reflect the age-specific levels. When possible, energy adjustment using the residual method was repeated to reflect these changes. In some cases, this approach was not possible, and thus alternative approaches for energy adjustment correction were taken.

Energy adjustment correction of aggregate ("stratum-level") data

In some cases, data were provided or accessed at the stratum level (i.e., age group, sex, education level, etc.). In these cases, energy adjustment correction depended on whether 2,000 kcal/day-adjusted values had previously been provided by the data owner. If energy-adjusted data had been provided, a simple ratio of the age-specific level to 2,000 kcal was applied post-hoc to convert the value to the correct energy level. If stratum-level data were only provided in an unadjusted format but with corresponding total energy intake, intake was adjusted to the age-specific energy level using the energy density method, in which a simple ratio of reported calorie intake to age-specific level was applied to the unadjusted value. If stratum-level data were provided in age groups which traversed more than one level of age-specific energy intake, a weighted mean daily energy intake was calculated. This weighted mean daily energy level was then used to adjust intake using the ratio readjustment method. If only unadjusted intake was available, the energy density method was used.

Energy adjustment of data without adjusted values or total energy intake

In limited cases, individual-level data were not initially energy-adjusted or provided with mean caloric intake data, precluding the use of the gold standard and ratio readjustment methods. In these instances, daily per capita energy availability data from FAO Food Balance Sheets (FBS) were used to inform stratum-level caloric intake. In short, country-year-specific FBS energy data were adjusted using coefficients derived from a multivariate linear regression of GDD input data, FBS data, and both regional and survey-level covariates. Adjusted FBS energy was then corrected to the prescribed energy level by applying a factor of the energy level's proportion of 2,000 kcal. Unadjusted food and nutrient intake values were then adjusted with this corrected energy intake via the energy density method.

Quality control

Data integrity and quality were assessed at each step during survey collection, processing, harmonization, and analyses. Duplicate reviews were performed of recorded survey characteristics, demographic variables, dietary definition classifications, and unit conversions. To assess for outliers and validity (errors) in reported intakes, plausibility thresholds were defined for each dietary factor, both at the individual level and stratum (e.g., group mean) level, based on dietary reference intakes, tolerable upper limits, toxicity ranges, and existing regional data on mean intakes in populations. Any value identified as potentially implausible was reviewed for extraction errors, followed by direct correspondence with the corresponding member or public survey data owners, to detect and correct potential errors. Data remaining implausible after such steps were excluded from final datasets. Results for each dietary factor were further graphed and visually inspected by country, age, sex, dietary assessment method, representativeness, and time, reviewing survey result plausibility and consistency within and across countries.

Data finalization

After data has been finalized for inclusion, it is stored within the Access database, which houses information on all surveys, corresponding authors, and survey checking statuses. Aggregated data is collated by dietary factor and prepared for input into the GDD prediction model.

Protocol for converting FFQ frequency data into GDD servings

1. Step 1- Standardize the categorical frequency variables to a single daily serving unit
    a. If a range of frequencies is given, take the mean (“Avg”) of the range
    b. If the frequency is presented in times/week, divide by 7 (for days in a week)
    c. If the frequency is presented in times/month, divide by 30.42 (average days in a month)
        i. ***Note:*** If the category is presented as days/week instead of times/week, assume one serving per day and treat as times/week
        ii. Example A) 5-7 days/week = (6 days/week) / (7days/week) = 0.857 servings/day
        iii. Example B) 1-3 times/month = (2 times/month) / (30.42 days/month) = 0.066 servings/day
    d. If the upper range is open ended, use the range of the other frequency categories in the survey to create an upper limit and then take the average of that range.
        i. Example: “5 or more times per week” where next lowest level is 2-4 times per week. Assume a range of 5-7 times per week, take the average (6 times per week)/(7 days/week) = 0.857 servings/day
2. Step 2- Convert servings to grams
    a. If available, survey-specific serving sizes were used for conversions.
    b. If survey-specific serving sizes are not available, ask the data owner for usual, country-specific serving sizes.
    c. If data owner does not provide country-specific serving sizes, utilize country-specific serving sizes identified from national agencies (e.g., USDA).
    d. If no country-specific serving sizes are identified, use the GDD standard serving size conversions.

Common categories of intake and their servings per day conversions

Categorical Variable	Calculation	Daily Serving
Never	0	0
Occasional-Few times/year*	Should capture the range of values between never and the next highest choice based on the data set	*Depends on next level categorization
Less than once a month (1-11 times per year)	1+11=12/2=6 Avg servings/year<br>6/12 months=0.5 servings/month<br>0.5/30.42 days	0.0164
1-3 times/month	1+3=4/2=2 Avg servings/month<br>2/30.42 days	0.066
1/week	1 servings/7 days	0.143
2-4 days/week	2+4=6/2=3 Avg servings/week<br>3servings/7 days	0.429
5-6 days/week	5+6=11/2= 5.5 Avg servings/week<br>5.5/7 days	0.786
5-7 days/week	5+7=12/2=6 Avg servings/week<br>6/7 days	0.857
1/day		1
2-3/day	2+3=5/2= Avg 2.5 servings	2.5
4-5/day	4+5=9/2= Avg 4.5 servings	4.5

Common weight conversions

**Provided weight**	**Grams**
1 Kilogram	1000
1 Ounce *Cannot use for fluid ounces	28
1 Pound	454

Standard serving sizes for foods and beverages

Dietary factor		Reference serving sizes	"Usual" average serving sizes (g/serving)		
**Variable Code**	**Variable name**	**2003-06 US NHANES (median)**	**Adults and children older than 2 years**	**12-24 months**	**6 to 11 months**
v01	Fruits°	110 g per serving	100	75	49
v02	Non-starchy vegetables°	40 g per serving	100	50	44
v05	Beans/legumes°	86.5 g per serving	100	32	24
v06	Nuts/seeds	29.75 g per serving	28.35	32	24
v08	Whole grains	48.975 g per serving	50	39	12
v09	Processed meats	53.705 g per serving	50	41	31
v10	Red meats	85 g per serving	100	32	24
v11	Seafood*	85.78 g per serving	100	30	23
v13	Cheese*	-	42	22	20
v14	Yogurt*	-	245	104	88
v15	Sugar-sweetened beverages*	368 g per serving	248	130	84
v16	Fruit juices*	209.25 g per serving	248	130	84
v57	Milk	198.25 g per serving	245	161	155

*Calculated using average of item-specific serving sizes from the USDA Nutrient Database.
°Calculated using average if both item-specific serving sizes from the USDA Nutrient Database and intake from NHANES 2003-2006.

Table S1. Definitions and units of dietary variables included in the GDD.

Dietary factor	Unit	Preferred definition	Alternative definition
Fruits	g/day	Total fruit intake, including fresh, frozen, cooked, canned, or dried fruit, excluding fruit juices and salted or pickled fruits.	Total fruit intake including fruit juices, nuts/seeds, vegetables, salted/pickled, preserved, and processed fruits (jams).
Non-starchy vegetables	g/day	Total vegetable intake, including fresh, frozen, cooked, canned, or dried vegetables. This definition excludes salted or pickled vegetables, vegetable juices, starchy vegetables (e.g., potatoes, taro, cassava, manioc, yucca, corn, peas), and legumes (beans and lentils).	Total vegetable intake including vegetable juices, starchy vegetables, nuts/legumes, nuts/beans, beans/legumes, salted/pickled vegetables, and salted/pickled beans/legumes.
Beans and legumes	g/day	Total intake of beans and legumes (beans, lentils), including fresh, frozen, cooked, canned, or dried beans/legumes. This definition excludes peanuts and peanut butter. This definition includes soybeans but excludes soy milk and soy protein.	Includes nuts/seeds, soy protein, soy products, peanuts, and peas.
Nuts and seeds	g/day	Total intake of tree nuts (e.g., walnuts, almonds, hazelnuts, pecans, cashews, pistachios), seeds (e.g., sesame seeds, sunflower seeds, pumpkin seeds), and peanuts (including peanut butter).	Includes pulses, beans, legumes, and foods primarily (>51%) from nuts or seeds.
Whole grains	g/day	Total intake of whole grains, defined as a food with ≥1.0 grams of fiber per 10 grams of carbohydrate, in which all components of the kernel (i.e., bran, germ, and endosperm) are present in the same relative proportions as the intact grain. Examples include whole grain bread, brown rice, whole grain pasta, whole grain breakfast cereals, oats, rye, barley, millet, sorghum, and bulgur. This definition excludes corn products including corn flour, corn meal, and popcorn.	Includes wholegrain breads, cereals, rice/pasta, bread, and other products such as biscuits.
Total processed meats	g/day	Total intake of processed meat, defined as any meat (including poultry) that has been cured, smoked, dried, or chemically preserved. Examples include bacon, salami, sausages, hot dogs, and processed deli or luncheon meats. This definition excludes fish and eggs.	Includes sausages and unprocessed meats.
Unprocessed red meats	g/day	Total intake of unprocessed red meat, defined as beef, pork, lamb, mutton, or game that has not been cured, smoked, dried, or chemically preserved. This definition excludes poultry, fish, and eggs.	Includes processed red meats, poultry, fish and organ meats.
Total seafoods	g/day	Total intake of fish and shellfish. Examples include salmon, tuna, trout, tilapia, shrimp, crab, oysters, and cephalopods.	Includes salted fish, processed fish, and other animal products.
Cheese	g/day	Total intake of cheese derived from the milk of livestock (e.g., cows, buffalo, yak), including hard cheese (e.g., cheddar,	Includes yogurt, milk products and cheese.

		mozzarella, Swiss), soft cheese (e.g., ricotta, cottage cheese, paneer), and processed cheese.	
Yogurt	g/day	Total intake of yogurt and fermented milk, including reduced-fat and full-fat yogurt.	Includes dairy curd, buttermilk, paneer, cheese, and milk.
Sugar-sweetened beverages (SSBs)	g/day	Total sugar-sweetened beverage intake, defined as any beverage with added sugar having ≥50 kcal per 8 ounces (236.5 grams) serving, including commercial or homemade beverages, soft drinks, energy drinks, fruit drinks, punch, lemonade, and frescas. This definition excludes 100% fruit and vegetable juices and non-caloric artificially sweetened drinks.	Includes fruit and vegetable juices. May also include coffee, tea, and milk.
Fruit juices	g/day	Total intake of 100% fruit juice, excluding sugar-sweetened fruit juice and vegetable juice.	Includes fruit juices, vegetable juices and sweetened juices.
Total milk	g/day	Total intake of dairy milk including non-fat, low-fat, skim, and whole-fat milk. This definition excludes yogurt, fermented milk, and soy or other plant derived milk (e.g., coconut milk, almond milk).	Includes yogurt, dairy drinks, cheese, and dairy products.
Saturated fat	Percent energy/day	Total saturated fat intake from all sources (primarily meat and dairy products, and tropical oils).	
Monounsaturated fat	Percent energy/day	Total monounsaturated fat intake from all sources.	
Total omega-6 fatty acids	Percent energy/day	Total omega-6 fatty acid intake from all sources (primarily liquid vegetable oils, including soybean oil, corn oil and safflower oil), excluding dietary supplements.	Includes total polyunsaturated fat or linoleic acid.
Seafood omega-3 (n-3) fat	mg/day	Total dietary EPA+DHA (eicosapentaenoic acid + docosahexaenoic acid) intake, excluding dietary supplements.	Includes total dietary EPA+DPA+DHA (eicosapentaenoic acid + docosahexaenoic acid + docosapentaenoic acid), long chain omega-3 only, excluding ALA (alpha-linolenic acid) and total seafood intake (fish & shellfish).
Plant omega-3 (n-3) fat	mg/day	Total dietary ALA (alpha-linolenic acid) intake, excluding dietary supplements.	Includes ALA (alpha-linolenic acid) + long chain omega-3 (EPA, DPA, DHA) (eicosapentaenoic acid, docosahexaenoic acid, docosapentaenoic acid)
Dietary sodium	mg/day	Total intake of sodium from all sources.	Includes urinary sodium.

Table S2. Countries, regions, and super-regions included in GDD 2018 (N=185).

Region	Countries
**Southeast and East Asia super-region (N=24)**	
East Asia (N=2)	China, Taiwan
Southeast Asia (N=9)	Cambodia, Indonesia, Lao People's Democratic Republic, Malaysia, Myanmar, The Philippines, Thailand, Timor-Leste, Viet Nam
Asia-Pacific high income (N=4)	Brunei Darussalam, Japan, Republic of Korea, Singapore
Oceania (N=9)	Fiji, Kiribati, Marshall Islands, Micronesia, Papua New Guinea, Samoa, Solomon Islands, Tonga, Vanuatu
**Central/Eastern Europe and Central Asia super-region (N=29)**	
Central Asia (N=9)	Armenia, Azerbaijan, Georgia, Kazakhstan, Kyrgyzstan, Mongolia, Tajikistan, Turkmenistan, Uzbekistan
Central Europe (N=13)	Albania, Bosnia and Herzegovina, Bulgaria, Croatia, Czech Republic, Hungary, Montenegro, Poland, Romania, Serbia, Slovakia, Slovenia, The former Yugoslav Republic of Macedonia
Eastern Europe (N=7)	Belarus, Estonia, Latvia, Lithuania, Republic of Moldova, Russian Federation, Ukraine
**Latin America and Caribbean super-region (N=32)**	
Caribbean (N=15)	Antigua and Barbuda, Bahamas, Barbados, Belize, Cuba, Dominica, Dominican Republic, Grenada, Guyana, Haiti, Jamaica, Saint Lucia, Saint Vincent and the Grenadines, Suriname, Trinidad and Tobago
Andean Latin America (N=3)	Bolivia, Ecuador, Peru
Central Latin America (N=9)	Colombia, Costa Rica, El Salvador, Guatemala, Honduras, Mexico, Nicaragua, Panama, Venezuela
Southern Latin	Argentina, Chile, Uruguay

America (N=3)	
Tropical Latin America (N=2)	Brazil, Paraguay
**Middle East and Northern Africa super-region (N=20)**	
Western Europe (N=1)	Israel
North Africa and Middle East (N=18)	Algeria, Bahrain, Egypt, Iran (Islamic Republic of), Iraq, Jordan, Kuwait, Lebanon, Morocco, Occupied Palestinian Territory, Oman, Qatar, Saudi Arabia, Syrian Arab Republic, Tunisia, Turkey, United Arab Emirates, Yemen
**South Asia super-region (N=8)**	
South Asia (N=6)	Afghanistan, Bangladesh, Bhutan, India, Nepal, Pakistan
Southeast Asia (N=2)	The Maldives, Sri Lanka
**Sub-Saharan Africa super-region (N=48)**	
Central Sub-Saharan Africa (N=6)	Angola, Central African Republic, Congo, Democratic Republic of Congo, Equatorial Guinea, Gabon
Eastern Sub-Saharan Africa (N=17)	Burundi, Comoros, Djibouti, Eritrea, Ethiopia, Kenya, Madagascar, Malawi, Mauritius, Mozambique, Rwanda, Seychelles, South Sudan, Sudan, Uganda, United Republic of Tanzania, Zambia
Southern Sub-Saharan Africa (N=6)	Botswana, Lesotho, Namibia, South Africa, Swaziland, Zimbabwe
Western Sub-Saharan Africa (N=19)	Benin, Burkina Faso, Cameroon, Cape Verde, Chad, Côte d'Ivoire, The Gambia, Ghana, Guinea, Guinea-Bissau, Liberia, Mali, Mauritania, Niger, Nigeria, São Tomé and Príncipe, Senegal, Sierra Leone, Togo
**High-Income Countries super-region (N=24)**	
Australasia (N=2)	Australia, New Zealand

Western Europe (N=20)	Austria, Belgium, Cyprus, Denmark, Finland, France, Germany, Greece, Iceland, Ireland, Italy, Luxembourg, Malta, Netherlands, Norway, Portugal, Spain, Sweden, Switzerland, United Kingdom
North America high-income (N=2)	Canada, United States of America

We included countries: 1) classified as United Nations (UN) Member States, 2) included in the United Nations Food and Agriculture Food Balance Sheets database, or 3) included in the World Bank Gross Domestic Product database.

Covariates

Covariate identification

We identified country- and time-specific covariate data from various sources to further inform our model predictions. These data supplement our individual-level dietary intake data, particularly in countries for which these inputs are limited. We consulted experts and conducted comprehensive searches of publicly available databases to identify >800 covariates. We prioritized approximately 400 covariates for testing:

Data source	Year(s)
UN FAO food balance sheets	1980 - 2018
Harvard Global Expanded Nutrient Supply (GENuS)	1980 - 2011
Principal component analysis of FAO and GENuS data	2013
Euromonitor fat and oils sales data	1998 - 2018
World Bank Gross Domestic Product (GDP)	1980 - 2018
World Bank unemployment rate	1980 - 2015
World Bank gini coefficient	1980 - 2015
World Bank poverty rate	1980 - 2015
Barro Lee years of schooling	1980 - 2010
World Bank precipitation	1982 - 2014
CIA Factbook latitude	N/A
CIA Factbook land area	N/A
CIA Factbook coastline ratio	N/A

We conducted principal component analysis (PCA) using the 'princomp' function in R separately for: 1) 23 grouped FAO food balance sheet (FBS) foods, beverages, and energy, 2) 142 GENuS foods, and beverages, and 3) 19 GENuS nutrients and energy. The first four components from each PCA were considered for covariate testing.

Covariate imputation and truncation

If covariate data were missing for some (but not all) years of a given country, we used linear interpolation to fill in those years. Covariate data sources that ended before 2018 were imputed using a moving average of the three most recent values to obtain values for all covariates through the year 2018. Region-level means were assigned to countries for which entire covariates were missing. To assess validity of the imputations, we imputed non-missing values with the same model and visually compared observed versus imputed values via scatter plots.

The GDD prediction model operates on the natural log scale (except for dietary factors measured as proportions), including the covariate data. To reduce the risk of having very small values for covariates with a broad range of values on the log scale having an outsized influence on modeled estimates, we truncated covariate data on the non-transformed scale using the following rules:

For covariates with a 95th percentile value

1. >3.5: Truncate values <0.5 to 0.5
2. ≥1 and ≤3.5: Truncate values <0.1 to 0.1
3. <1: No truncation

Covariate testing
For each dietary factor, we calculated the correlations between covariates and original survey-level stratified mean dietary intakes, and we selected up to 10 covariates for model inclusion, favoring those with the highest correlations, a mix of food/nutrients and other covariates, and sensible links to the dietary factor.

Each of the covariates identified in the correlation stage (maximum 10 covariates) and the four PCA components were then included in a stepwise regression (entry point of $p<.299$ and exit point of $p<.30$) to test for inclusion in GDD models. These stepwise regressions resulted in three nested versions of the GDD model per diet factor:

1. **Base model:** Closest diet factor proxy from FAO or GENuS (1-2 covariates per model)
2. **Restricted model:** All covariates with $p<0.1$ from the results of the stepwise regression plus base model covariate(s).
3. **Inclusive model:** All covariates from the results of the stepwise regression plus base model covariate(s).

For each dietary factor, five-fold cross-validation was used to compare model fit for the three versions of the GDD model. Data were split into five partitions at the survey level: four partitions making up the training dataset, and the remaining segment as the testing data. The models were fit to the training set, and resulting outputs were compared to training set to assess model fit via calculating the expected log predictive density (ELPD)[1]. This was repeated five times so that each partition was used once as the training set.

Final model selection and included covariates by dietary factor.

Dietary factor	Selected model	Covariates
Fruit	Base model	FAO fruit
Non-starchy vegetables	Base model	FAO vegetables
Whole grains	Base model	FAO whole grains
Nuts	Base model	FAO nuts
Legumes	Base model	FAO pulses
Unprocessed red meat	Base model	FAO red meat
Processed meat	Restricted model	FAO processed meat; FAO potatoes; GENuS iron; FAO PCA 1; FAO PCA 2; GENuS carbohydrates; FAO alcoholic beverages
Seafood	Base model	FAO fish and seafood
Milk	Base model	FAO milk
Cheese	Base model	FAO cheese
Yogurt	Base model	FAO cheese
Sugar-sweetened beverages	Base model	FAO sugar and sweeteners
Fruit juice	Base model	FAO fruit
Saturated fat	Base model	GENuS saturated fat
Monounsaturated fat	Base model	GENuS monounsaturated fat
Omega-6 fat	Base model	GENuS polyunsaturated fat
Seafood omega-3 fat	Base model	GENuS polyunsaturated fat
Plant omega-3 fat	Base model	GENuS polyunsaturated fat
Sodium	Base model	GENuS sodium

References
1. Vehtari, A., Gelman, A. & Gabry, J. Practical Bayesian model evaluation using leave-one-out cross-validation and WAIC. *Stat Comput* **27,** 1413–1432 (2017). https://doi.org/10.1007/s11222-016-9696-4.

GDD Prediction Model

1. Overview

The GDD prediction model aims to estimate mean intake of 53 dietary factors in 185 countries, by country/year/age/sex/urbanicity/education, by synthesizing survey mean intake data from sources of varying quality. The Bayesian multilevel framework has some advantageous properties that are appealing for our purposes. Namely,

- "Shrinkage" of parameter estimates towards an overall mean. For example, mean estimates for data sparse countries are pulled towards the region mean, allowing for more reasonable estimates for countries with potentially unreliable data.
- Intuitive framework for predicting means (with uncertainty bounds) for countries with no available data.
- Ability to include prior knowledge about intake through priors
- Allows for model flexibility and complexity often not granted in similar frequentist approaches due to difficulty in optimization.

2. Hierarchical nature of the data

Survey data collected across the globe have an inherently nested hierarchical structure which makes a multilevel approach to modeling the data appealing. The hierarchical structure of the data we assumed was as follows: countries were nested in super-regions, which are nested in the globe. Our model assumed that the super-region means were distributed log-normally around the global mean, and that country means were distributed log-normally around their respective super-region means. Using this structure allowed us to borrow strength across units, a concept commonly known as "partial pooling" in the Bayesian literature. In partial pooling, each country's mean estimate borrows from the other countries' data within the region, resulting in shrinkage of the country mean estimate towards the region mean. The less informative the data was for a particular country, the more pooling there is.

Our model used the following seven super-regions:

a. Asia (Southeast and East Asia)

b. FSU (Central/Eastern Europe and Central Asia)

c. HIC (High Income Countries)

d. LAC (Latin America and Caribbean)

e. MENA (Middle East and North Africa)

f. SAARC (South Asia)

g. SSA (Sub-Saharan Africa)

3. Description of model

Fundamentally, our model was a Bayesian model on the log-means of intake with a nested hierarchical structure (clusters countries within super-regions and super-regions within the globe), assuming exchangeability between countries and super-regions after accounting for covariates. To this structure, we added sex, urban/rural, education, and non-linear age effects (also within a nested hierarchical structure), survey and country-level covariates, and overdispersion on study-level variance to account for non-sampling

variation. It borrowed heavily from models presented by Finucane et al.[1] and Flaxman et al.[2]. For dietary factors that were measured as proportions of energy intake, we used $-log(-log(y))$ as the link function instead of $log(y)$.

Below we provide a full mathematical description of the model, with detailed descriptions for each component, but first, we present some notation:

a. Subscript notation:

   i. h: age/sex/educ/urbanicity group
   ii. i: study
   iii. j: country
   iv. k: super-region

b. Superscript notation:

   i. c: country
   ii. s: super-region
   iii. g: globe

The model

$$\boldsymbol{f}(y_{h,i}) \sim N\left(a_j + b_{1j}sex_{h,i} + b_{2j}u_{h,i} + b_{3j}educ_{h,i} + \gamma_j(z_{h,i}) + \boldsymbol{X}_i\boldsymbol{\beta}, SE^2_{h,i} + \tau^2\right)$$

where

$f(y)$ ⇐$-log(-log(y))$ for dietary factors measured as proportions, $log(y)$ otherwise

$y_{h,i}$ ⇐mean intake level for stratum h in study $i$$a_j$ ⇐ country-specific intercept

b_{1j} ⇐country-specific difference between females and males

$sex_{h,i}$ ⇐variable indicating whether the $y_{h,i}$ corresponds to an all male group (0), all female group (1),or mixed (0.5)

b_{2j} ⇐region-specific difference between urban and rural

$u_{h,i}$ ⇐variable indicating whether the $y_{h,i}$ corresponds to an all rural group (0), all urban group (1),or mixed (% urban)

b_{3j} ⇐region-specific education effect

u_i ⇐two variables indicating whether $y_{h,i}$ corresponds to low education (defined to be 6 years or less of schooling if mixed, proportion of low education and high education

γ_j ⇐non-linear age-trend for region j

$z_{h,i}$ ⇐midpoint age of stratum h in study i

$\boldsymbol{X}_i\beta$ ⇐study + country level covariate effects

$SE^2_{h,i}$ ⇐standard error of $f(y_{h,i})$ (estimated via delta method)

τ^2_i ⇐overdispersion parameter for study i

Intercept, sex differences, education differences, and urban/rural differences

We fit a multi-level model with 3 levels (countries nested in super-regions nested in the globe) for intercepts and sex differences, and 2 levels (super-regions nested in the globe) for age pattern, education differences, and urban/rural differences. a_j refers to the intercept for country j, b_{1j} refers to the country specific sex effect, b_{2j} refers to the country specific urban effect, and b_{3j} refers to the country specific education effect. a^g and b^g correspond to global intercept and effects while a^s, b^s denote super-region specific random effects and a^c, b^c denote country specific random effects. κ^c and κ^s are the between-country and between-super-region variance, respectively, for their respective model components. Note that the model assumes between country variance is the same across all super-regions, and that education, urban/rural differences and age patterns are assumed to be the same for countries within a super-region.
Mathematically, this can be described as follows:

$$a_j = a^c_j + a^s_{k[j]} + a^g$$
$$b_{1j} = b^c_{1j} + b^s_{1k[j]} + b^g_1$$
$$b_{2j} = b^s_{2k[j]} + b^g_2$$
$$b_{3j} = b^s_{3k[j]} + b^g_3$$

$$a^c_j \sim N(0, \kappa^c_a),\ b^c_{1j} \sim N(0, \kappa^c_{1b}),$$
$$a^s_k \sim N(0, \kappa^s_a),\ b^s_{1k} \sim N(0, \kappa^s_{1b}),\ b^s_{2k} \sim N(0, \kappa^s_{2b}),\ b^s_{3k} \sim N(0, \kappa^s_{3b})$$

Weakly informative priors were used for the hyper-parameters: half-Normal(0, 0.5) for the κ parameters, For a^g, b^g_1, b^g_2, and b^g_3, a prior of N(0, 0.35) was used. Input data were standardized to the standard normal scale to ensure priors were sensible for all dietary factors and to increase computational stability.

Covariate effects

There were two survey-level covariate effects included in the model to explain potential bias from a survey: survey type and food definition. There were four main types of diet surveys included as covariates in the model: short-term recalls (single or multiple); food frequency questionnaires (FFQs); household budget/intake surveys; and DHS (Demographic Health Survey) questionnaires. Only the recall was considered the "gold standard" with regards to estimating the mean unbiasedly. Likewise, not all surveys used the optimal definition for a dietary factor. For example, in the case of fruits, most surveys defined fruits as all fruits. However, some surveys only measured a suboptimal metric, such as fruits including fruit juices. Currently, we combine all sub-optimal metrics into one category for our models. We also included country-year specific predictors in the model (e.g., food availability (FAO food balance sheets or Global Expanded Nutrient Supply (GENuS) model). We assumed their relationship to $f(y)$ was linear, and that the relationships were independent of location (not super-region dependent, or country dependent). See the covariate testing section for a list of the country-year specific predictors used for each dietary factor.
Mathematically, this portion of the model can be described as follows:

$$
\begin{aligned}
X_i\boldsymbol{\beta} = {} & \beta_1 I\{X_i^{\text{diet}} = \text{FFQ}\} + \\
& \beta_2 I\{X_i^{\text{diet}} = \text{household survey}\} + \\
& \beta_3 I\{X_i^{\text{diet}} = \text{DHS}\} + \\
& \beta_4 I\{X_i^{\text{metric}} = \text{alternative}\} + \\
& X_{i[j]}^{\text{country-year predictors}} \boldsymbol{\beta_c}
\end{aligned}
$$

For survey level-covariates, we used a prior of Normal(0, 0.35). The prior for β_c parameters depended on the dietary factor. For many dietary factors, we only used 1 or 2 country-level covariates, all from FAO or GENuS. For these variables, we had a very strong prior belief that the they should be strongly correlated with the outcome of interest (e.g., log(fruit availability from FAO) should be strongly positively correlated with log(fruit intake)). In these cases, we used a highly informative prior of N(1, 0.1). For other dietary factors, either no such variable exisited, or other country-year level predictors were also included and do not warrant such a high degree of certainty in a strong relationship. In these cases, we used a much weaker prior of N(0, 0.5).

Age trend

For many surveys, intake was not linearly associated with age. We modelled age using restricted cubic splines with 4 knots at k_1, k_2, k_3, k_4, corresponding to ages 5, 20, 50 and 65, respectively, after standardization:

$$\gamma_{j[i]}(z_h) = \gamma_{1j[i]} z_h + \gamma_{2j[i]} S_1 + \gamma_{3j[i]} S_2$$

where

$$S_1 = (z_h - k_1)^3_+ - \frac{k_4 - k_1}{k_4 - k_3}(z_h - k_3)^3_+ + \frac{k_3 - k_1}{k_4 - k_3}(z_h - k_4)^3_+$$

$$S_2 = (z_h - k_2)^3_+ - \frac{k_4 - k_2}{k_4 - k_3}(z_h - k_3)^3_+ + \frac{k_3 - k_2}{k_4 - k_3}(z_h - k_4)^3_+$$

As with the urban and education effect parameters, we used 2 levels of hierarchy for the age-trend:

$$\gamma_{1j[i]} = \gamma^{s}_{1k[j]} + \gamma^{g}_{1}$$
$$\gamma_{2j[i]} = \gamma^{s}_{2k[j]} + \gamma^{g}_{2}$$
$$\gamma_{3j[i]} = \gamma^{s}_{3k[j]} + \gamma^{g}_{3}$$

$$\gamma^{s}_{1k} \sim N(0, \kappa^{s}_{1\gamma}), \gamma^{s}_{2k} \sim N(0, \kappa^{s}_{2\gamma}), \gamma^{s}_{3k} \sim N(0, \kappa^{s}_{3\gamma})$$

Weakly informative distributions were used for hyper-prior parameters: Half-Normal(0, 0.5) for the κ parameters and Normal(0, 0.35) for the γ^g parameters.

Overdispersion

An additional variance component was added to each study to allow the model to account for non-sampling variation due to survey-level error (from imperfect study design and quality). This additional variance component was modeled in such a way to reflect our expectation that surveys thatare less likely to represent the true mean (but not necessarily biased) were more variable. Sources of this non-sampling variation accounted for included surveys not being nationally representative, surveys not being stratified by sex, urban/rural or education, and surveys that used large age groupings (>10 years). We also added an additional constraint to ensure local surveys were considered more variable than regional surveys.

Thus,

$$\tau_i^2 = exp(\phi_{intercept} + \phi_{regional} I(X_i^{rep} = regional) + \phi_{local} I(X_i^{rep} = local)$$
$$+ \phi_{agerange} I(X_i^{AgeRange} > 10)$$
$$+ \phi_{sex} I(X_i^{sex} = both) + \phi_{urban/rural} I((X_i^{urban/rural} = both) \text{ or } (X_i^{educ} = all))$$

with the constraints $\phi^2_{regional} < \phi^2_{local}$, and all $\phi > 0$ except $\phi_{intercept}$. We used a prior of Normal(-2.5, 1) for $\phi_{intercept}$ to reflect our a priori belief than an "ideal" survey that is both fully stratified and nationally representative should have minimal overdispersion. For all other ϕ parameters, we used a prior of Normal(0, 0.5).

Computation

We fit each model using STAN[3,4] through rstan[5], using the No-U-turn sampler (NUTS)[6], a variant of Hamiltonian Monte Carlo[7]. We used 4 chains of 2000 iterations each, treating thefirst 1000 iterations of each chain as warm up, for a total of 4000 Monte Carlo iterations to define our posterior distributions.

Predictions

The model described above was ultimately used to provide predictive distributions of mean intake for each dietary factor by country-year and subgroup. Note that the model specified $g(y_{h,i[j]})$ of

subgroup h in survey i from country j as a linear combination of model parameters and survey- year-subgroup specific information:

$$a_j + b_{1j} sex_{h,i} + b_{2s} u_{h,i} + b_{3s} educ_{h,i} + \gamma_s(z_{h,i}) + \boldsymbol{X}_i \boldsymbol{\beta}$$

where we had posterior distributions for model parameters a_j, b_j, b_{2s}, b_{3s}, γ_s and $\boldsymbol{\beta}$. To obtain a predictive distribution for subgroup h in country j, we calculated:

$$\mu_{h,j} = g^{-1}(a_j + b_{1j} sex_{h,j} + b_{2s} u_{h,j} + b_{3s} educ_{h,j} + \gamma_s(z_{h,j}) + X_j^{\text{country-year predictors}} \boldsymbol{\beta_c})$$

for each draw of our posterior distributions. Because we are interested in country-specific means, we did not use survey specific parameters in our predictions. For countries with no survey data, we did not have a posterior distribution of a_j. To get the predictive distributions for these countries in such a way that properly accounts for the variation of mean intake between countries within a region, we replaced a_j and b_{1j} with a^*_j and b^*_{1j} where $a^*_j \sim N(a_{k[j]}, \kappa^c_a)$ and $b^*_{1j} \sim N(b_{1k[j]}, \kappa^c_{1b})$. Here, $a_{k[j]}$ and $b_{1k[j]}$ are super-region-level intercepts and sex effects corresponding to country j, and κ^c and $\kappa\ b_{1k[j]}$ are super-region-level intercepts and sex effects corresponding to country j, and κ^c_a and κ^c_{1b} are the between-country variances for intercept and sex effects, respectively. In other words, each posterior draw for the super-region-level parameter and it's corresponding between-country variance parameter generated a unique normal distribution for that draw, and we took a one sample draw from each of these distributions to generate the predictive distribution of that parameter for an unknown country in that region. Note that the uncertainty around the super-region level parameter and between country variance propagate into the predictive distribution for the mean.

a

For some dietary factors, there were entire super-regions with no data. For those super-regions, predictive distributions for b_{2s}, b_{3s}, and γ_s were obtained in a similar way, generating a normal distribution for each draw from the global level parameter and between region variance parameterand sampling from that. For a_j and b_{1j}, we needed to account for between-super-region variance and the between country variance. Therefore, taking the intercept as an example, for each posterior draw, we sampled from $N(a^g, \kappa^c_a + \kappa^s_a)$. Note that this is equivalent to drawing a sample region mean from $N(a^g, k^s_a)$ then using that sample as mean and k^c_a as variance to form a normal distribution to sample country mean from.

References

1. Finucane MM, Stevens GA, Cowan MJ, et al. National, regional, and global trends in body-mass index since 1980: systematic analysis of health examination surveys and epidemiological studies with 960 country-years and 9•1 million participants. *Lancet* 2011; 377(9765):557–567.

2. Flaxman AD, Vos T, Murray C. An integrative metaregression framework for descriptive epidemiology. University of Washington Press, 2015.

3. Carpenter B, Gelman A, Hoffman MD, et al. Stan: A probabilistic programming language. *Journal of statistical software* 2017; 76(1).

4. Stan Development Team. Stan modeling language users guide and reference manual, version 2.27, 2021.

5. Stan Development Team. RStan: the R interface to Stan, 2019. R package version 2.19.2.

6. Hoffman M, Gelman A, et al. The no-u-turn sampler: adaptively setting path lengths in hamiltonian monte carlo. *Journal of Machine Learning Research* 2014; 15(1):1593–1623.

7. Neal, R. Mcmc using hamiltonian dynamics. In Brooks S, Gelman A, Jones GL, et al., Handbook of Markov Chain Monte Carlo, pages 116–162. Cambridge University Press, Cambridge, 2011.

Varying slopes modeling structure

- Our extensive work to identify surveys and model intakes led to recognition and the finding that, for certain dietary factors, the available global data and model were insufficient to accurately model differences in intakes by jointly stratified by country, age, sex, education level, and urban/rural status while also modeling differences in intakes over time.
- For countries without multiple comparable dietary surveys over time (the great majority of global nations), trends over time are largely determined by the strength of the relationship between the best available covariates (often variables from FAO food balance sheets or associated GENuS variables) and the raw survey data. For certain dietary factors, this relationship was sufficiently robust to allow modeling of all joint demographic strata and time trends. By reviewing extensive time trends plots for individual dietary factors and nations, dietary factors with a model beta coefficient ≥0.4 with their corresponding FAO/GENuS covariate were identified as having a reasonable statistical relationship to capture both all demographic strata differences and time trends. For others (FAO/GENuS beta coefficient<0.4), time trends were modeled using a second, separate Bayesian model.
- This second Bayesian model assessed the country-specific associations over time of the survey data for each dietary factor with its corresponding FAO/GENuS covariate. The model incorporated country-level intercepts and slopes, along with their correlation that is estimated across countries. Input data were the same stratified survey data as for the GDD Core model and including dietary assessment method as a covariate. This time component model did not separately estimate differences by age, sex, education, or urban/rural status, but focused on the relationships with FAO/GENuS over time. In sensitivity analyses, age and sex were included as main effects (not varying by country or region) but were found to not qualitatively alter the parameter estimates for the relationship of a country's dietary intake data with its FAO/GENuS data. Thus, including these demographics did not largely affect the time-varying predictions. This model is commonly referred as a varying slopes model structure and leverages two-dimensional partial pooling between intercepts and slopes to regularize all parameters and minimize overfitting risk[1-3]. Predictions with the varying slopes model take into account a country-specific intercept and slope when the country has dietary factor data and use the global intercept and slope for countries where data are not available. Time effects were predicted separately for each year including 1990, 1995, 2000, 2005, 2010, 2015, and 2018.
- For each country and dietary factor, the country-specific time-trend central predictions from the varying slopes models were used to generate a country-year specific adjustment scaling factor, one for each year of 1990, 1995, 2000, 2005, 2010, 2015, and 2018, compared to the reference of one of these years as determined by the median year of that country's survey data (or 2005 if no country data). This scaling factor, determined by taking the ratio of the predicted dietary intake for that year as compared to the reference year, was multiplied by the country-year posterior predictions from the fully stratified, Core GDD model to determine a time-adjusted final estimate for each stratum.
- To be conservative, this varying slopes adjustment (scaling factor) was only used for dietary factors and countries meeting all of the following criteria: at the model level, (a) FAO/GENuS beta coefficient<0.4 in the Core GDD model; and (b) availability of a closely corresponding FAO/GENuS covariate (e.g., dietary survey vitamin A intake vs. GENuS vitamin A); and at the country-level, (c) identification of a positive relationship (coefficient or slope) between the national survey data and FAO/GENuS covariate in the varying slopes model; and (d) to minimize implausible results at the country level, no more than a 3-fold difference between the ratio of the country's range of predicted intake between 1990-2018 divided by the ratio of the country's range of FAO/GENuS values over that same time period.
- Among 53 evaluated dietary factors in the GDD, 29 were modeled and incorporated time adjustment using this Bayesian varying slopes model. The other dietary factors were not because (in order of criteria applied) 11 did not have any closely corresponding FAO/GENuS variable (e.g., dietary iodine), 8 had an FAO/GENuS beta coefficient in Core GDD Core Model of at least 0.4, and 4 were unable to complete sampling for the varying-slopes model (i.e., the MCMC chains did not finalize, independent of parameterization). One additional dietary factor, vitamin B9, with a borderline FAO/GENuS beta (0.34) was also not further scaled based on adequate qualitative characteristics of the observed time trends in the GDD Core Model.

A measurement error, varying slopes model that accounts for dietary assessment method, using standardized log-intakes for all dietary factors except those reported in percent energy:

$Y_{obs,i} \sim Normal(Y_{true,i}\ , DE_{SE,i})$ [distribution for observed intake, Y_{obs}, including measurement error associated with the stratum estimate, $DE_{SE,i}$]

$Y_{true,i} \sim Normal(\mu_i\ , \sigma)$ [distribution for true strata intake Y]

$\mu_i = \alpha_{country[i]} + \beta_{country[i]} * FAO + M_{method[i]}$ [linear equation for the average intake; Each country receives its own intercept and slope while also accounting for dietary assessment method]

$\begin{bmatrix} \alpha_{country} \\ \beta_{country} \end{bmatrix} \sim MVNormal\left(\begin{bmatrix} \alpha \\ \beta \end{bmatrix}, S \right)$ [population of varying effects]

$S = \begin{pmatrix} \sigma_\alpha & 0 \\ 0 & \sigma_\beta \end{pmatrix} R \begin{pmatrix} \sigma_\alpha & 0 \\ 0 & \sigma_\beta \end{pmatrix}$ [construct covariance matrix]

With hyperpriors that define the adaptive varying effects and effects for dietary assessment:

$\alpha \sim Normal(0\ , 1)$ [prior for average intercept]

$\beta \sim Normal(1\ , 0.1)$ [prior for average slope]

$M[method] \sim Normal(0\ , 0.2)$ [prior for method effect]

$\sigma \sim Halfnormal\ (0\ ,0.5)$ [prior for stddev within countries]

$\sigma_\alpha \sim Halfnormal\ (0\ ,0.5)$ [prior for stddev among intercepts]

$\sigma_\beta \sim Halfnormal\ (0\ ,0.5)$ [prior for stddev among slopes]

$R \sim LKJcorr\ (2)$ [prior for correlation matrix]

References

1. Gelman, A. and L. Pardoe, *Bayesian measures of explained variance and pooling in multilevel (hierarchical) models.* Technometrics, 2006. **48**(2): p. 241-251.
2. McElreath, R., *Statistical Rethinking: A Bayesian Course with Examples in R and Stan.* Statistical Rethinking: A Bayesian Course with Examples in R and Stan, 2016: p. 1-464.
3. Wagner, T., et al., *Using Multilevel Models to Quantify Heterogeneity in Resource Selection.* Journal of Wildlife Management, 2011. **75**(8): p. 1788-1796.

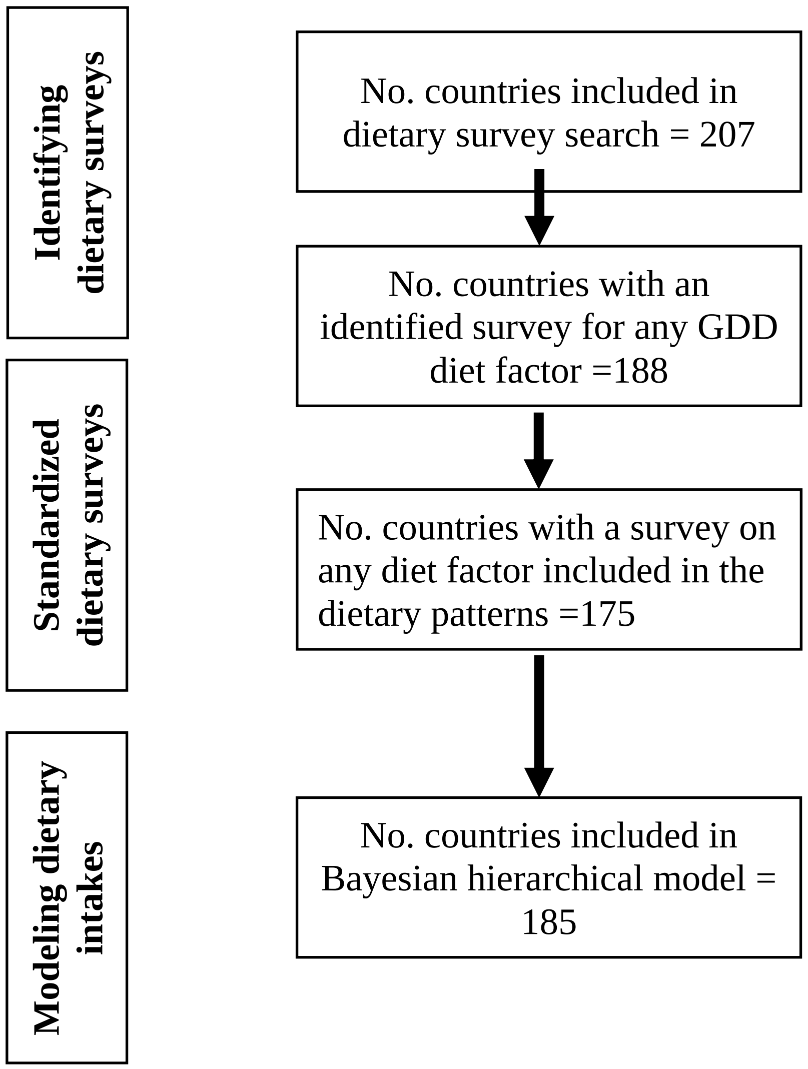

Flowchart of the number of countries with identified and standardized dietary surveys and included in the Bayesian hierarchical model.
Any GDD diet factor: fruit; non-starchy vegetables; potatoes; other starchy vegetables; beans/legumes; nuts/seeds; refined grains; whole grains; processed meats; unprocessed red meats; seafood; eggs; cheese; yogurt; sugar-sweetened beverages; fruit juices; coffee; tea; reduced fat milk; whole fat milk; total milk; energy; carbohydrate, total protein; animal protein; plant protein; saturated fat; monounsaturated fat; omega-6 fat; seafood omega-3 fat; plant omega-3 fat; dietary cholesterol; dietary fiber; added sugar; calcium; sodium; iodine; iron; magnesium; potassium; selenium; vitamin A with supplement; vitamin A without supplement; vitamin B1; vitamin B2; vitamin B3; vitamin B6; vitamin B9; vitamin C; vitamin D; vitamin E or zinc.
Any diet factor included in the dietary patterns: fruit; non-starchy vegetables; whole grains; nuts; legumes; unprocessed red meat; processed meat; seafood; cheese; yogurt; milk; sugar-sweetened beverages; fruit juice; saturated fat; monounsaturated fat; omega-6 fat; seafood omega-3 fat; plant omega-3 fat; or sodium.

Table S3. Characteristics of global data sources of dietary pattern components in children and adults.

Region (Total no. of surveys)	Number of surveys (% nationally or sub-nationally representative)	Total sample size of surveyed subjects	No. of surveys, by dietary assessment method			
			24hr recall	FFQ	DHS	Household budget survey
**Fruit (N=824)**						
Southeast and East Asia	96 (93.2)	876,659	34	58	11	0
Central/Eastern Europe and Central Asia	143 (99.3)	582,581	21	84	9	30
High Income Countries	208 (97.2)	808,110	59	118	0	37
Latin America/Caribbean	87 (91.6)	664,925	13	57	25	0
Middle East/North Africa	65 (90.3)	375,649	8	53	11	0
South Asia	33 (78.6)	713,146	10	20	12	0
Sub-Saharan Africa	148 (96.1)	1,148,611	13	59	82	0
**Overall**	**780 (94.7)**	**5,169,681**	**158**	**449**	**150**	**67**
**Non-starchy vegetables (N=798)**						
Southeast and East Asia	93 (93.0)	870,168	34	56	10	0
Central/Eastern Europe and Central Asia	138 (99.3)	566,339	19	84	6	30
High Income Countries	205 (97.2)	779,398	58	116	0	37
Latin America/Caribbean	83 (92.2)	654,787	12	55	23	0
Middle East/North Africa	63 (90.0)	369,112	7	53	10	0
South Asia	33 (80.5)	693,550	10	19	12	0
Sub-Saharan Africa	142 (96.6)	1,129,112	12	56	79	0
**Overall**	**757 (94.9)**	**5,082,466**	**152**	**439**	**140**	**67**
**Whole grains (N=256)**						
Southeast and East Asia	21 (91.3)	140,021	6	17	0	0
Central/Eastern Europe and Central Asia	47 (100.0)	111,346	11	19	0	17
High Income Countries	118 (96.7)	410,674	34	51	0	37
Latin America/Caribbean	13 (81.3)	122,474	10	6	0	0
Middle East/North Africa	10 (66.7)	46,519	7	8	0	0
South Asia	7 (50.0)	70,770	9	5	0	0
Sub-Saharan Africa	16 (84.2)	82,455	11	2	6	0
**Overall**	**162 (89.0)**	**986,259**	**88**	**108**	**6**	**54**

Region<br>(Total no. of surveys)	Number of surveys<br>(% nationally or sub-nationally representative)	Total sample size of surveyed subjects	No. of surveys, by dietary assessment method			
			24hr recall	FFQ	DHS	Household budget survey
**Nuts (N=273)**						
Southeast and East Asia	39 (88.6)	537,352	32	11	1	0
Central/Eastern Europe and Central Asia	51 (100.0)	246,692	12	9	1	29
High Income Countries	115 (97.4)	327,010	51	31	0	36
Latin America/Caribbean	13 (76.5)	161,970	10	5	2	0
Middle East/North Africa	15 (75.0)	116,837	7	9	4	0
South Asia	4 (44.4)	303,036	8	0	1	0
Sub-Saharan Africa	10 (71.4)	39,203	10	1	3	0
**Overall**	**247 (90.1)**	**1,732,100**	**130**	**66**	**12**	**65**
**Legumes (N=385)**						
Southeast and East Asia	39 (86.7)	470,491	13	22	10	0
Central/Eastern Europe and Central Asia	46 (97.9)	249,981	11	1	5	30
High Income Countries	94 (94.0)	245,232	55	12	0	36
Latin America/Caribbean	42 (89.4)	513,736	12	13	22	0
Middle East/North Africa	19 (76.0)	180,240	7	11	7	0
South Asia	21 (75.0)	641,215	10	6	12	0
Sub-Saharan Africa	88 (94.6)	996,271	13	4	76	0
**Overall**	**348 (90.4)**	**3,297,166**	**118**	**69**	**132**	**66**
**Unprocessed red meat (N=411)**						
Southeast and East Asia	57 (89.1)	709,036	34	23	7	0
Central/Eastern Europe and Central Asia	64 (98.5)	279,723	17	10	8	30
High Income Countries	98 (94.2)	248,332	54	13	0	37
Latin America/Caribbean	40 (87.0)	491,937	12	11	23	0
Middle East/North Africa	22 (75.9)	205,057	7	13	9	0
South Asia	18 (66.7)	390,182	10	8	9	0
Sub-Saharan Africa	70 (92.1)	769,897	13	3	60	0
**Overall**	**369 (89.8)**	**3,094,164**	**147**	**81**	**116**	**67**
**Processed meat (N=224)**						

Region (Total no. of surveys)	Number of surveys (% nationally or sub-nationally representative)	Total sample size of surveyed subjects	No. of surveys, by dietary assessment method			
			24hr recall	FFQ	DHS	Household budget survey
Southeast and East Asia	26 (86.7)	335,908	10	20	0	0
Central/Eastern Europe and Central Asia	47 (100.0)	206,263	16	1	0	30
High Income Countries	94 (94.9)	244,319	53	9	0	37
Latin America/Caribbean	16 (72.7)	137,178	12	9	1	0
Middle East/North Africa	11 (68.8)	44,395	8	8	0	0
South Asia	2 (50.0)	25,629	2	2	0	0
Sub-Saharan Africa	5 (83.3)	5,916	4	2	0	0
**Overall**	**201 (89.7)**	**999,608**	**103**	**53**	**1**	**67**
**Seafood (N=341)**						
Southeast and East Asia	53 (88.3)	684,216	32	22	6	0
Central/Eastern Europe and Central Asia	52 (98.1)	258,984	16	1	6	30
High Income Countries	84 (94.4)	233,332	41	11	0	37
Latin America/Caribbean	28 (84.8)	305,259	9	14	10	0
Middle East/North Africa	18 (75.0)	156,174	6	12	6	0
South Asia	13 (68.4)	377,102	6	6	7	0
Sub-Saharan Africa	59 (93.7)	708,490	10	4	49	0
**Overall**	**307 (90.0)**	**2,723,557**	**120**	**70**	**84**	**67**
**Cheese (N=144)**						
Southeast and East Asia	8 (80.0)	240,567	3	5	2	0
Central/Eastern Europe and Central Asia	11 (91.7)	60,497	7	1	4	0
High Income Countries	24 (85.7)	175,493	21	7	0	0
Latin America/ Caribbean	20 (80.0)	297,612	8	3	14	0
Middle East/ North Africa	11 (64.7)	118,768	4	9	4	0
South Asia	6 (85.7)	71,524	2	1	4	0
Sub-Saharan Africa	44 (97.8)	594,219	4	1	40	0
**Overall**	**124 (86.1)**	**1,558,680**	**49**	**27**	**68**	**0**
**Yogurt (N=191)**						
Southeast and East Asia	25 (92.6)	388,355	5	14	8	0

Region (Total no. of surveys)	Number of surveys (% nationally or sub-nationally representative)	Total sample size of surveyed subjects	No. of surveys, by dietary assessment method			
			24hr recall	FFQ	DHS	Household budget survey
Central/Eastern Europe and Central Asia	13 (100.0)	61,317	7	0	6	0
High Income Countries	24 (8.9)	175,369	21	6	0	0
Latin America/Caribbean	20 (83.3)	364,577	8	5	11	0
Middle East/North Africa	12 (70.6)	155,358	5	6	6	0
South Asia	10 (83.3)	357,578	3	1	8	0
Sub-Saharan Africa	70 (98.6)	870,272	3	0	68	0
**Overall**	**174 (91.1)**	**2,372,826**	**52**	**32**	**107**	**0**
**Milk (N=446)**						
Southeast and East Asia	60 (92.3)	648,419	32	19	14	0
Central/Eastern Europe and Central Asia	67 (98.5)	281,878	20	10	10	28
High Income Countries	92 (95.8)	193,691	52	7	0	37
Latin America/Caribbean	52 (96.3)	543,624	7	8	39	0
Middle East/North Africa	20 (90.9)	190,580	4	7	11	0
South Asia	23 (79.3)	647,491	9	6	14	0
Sub-Saharan Africa	108 (96.4)	1,046,581	12	2	98	0
**Overall**	**422 (94.6)**	**3,554,611**	**136**	**59**	**186**	**65**
**Sugar-sweetened beverages (N=459)**						
Southeast and East Asia	38 (86.4)	394,432	13	30	1	0
Central/Eastern Europe and Central Asia	90 (100.0)	313,350	15	58	0	17
High Income Countries	205 (97.6)	802,300	57	116	0	37
Latin America/Caribbean	39 (90.7)	272,328	10	28	5	0
Middle East/North Africa	33 (84.6)	186,987	7	27	5	0
South Asia	7 (70.0)	82,809	3	5	2	0
Sub-Saharan Africa	19 (82.6)	78,950	8	12	3	0
**Overall**	**431 (94.0)**	**2,131,156**	**113**	**276**	**16**	**54**
**Fruit juice (N=355)**						
Southeast and East Asia	25 (83.3)	395,588	7	12	11	0
Central/Eastern Europe and Central Asia	53 (100.0)	246,691	17	0	7	29

Region (Total no. of surveys)	Number of surveys (% nationally or sub-nationally representative)	Total sample size of surveyed subjects	No. of surveys, by dietary assessment method			
			24hr recall	FFQ	DHS	Household budget survey
High Income Countries	93 (94.9)	220,839	53	11	0	34
Latin America/Caribbean	49 (94.2)	532,700	9	6	37	0
Middle East/North Africa	17 (81.0)	156,711	6	6	9	0
South Asia	8 (72.7)	299,960	3	1	7	0
Sub-Saharan Africa	88 (97.8)	884,811	4	3	83	0
**Overall**	**333 (93.8)**	**2,736,800**	**99**	**39**	**154**	**63**
**Monounsaturated fat (N=91)**						
Southeast and East Asia	9 (64.3)	224,884	7	7	0	0
Central/Eastern Europe and Central Asia	6 (85.7)	18,415	6	1	0	0
High Income Countries	23 (85.2)	173,510	20	7	0	0
Latin America/Caribbean	8 (50.0)	124,863	11	5	0	0
Middle East/North Africa	8 (53.3)	30,222	7	8	0	0
South Asia	1 (14.3)	45,208	4	3	0	0
Sub-Saharan Africa	3 (60.0)	3,357	3	2	0	0
**Overall**	**58 (63.7)**	**620,459**	**58**	**33**	**0**	**0**
**Saturated fat (N=198)**						
Southeast and East Asia	18 (72.0)	262,377	12	13	0	0
Central/Eastern Europe and Central Asia	25 (96.2)	35,275	23	3	0	0
High Income Countries	82 (91.1)	231,760	73	17	0	0
Latin America/Caribbean	15 (65.2)	130,018	13	10	0	0
Middle East/North Africa	9 (50.0)	32,462	9	9	0	0
South Asia	1 (10.0)	48,473	7	3	0	0
Sub-Saharan Africa	4 (66.7)	4,357	3	3	0	0
**Overall**	**154 (77.8)**	**744,722**	**140**	**58**	**0**	**0**
**Seafood omega-3 fat (N=178)**						
Southeast and East Asia	12 (75.0)	235,347	6	10	0	0
Central/Eastern Europe and Central Asia	43 (100.0)	191,442	13	0	0	30
High Income Countries	83 (97.6)	189,608	39	9	0	37

Region (Total no. of surveys)	Number of surveys (% nationally or sub-nationally representative)	Total sample size of surveyed subjects	No. of surveys, by dietary assessment method			
			24hr recall	FFQ	DHS	Household budget survey
Latin America/Caribbean	8 (88.9)	11,109	5	4	0	0
Middle East/North Africa	10 (58.8)	30,484	7	10	0	0
South Asia	2 (50.0)	2,390	4	0	0	0
Sub-Saharan Africa	4 (100.0)	1,450	3	1	0	0
**Overall**	**162 (91.0)**	**663,564**	**77**	**34**	**0**	**54**
**Plant omega-3 fat (N=74)**						
Southeast and East Asia	12 (80.0)	236,290	7	8	0	0
Central/Eastern Europe and Central Asia	6 (100.0)	9,265	6	0	0	0
High Income Countries	32 (97.0)	139,758	25	8	0	0
Latin America/Caribbean	6 (100.0)	9,599	3	3	0	0
Middle East/North Africa	5 (50.0)	25,335	6	4	0	0
South Asia	0 (0.0)	2,040	2	0	0	0
Sub-Saharan Africa	1 (50.0)	1,944	1	1	0	0
**Overall**	**62 (83.8)**	**424,231**	**50**	**24**	**0**	**0**
**Omega-6 fat (N=126)**						
Southeast and East Asia	14 (70.0)	254,326	11	9	0	0
Central/Eastern Europe and Central Asia	11 (91.7)	13,355	11	1	0	0
High Income Countries	49 (94.2)	158,754	41	11	0	0
Latin America/Caribbean	10 (58.8)	27,601	10	7	0	0
Middle East/North Africa	9 (52.9)	30,982	8	9	0	0
South Asia	0 (0.0)	22,765	2	3	0	0
Sub-Saharan Africa	2 (66.7)	2,494	2	1	0	0
**Overall**	**95 (75.4)**	**510,777**	**85**	**41**	**0**	**0**
**Sodium (N=394)***						
Southeast and East Asia	56 (71.8)	351,306	34	19	0	0
Central/Eastern Europe and Central Asia	33 (94.3)	185,042	14	2	0	13
High Income Countries	149 (84.7)	226,622	83	11	0	0
Latin America/Caribbean	21 (67.7)	179,667	14	6	0	1

Region (Total no. of surveys)	Number of surveys (% nationally or sub-nationally representative)	Total sample size of surveyed subjects	No. of surveys, by dietary assessment method			
			24hr recall	FFQ	DHS	Household budget survey
Middle East/North Africa	18 (64.3)	47,053	12	14	0	0
South Asia	6 (33.3)	91,572	11	5	0	0
Sub-Saharan Africa	11 (39.3)	12,438	8	3	0	0
**Overall**	**294 (74.6)**	**1,093,700**	**177**	**60**	**0**	**14**

1,139 surveys on fruit, non-starchy vegetables, whole grains, nuts, legumes, unprocessed red meat, processed meat, seafood, cheese, yogurt, milk, sugar-sweetened beverages, fruit juice, monounsaturated fat, saturated fat, seafood omega-3 fat, plant omega-3 fat, omega-6 fat, or sodium. 30.6% of surveys from High-Income Countries; 17.4% from Sub-Saharan Africa; 14.1% from Central/Eastern Europe and Central Asia; 13.8% from Southeast and East Asia; 11.3% from Latin America/Caribbean; 7.8% from Middle East/North Africa; 5.0% from South Asia.

*143 Biomarker surveys available: Southeast and East Asia=25; Central/Eastern Europe and Central Asia=6; High Income Countries=82; Latin America/Caribbean=9; Middle East/North Africa=2; South Asia=2; Sub-Saharan Africa=17.

Table S4. Characteristics of total dietary surveys (N=1,139) used to model the dietary pattern components.

Survey characteristics	Number of surveys
Surveys assessed and included, n	1139
Number of countries represented, n	175
Global population represented, n (%)	7.46 of 7.63 billion (97.8)
Demographic characteristics reported by surveys, %	
Children/adolescents (ages 0-19)	73.9
<1 year	26.1
1 to 2 years	28.5
3 to 4 years	23.3
5 to 9 years	24.1
10 to 14 years	49.6
15 to 19 years	62.2
Adults (ages 20+)	64.5
20 to 24 years	50.7
25 to 29 years	57.2
30 to 34 years	57.3
35 to 39 years	58.4
40 to 44 years	61.5
45 to 49 years	54.9
50 to 54 years	53.4
55 to 59 years	42.6
60 to 64 years	35.6
65 to 69 years	34.4
70 to 74 years	30.6
75 to 79 years	27.8
80 to 84 years	25.5
85 to 89 years	23.9
90 to 94 years	22.4
≥95 years	20.5
Level of education	30.2
Urban vs. rural residence	

Both urban and rural	54.5
Urban only	4.4
Rural only	1.8
Information not available	39.2
Representativeness, %	
National	70.9
Subnational	18.3
Community	10.9
Response rate, %	
60 to 100%	37.3
20 to 59%	3.2
< 20 %	6.0
Information not available	53.5
Sampling methodology, %	
Probability sampling, including survey weights	38.6
Probability sampling without survey weights	33.2
Non-probability sampling	3.9
Information not available	24.3
Dietary assessment method, %	
Single or multiple diet recalls/records	22.7
Food frequency questionnaire	42.1
DHS questionnaire	16.7
Biomarker	12.6
Level of data collection, %	
Individual-level	91.6
Household-level	6.1
Information not available	2.3
Data type, %	
Individual-level dietary data	60.8
Aggregated stratum-level distributions	39.2

To calculate the population represented, we assumed that one or more surveys for any year of data collection were representative of the national population. We summed the national populations of the countries with survey data to estimate the absolute population represented. For the estimate of the percentage of the population represented, we used 7.63 billion as the denominator (global population in 2018).

Table S5. Number of dietary surveys for diet factor overall and by year.

Dietary factor	Total no. surveys	No. surveys by year							
		1980-1984	1985-1989	1990-1994	1995-1999	2000-2004	2005-2009	2010-2014	≥2015
Any diet factor	1139	29	95	94	174	268	268	198	15
Fruit	824	4	28	55	95	221	228	178	15
Non-starchy vegetables	798	4	25	53	90	211	222	178	15
Whole grains	256	2	23	43	61	46	41	32	8
Nuts	273	3	22	48	44	59	54	34	9
Legumes	385	3	13	30	45	92	92	96	14
Milk	446	3	23	41	83	106	87	88	15
Unprocessed red meat	411	3	13	35	71	80	93	101	15
Seafood	341	2	12	27	49	56	83	97	15
Processed meat	224	3	12	30	41	56	46	28	8
Yogurt	191	0	1	5	7	27	59	79	13
Cheese	144	0	0	5	4	13	40	70	12
Sugar-sweetened beverages	459	3	26	52	72	86	100	112	8
Fruit juice	355	1	18	43	64	79	57	80	13
Monounsaturated fat	91	0	0	7	5	7	28	37	7
Saturated fat	198	1	5	25	29	47	45	39	7
Seafood omega-3 fat	178	2	11	26	47	46	28	13	5
Plant omega-3 fat	74	0	0	8	14	15	18	14	5
Omega-6 fat	126	0	4	14	22	25	28	30	3
Sodium	394	25	60	38	75	71	69	47	8

Any diet factor: fruit; non-starchy vegetables; whole grains; nuts; legumes; unprocessed red meat; processed meat; seafood; cheese; yogurt; milk; sugar-sweetened beverages; fruit juice; monounsaturated fat; saturated fat; seafood omega-3 fat; plant omega-3 fat; omega-4 fat; or sodium.

The Global Dietary Database Corresponding Members

Pamela Abbott; Morteza Abdollahi, National Nutrition and Food Technology Research Institute (NNFTRI): SBMU; Parvin Abedi, Ahvaz Jundishapur University of Medical Sciences; Suhad Abumweis, The Hashemite University; Linda Adair; Swee Ai Ng; Mohannad Al Nsour, Eastern Mediterranean Public Health Network: EMPHNET; Iftikhar Alam, BKUC.edu.pk; Nasser Al-Daghri, King Saud University; Nawal Al-Hamad, Ministry of Health; Suad Al-Hooti, Kuwait Institute for Scientific Research; Eman Alissa, King Abdulaziz University; Sameer Al-Zenki; Simon Anderson, University of Manchester; Karim Anzid, Cadi Ayyad University; Carukshi Arambepola, Faculty of Medicine, University of Colombo, Sri Lanka; Mustafa Arici, Hacettepe University Faculty of Medicine; Joanne Arsenault, University of California, Davis; Renzo Asciak; Lajos Biró; Noël Barengo, Herbert Wetheim College of Medicine; Simon Barquera, Instituto Nacional de Salud Publica; Murat Bas, Acibadem University; Wulf Becker; Sigrid Beer-Borst; Per Bergman, Livsmedelsverket; Sesikeran Boindala, National Institute of Nutrition India; Pascal Bovet, Intitute of Soical and Preventive Medicine, Univeresity of Lausanne, Switzerland AND Ministry of Health, Republic of Seychelles; Debbie Bradshaw; Noriklil Bukhary Ismail Bukhary, Ministry of Health (Malaysia); Kanitta Bundhamcharoen; Mauricio Caballero, Fundacion Infant; Neville Calleja, Directorate for Health Information & Research; Xia Cao; Mario Capanzana, Food and Nutrition Research Institute, Dept. of Science and Technology; Jan Carmikle, Senior Intellectual Property Office; Katia Castetbon, Institut de Veille Sanitaire; Michelle Castro, Departamento de Alimentação Escolar; Corazon Cerdena; Hsing-Yi Chang, National Health Research Institutes; Karen Charlton; Yu Chen, NYU School of Medicine; Shashi Chiplonkar, HC Jehangir Medical Research Institute, Pune India; Yoonsu Cho, Korea University; Khun-Aik Chuah; Simona Costanzo, IRCCS INM Neuromed; Melanie Cowan; Albertino Damasceno, Faculty of Medicine, Eduardo Mondlane University, Maputo, Mozambique; Saeed Dastgiri, Tabriz University of Medical Sciences; Stefaan De Henauw, Ghent University; Karin DeRidder, Belgian Public Health Institute; Eric Ding, Harvard School of Public Health; Rivera Dommarco; Rokiah Don; Charmaine Duante; Vesselka Duleva, Head of Department; Samuel Duran Aguero, Universidad San Sebastian, Chile; Veena Ekbote, Hirabai Cowasji Jehangir Medical Research Institute, Jehangir Hospital, Pune, India; Jalila El Ati, National Institute of Nutrition and Food Technology & SURVEN RL; Asmaa El Hamdouchi; Alison Eldridge, Nestle Research Center; Tatyana El-kour, World Health Organization; Ibrahim Elmadfa, University of Vienna; Helene Enghardt Barbieri; Alireza Esteghamati, Tehran University of Medical Sciences; Zohreh Etemad, Dutch National Institute for Public Health and the Environment (RIVM); Fariza Fadzil, Ministry of Health, Malaysia; Farshad Farzadfar; Mei Fen Chan; Anne Fernandez, Perdana University, Royal College of Surgeons in Ireland; Dulitha Fernando; Regina Fisberg, University of Sao Paulo, Brazil; Simon Forsyth, The University of Queensland School of Public Health; Edna Gamboa Delgado, Fundacion Cardiovascular de Colombia; Didier Garriguet, Statistics Canada; Jean-Michel Gaspoz; Dorothy Gauci; Marianne Geleijnse, Wageningen University; Brahmam Ginnela; Giuseppe Grosso, Integrated Cancer Registry of CT-ME-SR-EN; Idris Guessous, Geneva University Hospitals; Martin Gulliford, King's College London; Ingibjorg Gunnarsdottir; Wilbur Hadden; Aida Hadziomeragic, Institute of Public Health of Federation of Bosnia and Herzegovina; Christian Haerpfer; Jemal Haidar Ali, Addis Ababa University; Rubina Hakeem; Aminul Haque, University of Dhaka; Maryam Hashemian; Rajkumar Hemalatha, Director - ICMR-National Institute of Nutrition; Sigrun Henjum, Oslo and Akerhus University College; Hristo Hinkov, Director of National Center of Public Health and Analyses (NCPHA); Zaiton Hjdaud; Daniel Hoffman, Rutgers University; Beth Hopping; Anahita Houshiar-rad, National Nutrition & Food Technology Research Ins; Yao-Te Hsieh, Institute of Biomedical Sciences, Academia Sinica, Taipei, Taiwan; Shu-Yi Hung; Inge Huybrechts, International Agency for Research on Cancer; Nahla Chawkat Hwalla, American University of Beirut; Nayu Ikeda; Daniel Illescas-Zarate, National Institute of Public Health; Manami Inoue; Olof Jonsdottir; Hamid Jan Bin Jan Mohamed, Universiti Sains Malaysia; Chandrashekar Janakiram, Amrita School of Dentistry; Ranil Jayawardena, University of Colombo; Rajesh Jeewon, University of Mauritius; Nattinee Jitnarin; Lars Johansson; Ola Kally; Mirnalini Kandiah; Tilakavati Karupaiah, National University of Malaysia; Lital Keinan-Boker, Israel Center for Disease Control; Roya Kelishadi, Research Institute for Primordial Prevention of NCD, Isfahan University of Medical Sciences; Anuradha Khadilkar, Hirabai Cowasji Jehangir Medical Research Institute; Cho-il Kim, Korea Health Industry

Development Institutee; Eda Koksal; Jurgen Konig, University of Vienna, Department of Nutritional Sciences; Liisa Korkalo, University of Helsinki; Jeremy Koster, University of Cincinnati; Irina Kovalskys, ILSI (International Life Sciences Institute), Argentina; Anand Krishnan, All India Institute of Medical Sciences; Herculina Kruger, North-West University, Potchefstroom South Africa; Rebecca Kuriyan-Raj, St. John's Research Institute; Sanghui Kweon; Carl Lachat, Ghent University, Belgium; Yuen Lai; Pulani Lanerolle, University of Colombo, Sri Lanka; Indu Waidyatilaka, University of Colombo, Sri Lanka; Avula Laxmaiah, National Institute of Nutrition, ICMR, Hyderabad; Catherine Leclercq; Meei-Shyuan Lee; Hae-Jeung Lee, Eulji University; J Lennert Veerman, The University of Queensland; Lydia Lera Marques, Unidad de Nutricion Publica-Professor Asociado; Yanping Li, Harvard School of Public Health; Jaana Lindström; Annie Ling; Nur Indrawaty Liputo, Andalas University; Patricio Lopez-Jaramillo, FOSCAL and UDES; Amy Luke, Loyola University Chicago; Widjaja Lukito; Nuno Lunet, Faculty of Medicine, University of Porto, Portugal; Elisabette Lupotto, Director of CREA-Alimenti e Nutrizione; Guansheng Ma; Yi Ma; Zaleha Abdullah Mahdy, National University of Malaysia (UKM); Reza Malekzadeh, Digestive Digestive Center, Digestive Disease Research Institute,Tehran University of Medical Sciences; Wan Manan, Universiti Sains Malaysia; Dirce Marchioni; Pedro Marques-Vidal, Lausanne University Hospital (CHUV); Yves Martin-Prevel, Institut de Recherche pour le Developpement; Hajah Masni Ibrahim; Angie Mathee; Yasuhiro Matsumura, Bunkyo University, Faculty of Health and Nutrition; Paramita Mazumdar, Centre For Media Studies; Abla Mehio Sibai; Anjum Memon, Brighton and Sussex Medical School; Gert Mensink, Robert Koch Institute; Alexa Meyer, University of Vienna, Austria; Parvin Mirmiran, Research Institute for Endocrine Sciences, Shahid Beheshti University of Medical Sciences;Masoud Mirzaei, Yazd Cardiovascular Research Centre, Shahid Sadoughi University of Medical Sciences; Puneet Misra, All India Indtitute of Medical Sciences; Anoop Misra, Fortis CDOC Center for Excellence for Diabetes; Claudette Mitchell, University of the Southern Caribbean; Noushin Mohammadifard, Isfahan Cardiovascular Research Center, Cardiovascular Research Center, Isfahan University of Medical Sciences; Fatemeh Mohammadi-Nasrabadi, National Nutrition and Food technology Reserch Institute; Zalilah Mohd Shariff, Universiti Putra Malaysia; Foong Ming Moy, University of Malaya; Abdulrahman Musaiger, Arab Center for Nutrition; Elizabeth Mwaniki, The Technical University of Kenya; Jannicke Myhre; Balakrishna Nagalla; Androniki Naska,; Augustin Nawidimbasba Zeba, Intitut de Recherche en sciences de la Sante; Shu Wen Ng, University of North Carolina at Chapel Hill; Le Tran Ngoan, Hanoi Medical University; Sina Noshad, Tehran University of Medical Sciences; Angelica Ochoa, Universidad de Cuenca; Marga Ocke, Dutch National Institute for Public Health and the Environment: RIVM; Jillian Odenkirk; Kyungwon Oh, Director, Division of Health and Nutrition Survey; Mariana Oleas, Universidad Tecnica del Norte; Sonia Olivares, Institute of Nutrition and Food Technology (INTA), University of Chile; Philippos Orfanos; Johana Ortiz-Ulloa, Cuenca University; Johanna Otero, Fundacion Oftalmologica de Santander (FOSCAL); Marja-Leena Ovaskainen; Mohammadreza Pakseresht; Cristina Palacios, Florida International University; Pam Palmer; Wen-Harn Pan; Demosthenes Panagiotakos, Harokopio University; Rajendra Parajuli, McGill University; Myungsook Park; Gulden Pekcan; Stefka Petrova; Noppawan Piaseu, Mahidol University; Christos Pitsavos; Kalpagam Polasa, National Institute of Nutrition; Luz Posada, Universidad de Antioquia; Farhad Pourfarzi, Ardabil University of Medical Sciences; Alan Martin Preston, Univ Puerto Rico-Med Sci Dept Biochemistry; Ingrid Rached, Centro de Atencion Nutricional Antimano (CANIA); Ali Reza Rahbar; Colin Rehm; Almut Richter; Leanne Riley; Luz Maria Sánchez-Romero, National Institute of Public Health Mexico; Benoit Salanave; Nizal Sarrafzadegan, Professor- Isfahan Cardiovascular Research Center; Norie Sawada, Research Center for Cancer Prevention and Screening, National Cancer Center; Makiko Sekiyama, Graduate Program in Sustainability Science Global Leadership Initiative (GPSS—GLI), The University of Tokyo; Rusidah Selamat; Khadijah Shamsuddin, Universiti Kebangsaan Malaysia Medical Centre; Sangita Sharma, University of Alberta; Harri Sinkko; Isabelle Sioen; Ivan Sisa, USFQ; Sheila Skeaff, University of Otago; Laufey Steingrimsdottir; Tor Strand, University of Bergen; Milton Fabian Suarez-Ortegon, University of Edinburgh; Sumathi Swaminathan, St John's Research Institute; Gillian Swan; Elzbieta Sygnowska; Maria Szabo; Lucjan Szponar, National Food and Nutrition Institute; Ilse Tan-Khouw; Heli Tapanainen, The National Institute for Health and Welfare (THL); Reema Tayyem, the Hashemite University; Bemnet Tedla; Alison Tedstone; Robert Templeton; Celine Termote, Bioversity International; Anastasia Thanopoulou, Diabetes Center, 2nd Department of internal Medicine, Athens University, Greece; Holmfridur Thorgeirsdottir; Inga Thorsdottir; Dimitrios

Trichopoulos; Antonia Trichopoulou; Shoichiro Tsugane; Aida Turrini; Coline van Oosterhout; Erkki Vartiainen,; Suvi Virtanen, National Institute for Health and Welfare; Peter Vollenweider; Marieke Vossenaar, CeSSIAM in Guatemala; Eva Warensjo Lemming, Risk and Benefit assessment Department, National Food Agency Sweden; Anna Waskiewicz, Department of CVD Epidemiology, Prevention and Health Promotion, Institute of Cardiology, Warsaw, Poland; Eveline Waterham; Lothar Wieler, Robert Koch Institute; Tizita Wondwossen, Addis Ababa University; Suh Wu; Roseyati Yaakub; Mabel Yap,; Safiah Yusof; Sahar Zaghloul; Gábor Zajkás; Maria Zapata, CESNI; Khairul Zarina; Fatemeh Vida Zohoori, Teesside University

Table S6. AHEI scoring thresholds.

Score	0	10
Fruit, servings/d	0.00	4.00
Non-starchy vegetables, servings/d	0.00	5.00
Whole grains, women, g/d	0.00	75.00
Whole grains, men, g/d	0.00	90.00
Sugar-sweetened beverages and fruit juice, servings/d	1.00	0.00
Legumes and nuts, servings/d	0.00	1.00
Unprocessed red meat and processed meat, servings/d	1.50	0.00
Seafood omega-3 fat, mg/d	0.00	250.00
PUFAs, % energy/d	2.00	10.00
Sodium*, mg/d	10292.65	1657.16

*Sodium deciles for 2018 were applied to 1990.

The AHEI included 9 of the 11 AHEI components (alcohol and *trans*-fat were excluded): non-starchy vegetables, fruits, nuts and legumes, whole grains, red and processed meat, sugar-sweetened beverages and fruit juice, polyunsaturated fatty acids (PUFAs), long-chain n-3 PUFAs, and sodium. Each component was scored from 0 to 10, and the AHEI ranged from 0 (non-adherence) to 90 (perfect adherence) but was scaled to range from 0 to 100.

Table S7. DASH scoring thresholds.

	Women				Men			
**Percentile**	**20%**	**40%**	**60%**	**80%**	**20%**	**40%**	**60%**	**80%**
Fruit, servings/d	0.71	0.98	1.26	1.75	0.65	0.86	1.11	1.52
Non-starchy vegetables, servings/d	0.94	1.35	1.74	2.29	0.86	1.24	1.59	2.13
Legumes and nuts, servings/d	0.25	0.41	0.60	0.93	0.26	0.41	0.60	0.95
Whole grains, servings/d	0.27	0.47	0.77	1.30	0.26	0.46	0.75	1.29
Low fat dairy, servings/d	0.34	0.66	1.12	1.71	0.31	0.62	1.07	1.64
Red and processed meat, servings/d	0.37	0.62	0.97	1.49	0.38	0.66	1.10	1.66
Sugar-sweetened beverages, servings/d	0.17	0.37	0.64	1.08	0.19	0.40	0.68	1.14
Sodium, mg/d	1857.58	2233.79	2615.16	3037.78	1974.49	2374.67	2814.69	3228.36

The DASH included 8 components: non-starchy vegetables, fruits, nuts and legumes, whole grains, low fat dairy products, red and processed meat, sugar-sweetened beverages, and sodium. Each component was scored from 1 to 5 using sex-specific quintiles, and the DASH score ranged from 8 (non-adherence) to 40 (perfect adherence).

Table S8. MED scoring thresholds.

	Median cutoff	
	**Women**	**Men**
Fruit and nuts, servings/d	1.45	1.29
Non-starchy vegetables, servings/d	1.54	1.41
Legumes, servings/d	0.22	0.23
Whole grains, servings/d	0.61	0.59
Dairy, servings/d	0.89	0.84
Red and processed meat, servings/d	0.80	0.86
Seafood, servings/d	0.31	0.30
MUFA:SFA	0.98	1.05

The MED included 8 of the 9 MED components (alcohol was excluded): non-starchy vegetables, fruits and nuts, legumes, whole grains, dairy products, red and processed meat, seafood, and the ratio of monounsaturated fatty acids to saturated fatty acids. Each component was scored as 0 or 1 using sex-specific medians, and the MED score ranged from 0 (non-adherence) to 8 (perfect adherence).

Global, regional, and national DASH and MED scores in 2018
The global mean DASH score in 2018 was 22.9 (95% UI 22.6, 23.2). Regional means were highest in the Central/Eastern Europe and Central Asia (24.7; 23.9, 25.5) and South Asia (24.5; 24.0, 25.1), and lowest in Latin America and the Caribbean (20.3; 19.7, 20.8) and Southeast and East Asia (21.7; 21.2, 22.1). Among populous countries, the mean DASH score ranged between 18.2 (17.5, 18.8) to 28.5 (27.9, 29.2). Highest DASH scores were observed in Iran, Vietnam, Russia, and India, while lowest scores were identified in Brazil, Pakistan, the Philippines, and Egypt.

Globally, the mean MED score in 2018 was 4.1 (95% UI 3.9, 4.2). High-Income Countries and Latin American and the Caribbean had the lowest regional MED scores (2.8; 2.6, 2.9 and 3.0; 2.8, 3.2, respectively), and South Asia and Sub-Saharan Africa had the highest (5.0; 4.5, 5.3 and 4.7; 4.4, 5.0, respectively). Among the most populous countries, the national MED score was lowest the United States, the Philippines, Germany, and Brazil (range 2.4 to 2.5), and highest in Bangladesh, Tanzania, the Democratic Republic of Congo, and Vietnam (range 5.4 to 6.1).
